# Current Management and the New Paradigm for ALK+ NSCLC After CROWN

**DOI:** 10.3390/cancers18183044

**Published:** 2026-09-19

**Authors:** Shuo Shi, Petros Christopoulos

**Affiliations:** 1 Department of Medical Oncology, Thoraxklinik, Heidelberg University Hospital and National Center for Tumor Diseases (NCT) Heidelberg, a Partnership Between the German Cancer Research Center (DKFZ) and Heidelberg University Hospital, 69126 Heidelberg, Germany; shuo.shi@med.uni-heidelberg.de; 2 Department of Translational Pulmonology, Translational Lung Research Center at Heidelberg University Hospital, German Center for Lung Research (DZL), 69120 Heidelberg, Germany

**Keywords:** ALK+ NSCLC, ALK inhibitors, lorlatinib, tyrosine kinase inhibitors

## Abstract

The 7-year update of the phase 3 CROWN study is the most impressive milestone in anaplastic lymphoma kinase (ALK)+ non-small-cell lung cancer (NSCLC) since the discovery of the oncogene 20 years ago and inaugurates an entire new paradigm. The progression-free survival (PFS) plateau at a high level > 50%, with an estimated very low annual progression rate < 2% after the third year, suggests that lorlatinib can inactivate the disease in most patients. The complete abrogation of on-target resistance abolishes ALK tyrosine kinase inhibitor (TKI) sequencing and will seriously impact future drug development. In the new era of extraordinary drug efficacy, the design of clinical trials, pathways of marketing authorization, and dosing optimization policies will also need to be reconsidered. Lorlatinib will dominate the therapeutic landscape of ALK+ NSCLC until the advent of combination therapies, which are the most reasonable next step. Further strengthening of the ALK blockade with additional ALK-directed drugs could likely eliminate the small residual “leak” of late off-target resistance after the third year, while combination with emerging neoantigen-directed immunotherapies may also help control the highly aggressive and more immunogenic early-progressing tumors and facilitate cure of the disease. Universal state-of-the-art DNA/RNA NGS for newly diagnosed NSCLC is now imperative to identify and treat all ALK+ patients according to the latest therapeutic advances, which will hopefully soon also spread to earlier stages and other oncogenic drivers.

## Simple Summary

The 7-year CROWN update reaffirmed a new reality with far-reaching consequences: lorlatinib can bring metastatic ALK+ lung cancer to a standstill, with an estimated annual progression rate < 2% after the 3rd year, for most patients. This new ability to prevent acquired resistance abolishes ALK TKI sequencing and inaugurates an entire new paradigm. Clinical algorithms, principles of drug development, dosing policies, and regulatory approval processes require reconsideration. The anticipated further increase in frontline efficacy with combination regimens makes cure of ALK+ disease appear feasible for the first time. *After the Crown evolved anew*, *the rule-of-two no longer holds true*. *Three years on treatment came and went*, *the ALK affliction’s fire was spent*. *Acquired resistance*, *once the rule*, *nigh a relic of a bygone school*. *TKI follows TKI no more*, *one lasting drug we are striving for*. *The path to cure*, *long left untold*, *can now at last begin to unfold*.

## 1. Introduction

Anaplastic lymphoma kinase (ALK)-positive tumors comprise approximately 3–5% of non-small-cell lung cancer (NSCLC) and occur more frequently in younger patients who are never- or light smokers [1]. The primary driver results from fusion of the *ALK* kinase sequence with the 5′ region of a partner gene, usually echinoderm microtubule-associated protein-like(*EML*) 4, which provides the promoter and oligomerization domains to the resulting oncoprotein [2]. Since its discovery in 2007 [3], ALK+ NSCLC has been the poster child of thoracic oncology owing to several distinctive characteristics: the lowest mean tumor mutational burden (TMB), as indicator for the highest genetic stability and strongest oncogenic addiction [4]; complex management, comprising highly variable sequences of tyrosine kinase inhibitors (TKI), chemotherapy, and local ablative therapies (LAT) depending on patterns of acquired resistance and radiologic progression [5,6,7]; as well as the longest overall survival (OS) among lung cancers so far, exceeding 5 years in median already since 2020 [8]. With its exquisite TKI sensitivity, ALK+ NSCLC was also the first entity to demonstrate several key concepts of modern cancer medicine, such as the “best-first approach”, with prioritization of the second-generation (2G) drug alectinib over the 1G compound crizotinib as first-line (1L) therapy since approval of the former in 2017 [7,9]; use of next-generation sequencing (NGS) results from tissue or liquid rebiopsies at the time of disease progression to guide subsequent management [10,11]; development of the first framework for molecular risk stratification with single- and double-positive tumors based on the presence of *EML4-ALK* variant 3 (V3) and *TP53* mutations as independent risk factors validated by the prospective phase 3 ALTA-1L study [4,12]; and the potential for longitudinal, minimally invasive disease monitoring across treatment lines by combining targeted NGS of cell-free DNA (cfDNA) with profiling of other circulating tumor DNA (ctDNA) characteristics, such as copy number alterations (CNA) or epigenetic features, as well as serum proteins [13,14,15,16]. However, the best was yet to come. After all these remarkable advances, this highly dynamic field was poised for yet another, even more radical transformation catalyzed by the 3G inhibitor lorlatinib, which fundamentally changes our perception of the ALK+ disease. The aim of this review is to analyze the implications of the latest CROWN study results [17], which redefine the treatment paradigm and future roadmap for ALK+ and other oncogene-driven cancers. Pertinent material was drawn from publications identified in Pubmed and presentations in major congresses (ESMO, ASCO, WCLC, ELCC, AACR) during the last 20 years (2007–2026).

## 2. From Exponential Improvements to Disease Inactivation

### 2.1. The Rule-of-Two for ALK TKI Efficacy and How Lorlatinib Transcends It with Longer Follow-Up

The unparalleled improvement in outcomes during the last decade, from a median progression-free survival (mPFS) of approximately 7.3 months under pemetrexed to several years currently [17,18,19,20,21,22], was facilitated by an exponential increase of efficacy across three consecutive TKI generations—a unique achievement unlikely to be observed again in any other medical setting. As shown by randomized head-to-head comparisons, the 1G ALK inhibitor crizotinib is twice as good as chemotherapy based on a hazard ratio (HR) for the PFS of approximately 0.5; the 2G TKI alectinib and brigatinib are twice as good as crizotinib, again based on a PFS HR of approximately 0.5; and the 3G compound lorlatinib initially appeared to be also twice as good as 2G drugs based on a PFS HR vs. crizotinib which was approximately half that of 2G TKI vs. crizotinib in the respective phase 3 studies (Table 1). Considering the extraordinary activity of 2G ALK TKI, with a median 1L PFS of 2.5–3 years according to investigator assessment, already almost twice as long as that with 1L osimertinib in FLAURA (18.9 months [23]), the further exponential efficacy improvement reported in the interim CROWN analysis of 2020 and confirmed by the 3-year update in 2022 already bordered on impossible [21,24]. However, with longer follow-up, something much more intriguing would be revealed.

The PFS drop rate, which was approximately 10% during each of the first two years in CROWN (excluding the erroneously enrolled 14/149 patients with false-positive ALK immunohistochemistry and disease progression [PD] within the first year, reported as abstract [25]), gradually decreased and stabilized at a very low average level of 2.5% annually (range 2–3%) after the third year (Table 2, third column) [17,26]: 2–4 PFS events among 66–81 patients at risk in years 4–7, according to the published raw data. Some of these PFS events represent deaths due to unrelated causes; for example, among seven PFS events between the CROWN five-year and seven-year analyses with a PFS decrement of approximately 5%, only four events were due to disease progression, while the remaining 3/7 represented unrelated deaths during remission [17]. Therefore, the true rate of PD is even lower, <2% on average (range 1–2%), after subtracting the age-adjusted mortality of the general population comprising approximately 1.0%, as explained in the Supplementary Materials (Table 2).

Therefore, lorlatinib is not just >2x stronger than earlier drugs, but has additionally exceeded a critical threshold: it is so effective that it can almost stop further development of the disease for several years in the majority of patients, as evidenced by the PFS curve plateauing at a very high level exceeding 50% [17]. Similar plateaus were observed under lorlatinib for all CROWN patient subsets analyzed so far, i.e., for patients with and without brain metastases (BM), patients with tumors harboring *EML4-ALK* variant 3 (V3) or V1, *TP53* mutated or wild-type status. In published subgroup analyses, the exact level of the plateau would vary according to the patient’s risk profile, but a very long PFS with curve flattening at a high level was always present, indicating a realistic chance of long-term disease control with a very low risk of late progression for patients with any baseline characteristics (partly reported in abstract form) [26,27].

**Table 2:** PFS rate, PFS drop rate, and estimated progression rate under lorlatinib in CROWN.

CROWN Follow-Up	PFS Rate Original (Adjusted) ^1,2^	PFS Drop Rate/yr Original (Adjusted) ^1,2^	Conditional PFS Drop Rate/yr ^1^	Numbers at Risk (PFS Events) ^3^	Progression Rate/yr Estimated (Adjusted) ^1^
year 1	80% (88%)	20% (11.7%)	18.8%	149 (28)	n/a
year 2	70% (77%)	10% (11.0%)	12.6%	111 (14)	n/a
year 3	65% (72%)	5% (5.6%)	7.5%	93 (7)	n/a
year 4	63% (70%)	2% (2.2%)	2.5%	81 (2)	1% (1.2%)
year 5	60% (66%)	3% (3.3%)	5.1%	78 (4)	2% (2.3%)
year 6	57% (63%)	3% (3.3%)	4.2%	71 (3)	2% (2.3%)
year 7	55% (61%)	2% (2.2%)	3.0%	66 (2)	1% (1.2%)

^1^ The annual “PFS drop rate” (third column) indicates the percentage-point decrement of the Kaplan–Meier estimates of PFS rates (second column) published in the original study results (Figure 1A of [17]). The annual “conditional PFS drop rate” (fourth column) has been approximated by dividing the number of PFS events by the number of patients at risk at interval start (both provided in the fifth column) and is conservative under heavy censoring for year 7. The (adjusted) annual “progression rates” could be approximated only for the plateau phase after the third year from the (adjusted) annual “PFS drop rates”, after subtracting the annual age-specific mortality of the general population comprising approximately 1.0%, as explained in the Supplements [28]. ^2^ “Adjusted” PFS rates and estimated “adjusted” PFS drop rates were calculated after subtracting the 14 patients enrolled with false-positive ALK results and early progression within the first year (reported as abstract [25]). ^3^ Numbers at risk at the beginning of each year and PFS events during each year (calculated by subtracting the reported number of censored cases from the reported number of patients at risk) are original data from the CROWN study results, according to Figure 1A of [17]. PFS rates are rounded without decimals. For PFS drop rates (and the derivative conditional and progression rates), original values from CROWN are available only as rounded numbers without decimals, while values calculated by the authors are given with one decimal.

### 2.2. The PFS Plateau as Unique and Pivotal Property of Lorlatinib

Thus, the focus is gradually shifting: the distinguishing characteristic of lorlatinib is not just “a longer” or “the longest” PFS compared with that of ALK inhibitors any more, but rather its unique ability to bring a metastatic cancer to a standstill. Five-year survival plateaus in advanced NSCLC had thus far been possible only with (chemo-)immunotherapy, and only for small patient subsets comprising up to 20–30% [29]. In contrast, the PFS plateau under lorlatinib in CROWN is exceptional in two respects: not only the first ever observed with a targeted drug, but also much higher, encompassing the majority of patients. Of course, there is also another important difference: while PD-(L)1 inhibitors can be stopped after the second year and adequate workup without compromising survival [30], the continued benefit under lorlatinib requires ongoing administration of the drug. Notwithstanding, this accomplishment remains unequaled across the entire field of medical oncology for several reasons, which can be better appreciated after some conceptual clarifications.

The term “plateau” is not new, but it has occasionally been used in the past when referring to upfront administration of earlier ALK TKI, e.g., brigatinib in the seminal NEJM publication of the ALTA-1L study [20], as well as alectinib, ceritinib and even crizotinib in several secondary analyses and reviews [7,31,32]. However, the PFS estimates available at that time did not exceed 50% at 3 years and were subject to substantial censoring, as the PFS follow-up in ALEX, J-ALEX, ALTA-1L, and ASCEND-4 was of similar duration and not subsequently extended [8,12,33,34]. Within the old paradigm, the word “plateau” was only a descriptive expression for the shape of the PFS curve and served only to suggest that 2G TKI should be preferred upfront over crizotinib because they offered a “long-lasting benefit over 2 years”—a duration that seems short by current CROWN standards but was substantial at the time, as both 1L crizotinib and 2L 2G TKI showed a mPFS of only about 1 year [7]. In contrast, the word “plateau” assumes a very different and specific meaning in the new paradigm emerging from the last CROWN update: it denotes a true plateau, which is verified, i.e., without censoring, and characterized by a consistent and confirmed very low annual PFS decline of only 2.5% after year 3, because we can observe it with a much longer follow-up of 7 years [17]. Such a semantic shift of established terms is a normal occurrence when scientific paradigms change [35].

**Figure 1:**
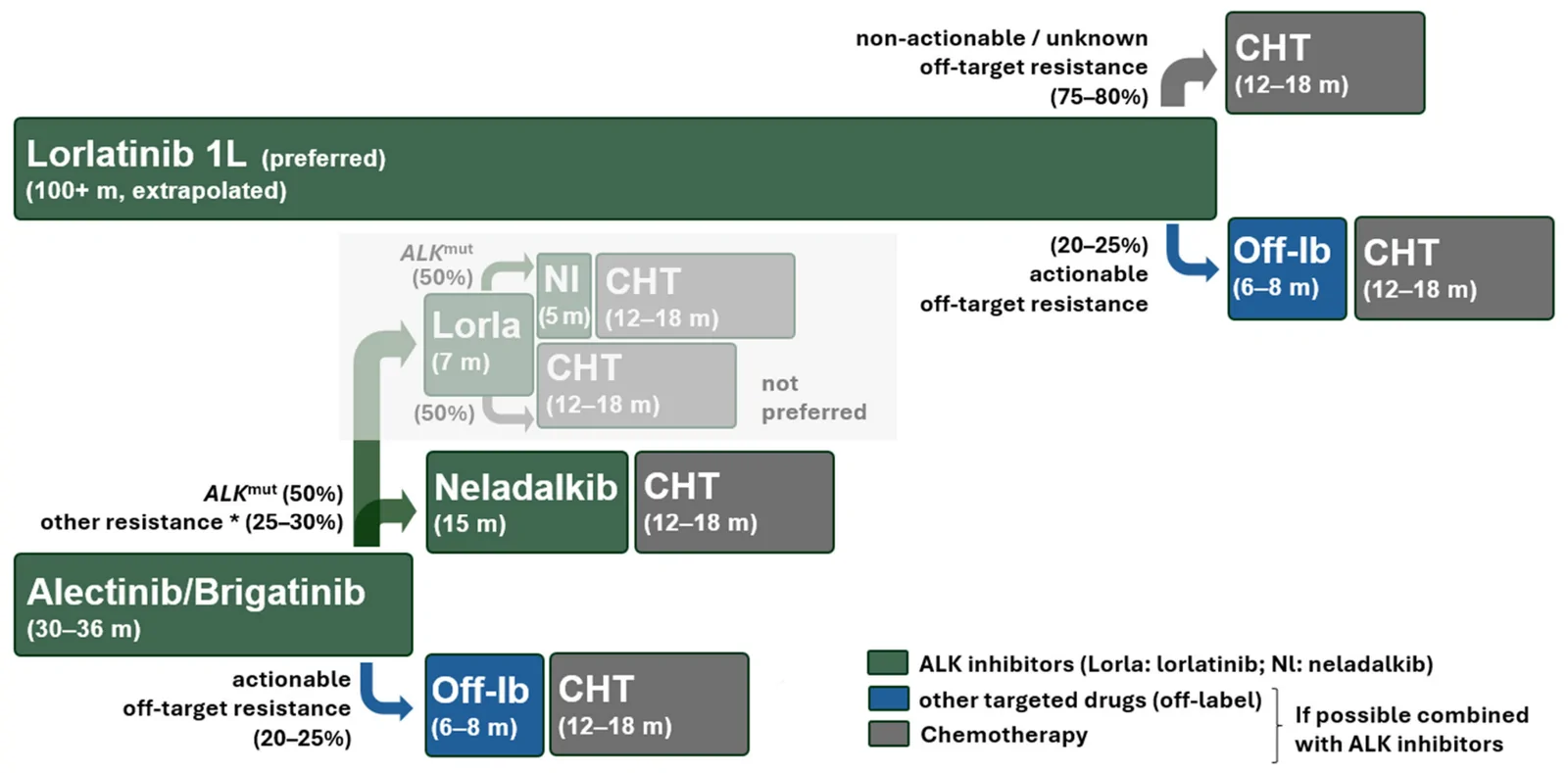
Current management algorithm for ALK+ NSCLC after CROWN. For each first and subsequent line option, the length of the bar is proportional to the respective median PFS (rounded in parentheses), as assessed by the investigators according to published data. (NB the 100+ months of PFS for lorlatinib are an extrapolation). Further details are given in the main text (Section 2.4, Section 3.1, Section 3.4 and Section 4.2). * shorter survival.

With a 7-year PFS of 55% and a consistent annual decrement of 2.5% after year 3 (Table 2), the median 1L PFS under lorlatinib is projected to be approximately nine years (>100 months), which no other drug has ever achieved for metastatic cancer (Figure 1). Moreover, lorlatinib is the first ever drug to demonstrate de facto superiority of still formally non-evaluable OS results over earlier TKI: its 55% PFS rate at 7 years in CROWN is already substantially longer than the 7-year OS rates of alectinib (48.6%) and crizotinib (38.2%) in ALEX, although mature OS results have not yet been reported from CROWN, because the 139 deaths (70% information fraction) required for the protocol-specified second interim analysis have not yet occurred [17,36]. While cross-trial comparisons have limitations, the PFS in CROWN and PFS/OS in ALEX are mathematically very similar time-to-event endpoints, calculated using very similar methods and similar stringency within the context of registrational trials, in very similar patient populations: contemporary treatment-naïve ALK+ patients with ECOG performance status of 0–1, brain monitoring, availability of stereotactic radiotherapy, etc. This de facto superiority of lorlatinib renders some earlier network metanalyses that calculated similar or longer survival after 2G drugs, such as [37,38], obsolete, because this cannot be plausibly reconciled with the projected mPFS of >100 months in CROWN vs. the reported mOS of 81 months in ALEX. Moreover, the enormous difference in the 1L PFS of lorlatinib vs. 2G drugs is further underlined by the updated results in the control arm of CROWN: the PFS rate for crizotinib after 7 years dropped to 3%, which clearly shows that 1L crizotinib cannot produce a survival plateau [17]. Considered together with the fact that (i) 2G TKI after crizotinib do not produce a plateau either but show PFS similar to that of 1L crizotinib [7]; and (ii) 2G TKI in the 1L have shown OS identical to that of 1L crizotinib in phase 3 studies with unrestricted access to 2G TKI post-crizotinib (i.e., J-ALEX for alectinib and ALTA-1L for brigatinib) [12,39]), this leads to the conclusion that 2G ALK TKI cannot produce a true PFS plateau even if given upfront, either. For 1L alectinib, the lack of a PFS plateau in the 1L has also been empirically confirmed by ALESIA, which reveals a continuous PFS drop until the end of its uniquely long follow-up at >60 months [40]. 2G ALK TKI given upfront do result in a 5-year PFS rate of approximately 35% based on ALESIA and ALEX (with 34.9% of patients still on alectinib at the 5-year update [8]), but cannot achieve a true PFS plateau, which is defined by the slope of the PFS curve and a distinct property. ALESIA and ALEX also provide important insights into how prohibiting crossover impacted the outcomes of 2G TKI studies. First, it reduced exposure to subsequent ALK TKI post-crizotinib by approximately 1/3 (54% in ALEX and 58% in ALESIA banning crossover, vs. 83% in J-ALEX and 82% in ALTA-1L permitting crossover), which corresponds to an artificial undertreatment of the control arm by today’s standards, as 2G TKI are freely available and recommended for all patients failing crizotinib in the routine setting. Second, it was associated with a transient separation of the OS curves favoring alectinib over crizotinib, followed by reconvergence at the 7-year updates of both ALEX and ALESIA, with no significant benefit for the 2G arm (partly reported as abstract) [36,41]. In contrast, the OS curves for the 2G TKI and crizotinib in J-ALEX and ALTA-1L (which allowed crossover) remained indistinguishable throughout the entire follow-up [12,39]. The OS patterns observed in ALEX and ALESIA are consistent with shorter survival among some patients with PD on crizotinib and poorer prognosis (potentially compounded by limited access to 2G TKI), but patients with a more favorable prognosis appear to have similar long-term survival whether they start with alectinib or crizotinib. If 2G TKI were able to cause a true PFS plateau, we should instead observe an increasing separation over time between the OS curves for, e.g., alectinib and crizotinib (which does not entail a plateau as explained above and based on the 7-year CROWN results) in all trials, regardless of whether or not crossover was permitted. Hence, the 7-year CROWN update, including the latest results from both arms, becomes the touchstone that reaffirms the capability of a PFS plateau for lorlatinib, while disproving it for all previous ALK TKI at the same time.

### 2.3. “Inactive Disease” as a Novel Goal of Treatment and Objective for Clinical Trials

The outcomes achieved with lorlatinib are so favorable that even the recently coined “transformation of ALK+ NSCLC into a chronic disease” may be an understatement. “Chronic disease” implies survival with recurrent or persistent symptoms, side effects and limitations, as exemplified by some patients with less aggressive hormone-sensitive metastatic prostate or breast cancer [42,43]. In contrast, the 5- and 7-year CROWN updates provide a strikingly different picture: among the 65% of patients who remained progression-free at the end of year 3, no additional patient developed BM after month 30 (0/89), no further treatment-related discontinuations of lorlatinib occurred after month 26 (0/89), and no new patient developed grade 3–4 adverse events (AE) between the 5- and 7-year analysis, although the denominator for this latter observation is not reported [17]. In fact, the exceedingly low estimated annual risk of progression < 2% after year 3 in CROWN appears to be numerically lower than that of some premalignant or precursor conditions, such as leukoplakia (crude incidence ca. 5% per year or 39/170 patients after a median follow-up of 54 months [44]), or smoldering multiple myeloma (crude incidence ca. 5% per year, or 108/308 patients after a median follow-up of 79 months [45]). Although the estimated average annual incidence of PFS events in CROWN across the entire seven years is comparable to that of the aforementioned precursor conditions (crude PFS rate ca. 5.8% per year or 60/149 patients after a median of 83 months [17], which can be adjusted to approximately 4.8% per year by the exclusion of unrelated deaths, as described in Section 2.1), the ratio of late/early progression is lower for lorlatinib (ca. 17%) compared to leukoplakia (ca. 100%) or smoldering myeloma (ca. 50%, Supplementary Material). Therefore, “inactive disease” would arguably be a more appropriate term than “chronic disease” for patients with ALK+ NSCLC completing 3 years under lorlatinib. Drug-induced disease inactivation operates in parallel with patient selection, as only approximately 2/3 of lorlatinib-treated patients could enter the fourth year in CROWN, but remains a unique feature of lorlatinib, as it has not been observed with any earlier ALK TKI (Section 2.2).

At the same time, the annual rates of disease progression (PDR) and PFS drop (PFSDR) during this late treatment phase emerge as important novel endpoints against which the efficacy of every future ALK-directed drug will inevitably be measured (Table 2). Based on the data currently available from CROWN, and for the sake of simplicity, in this study we defined PFSDR as the annual decrement of the PFS curve, and approximated the PDR and the conditional PFSDR based on the scant data available from the 7-year analysis (Table 2 and Supplementary Material) [17]. If exact information about the type of PFS events (PD vs. related/unrelated deaths) becomes available from CROWN and other similar studies in the future, the PDR and conditional PFSDR could be calculated exactly and used as complementary benchmarks (Table 2). These endpoints reflect the slope rather than the height of the PFS curve and are presumably more important for long-term outcomes than landmark PFS rates (e.g., 60% for lorlatinib at 5 years) and the mPFS, because they quantify the ability of a drug to inactivate (“turn off”) malignant disease at a specific timepoint, which is the underlying biologic process, rather than the likelihood of patient survival over a longer time period, which is the phenotypic result. Furthermore, robust estimates for these “slope”-endpoints from clinical trials will likely be available earlier than the mPFS and be more robust against methodological shortcomings, like the erroneous inclusion of some false-positive patients in CROWN (Table 2). The strong and durable efficacy of lorlatinib, together with the rigorous conduct of the CROWN trial, including regular computed tomography (CT) and magnetic resonance imaging (MRI) assessments over many years, enabled, for the first time, an accurate evaluation of the PFSDR, whereas this would not have been feasible or reliable with shorter follow-up or the fewer radiologic assessments typically performed in other clinical trials.

As the PFSDR and PDR are new potential endpoints, no surrogate markers exist yet. The systemic (ORR) and intracranial overall response rates (icORRs) are not useful, because they are very similar for all ALK inhibitors (Table 1). Among radiologic measures, the achievement of intracranial complete response (icCR) appears to be most informative, as it was much higher for lorlatinib (>70%) compared to earlier ALK TKI (0–38%). In biological terms, the achievement of CR may indicate a drug’s ability to eradicate resistance clones, which may ultimately protect from further progression and facilitate the very low PFSDR and PDR observed in CROWN. The same notion was recently reinforced by the results of the phase 2 LORIN trial, in which 3 months of neoadjuvant lorlatinib could achieve an unprecedented pathologic complete response (pCR) rate of 47%, much higher than the 12% observed earlier with alectinib (reported as abstract) [46,47]. It remains unclear, however, why the systemic CR rate of lorlatinib in CROWN was only <5% and similar to that of other drugs (Table 1). Presumably, the primary lung tumor and other large lesions may persist as residuals on CT despite the absence of vital tumor tissue, whereas repeat assessment of the generally smaller intracranial lesions with the more sensitive brain MRI can capture tumor eradication better. Regardless, fine granular radiologic assessment has demonstrated a significant correlation between the depth of response and longer PFS both under lorlatinib and brigatinib (reported in abstract form) [48,49]. Furthermore, the drop in plasma ctDNA levels 4 weeks after initiation of lorlatinib correlates with deeper radiologic responses and longer PFS, but it remains unclear whether these early ctDNA dynamics could identify patients poised to subsequently enter the PFS plateau (i.e., achieve PFS > 3 years under the drug) [50,51]. Functional imaging with PET/CT, radiomic studies with novel machine-learning algorithms, and ctDNA monitoring using highly sensitive minimal-residual disease (MRD) assays may provide early predictors and surrogates for the achievement of a PFS plateau in the future [52,53,54].

### 2.4. Implications for Patient Survival and the Clinical Algorithm

This unique ability of lorlatinib to achieve a PFS plateau is catalytic for clinical decision-making: patients in the plateau phase enter an alternate reality, in which the clock of the disease has practically stopped. The perspective of this “time dilation” becomes the single most important reason to prioritize lorlatinib over other ALK inhibitors upfront, because the window of opportunity exists only in the first line. With a projected mPFS of 9 years in the first vs. 7–8 months in subsequent lines, lorlatinib is a different drug in the treatment-naïve setting and acquires priority due to this property, regardless of other details, such as the slightly different rates of second-line treatment in phase 3 trials of earlier ALK inhibitors. Moreover, the superiority of lorlatinib will increase further with longer follow-up, because patients starting with 1G/2G drugs do not enter a plateau phase, but keep progressing at higher rates (Section 2.2). This survival advantage will be further enhanced by novel drugs, such as antibody–drug conjugates (ADCs), targeted protein degraders (TPDs) or next-generation immunotherapies, which may become available in the coming years, as more lorlatinib-treated patients may live longer to receive them compared to patients starting with 1G/2G drugs today. Consequently, an ever-growing number of physicians and some guidelines, like the German S3 (highest-level) national lung cancer guideline, but not, notably, the ESMO or NCCN guidelines yet, recognize the vital importance and explicitly prioritize lorlatinib as the preferred 1L treatment for metastatic ALK+ NSCLC [5,55,56]. In any case, 2G ALK TKI as first-line treatment remain important, e.g., for patients with contraindications to lorlatinib, like serious preexisting neurologic or psychiatric illness, or intractable toxicity [55]. However, less obvious than the clinical consequences are the translational implications of the latest CROWN results, which are crucial for future developments in the field.

## 3. Acquired Resistance Becomes (Almost) Preventable

### 3.1. ALK-Dependent Resistance Is Completely Abrogated with Frontline Lorlatinib

Understanding how acquired resistance emerges under ALK TKI is essential for unravelling the biological underpinnings of the lorlatinib plateau. Molecular profiling of patients progressing under lorlatinib in CROWN has not yet revealed any *ALK* resistance mutation, whereas on-target resistance is encountered in approximately 50% of cases failing either 2G ALK TKI (mostly single mutations) or lorlatinib in later lines (mostly compound mutations) [57,58]. Acknowledging the relatively small number of patients progressing under lorlatinib with available molecular results (*n* = 31 so far, [17,26]) and that the ctDNA assays used for profiling may occasionally miss alterations due to limited shedding, this paucity is still remarkable and suggests complete suppression of ALK-dependent (on-target) resistance when lorlatinib is used upfront. The absence of compound *ALK* mutations in this setting was expected and had already been predicted based on preclinical data several years ago, as their development requires preexisting single *ALK* mutations to have emerged during previous treatment with 1G/2G inhibitors [59]: for example, when *ALK* p.G1202R is already present at the time of lorlatinib initiation, lorlatinib cannot prevent the emergence of a second, and thus compound, *ALK* mutation, with high-level resistance to the drug. However, at the same time, lorlatinib could suppress the emergence of any *ALK* mutation when given upfront in CROWN, including *ALK* p.L1256F, which is highly resistant (IC50 > 1000 nM [60]), and *ALK* p.G1202R with an IC50 for lorlatinib 5–50x higher (e.g., 50 nM or 487 nM [58,61]) than other *ALK* mutations (<10 nM [58]). Why?

### 3.2. ALK Activity Drives the Development of On-Target Resistance

These observations are reconciled if we consider that emergence of *ALK* resistance mutations is driven by the activity of the ALK oncoprotein itself, i.e., requires sufficient residual ALK signaling. This is evident by the patients’ phenotype: *ALK* resistance mutations, in particular *ALK* p.G1202R, are much more frequent in patients with tumors harboring the more active *EML4-ALK* V3 (E6:A20), which is more stable and triggers stronger downstream signaling than other *ALK* fusion variants [62,63,64,65]. The longer half-life and stronger activity of the shorter *EML4-ALK* V3 compared to V1 are explained biochemically by the absence of truncated β-propeller domains, which increase propensity to degradation and reduce interaction with the cytoskeleton [66,67]. However, emergence of *ALK* p.G1202R partially impairs the ALK kinase and creates a disadvantage for ALK-addicted tumor cells, as the mutation alters the kinase domain structure around the active site, which has been optimized by evolution over > 700 million years [68]: this is evident by the decrease in ALK phosphorylation when, for example, *ALK* p.G1202R or *ALK* p.L1196M are introduced into BaF3 cells compared to the ALK phosphorylation in ALK WT cells [59]. Consequently, emergence of *ALK* p.G1202R and other *ALK* resistance mutations under treatment with 2G TKI is better tolerated by the tumor cells against a background of higher baseline ALK phosphorylation as provided by *EML4-ALK* V3 compared to V1 [62]. Lorlatinib, however, suppresses the activity of ALK, including V3 [11], very deeply, and can apparently block this.

The prevention of *ALK* resistance mutations resulting from the deeper suppression of WT ALK works differently than the treatment of established *ALK* mutations, which absolutely depends on the respective IC50 against the particular mutation. For example, although lorlatinib cannot treat *ALK* resistance mutations as well as neladalkib, which is evident by a shorter mPFS of 6.6 vs. 14.5 months after 2G ALK TKI, respectively [69,70], lorlatinib can nevertheless completely block emergence of *ALK* p.G1202R and other *ALK* resistance mutations, as its IC50 for the WT ALK is excellent and similar to that of neladalkib (≈1 nM for both [61]). This dissociation between the preventive and therapeutic capacity of a novel targeted drug with respect to acquired resistance is an additional important indirect conclusion from the 7-year CROWN update with crucial importance for further drug development, as will later be discussed in Section 4.2. In clinical terms, lorlatinib, as a stronger ALK inhibitor, has consistently shown much better outcomes for *EML4-ALK* V3-driven tumors than 2G drugs, with a mPFS of 60 months compared to only 16–18 months achieved by brigatinib and alectinib [12,17,71]. However, even 60 months is substantially shorter than the projected 100+ months for the mPFS of the overall population in CROWN (Section 2.2). If lorlatinib can fully eliminate ALK-dependent resistance, why does it not completely abrogate the *EML4-ALK* V3-associated phenotype?

### 3.3. ALK Activity Also Facilitates the Development of ALK-Independent Resistance

**Figure 2:**
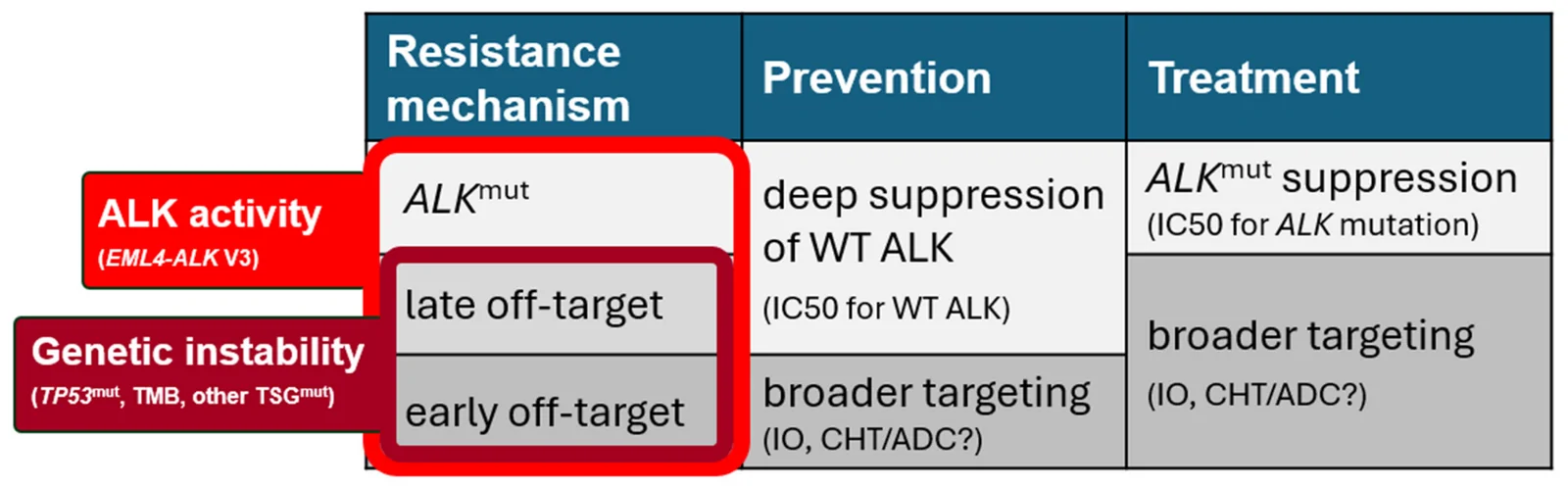
Activity of the ALK oncoprotein and genetic instability (with molecular surrogates in parentheses) as the two key drivers of on-target (*ALK*^mut^) and off-target acquired resistance in ALK+ NSCLC, along with potential prevention and treatment strategies. The benefit from chemotherapy-based approaches has so far been temporary and questionable in the long run. Further details are given in the main text (Section 3.1, Section 3.2, Section 3.3, Section 3.4, Section 5.1 and Section 5.2).

The reason is that stronger ALK signaling and *EML4-ALK* V3 also drive the development of off-target resistance. This is again evident from the patients’ phenotype. *TP53* mutations, which destabilize the genome and facilitate emergence of complex resistance mechanisms, such as *MET*^amp^ and other CNAs, can exert their full phenotype only against a background of stronger ALK phosphorylation, as provided by *EML4-ALK* V3: double-positive V3 + *TP53*^mut^ patients have the most extensive metastatic spread at initial diagnosis, the highest number of CNAs in ctDNA, and the shortest survival compared to all other ALK+ NSCLC patient subsets [13,72]. At the biological level, stronger oncogenic signaling activates anti-apoptotic and stress-adaptation pathways and can help tumor cells survive the potentially detrimental, complex genetic events that are necessary for the development of ALK-independent resistance [73]. Indeed, lorlatinib, as a stronger ALK TKI, could produce a much longer mPFS of 51.6 months for ALK+ patients with *TP53*^mut^ compared to only 18–21 months under alectinib and brigatinib [12,17,74]. A similar ability of stronger oncogene suppression to attenuate both on-target and off-target resistance has been observed in the MARIPOSA trial, in which complex resistance mechanisms were also less frequent after the addition of the EGFR/MET-bispecific antibody amivantamab to the 3G EGFR inhibitor lazertinib vs. the 3G EGFR inhibitor osimertinib alone; the addition of chemotherapy to osimertinib, on the other hand, did not alter the resistance pattern compared to osimertinib monotherapy in the FLAURA2 trial (reported in abstract form) [75,76]. This exquisite activity of lorlatinib against *TP53*-mutated tumors compared to 2G drugs is not due to any p53 inhibitory effects, but rather to the deeper suppression of WT ALK, whose activity is essential for the pathogenesis of ALK-independent resistance and the *TP53*^mut^ associated phenotype (Figure 2).

### 3.4. The Suppression of Off-Target Resistance by Lorlatinib Is Incomplete

Consequently, off-target resistance is also hampered and delayed under frontline lorlatinib, which contributes to the success of the CROWN: if off-target resistance were not affected by lorlatinib, it would have simply replaced the canceled on-target resistance with ultimately little or no survival benefit for the patients. However, in contrast to the on-target resistance, which is fully eliminated, the suppression of ALK-independent resistance under 1L lorlatinib is incomplete, as alterations in *MET*, *MAP2K1*, *KRAS*, *NTRK1*, *MYC*, *RB1*, as well as other off-target mechanisms have been identified in CROWN patients at progression [17]. In clinical terms, the incomplete suppression of ALK-independent resistance explains both the small leak of disease progression with a constant rate < 2% annually after the third year in CROWN (“late off-target resistance”, column 6 of Table 2) and the higher annual progression rate of 5–10% during years 1–3 (“early off-target resistance”, roughly estimated from the PFS decline in Table 2, column 3 [adjusted values], after subtracting the baseline mortality rate of 1.0%, as described in Section 2.1). Both late and early off-target resistance are promoted by the activity of ALK (Figure 2), as the rates of both late and early progression were lower in CROWN (Table 2) compared with, e.g., ALEX: the latter did not show a PFS plateau and had a higher PFS drop of 43% in the first two years [8], a substantial proportion of which (approximately 50% longitudinally [58]) is caused by off-target resistance. One practical consequence is that rebiopsies, at the time of disease progression under lorlatinib, remain imperative to identify off-target resistance mechanisms and characterize their actionability before choosing the subsequent therapy. According to the 7-year CROWN update, these may be potentially treatable using off-label or experimental drugs—in combination with ALK TKI, if possible [77]—in approximately 20–25% of cases, which is similar to the frequency of actionable off-target resistance encountered after 2G ALK TKI (Figure 1) [17,78]. While published data are scarce, the expected mPFS from the off-label use of targeted drugs to treat off-target resistance in ALK+ NSCLC can be estimated at 6–8 months based on the few published cases, anecdotal reports and the larger experience in EGFR+ disease [79,80] (Figure 1). The incomplete suppression of off-target resistance in ALK+ NSCLC after the third year under lorlatinib also suggests that the disease is not fully neutralized yet and that even stronger upfront therapies may be necessary before considering the patients as cured and eligible to stop pharmacotherapy without significant risk of relapse.

## 4. The End of ALK TKI Sequencing

### 4.1. ALK-Directed Therapies Beyond the First Line Become Meaningless

Our new ability to prevent acquired resistance will inevitably result in abolishment of a key therapeutic principle: the end of ALK TKI sequencing, which has been a cornerstone of management within the previous paradigm and contributed substantially to survival improvements over the last decade [6,7]. If lorlatinib is so active that it must be positioned upfront (Section 2.4), and no ALK resistance mutation can arise after lorlatinib (Section 3.1), then there is no room for a subsequent ALK inhibitor in the treatment trajectory. An exception is, of course, re-exposure with the same or another ALK TKI in later lines after, e.g., intervening chemotherapy, which can still be helpful in selected cases, as resensitization can occur due to clonal tiding [81]. This “winner-takes-all” emerging new paradigm of ALK+ disease (Table 3) is excellent news for patients entering the plateau, but also harsh for the others, as well as for several novel ALK-directed drugs, which remain in development based on the premises of the old paradigm. For example, several arms with pretreated patients of a clinical trial testing the ALK TPD TRI-611 since March 2026 (NCT07491497) have now limited value (reported as abstract) [82], while another trial of prophylactic vaccination using neopeptides derived from ALK resistance mutations (NCT05950139) loses most of its practical relevance despite successful results (reported as abstract) [83]. However, by far the most important implications concern the 4G ALK inhibitor neladalkib.

### 4.2. The Benefits but Precarious Position of Neladalkib and Other “Soon-After-First” Novel Drugs

Neladalkib was carefully designed to combine all favorable efficacy and tolerability features [61], but its development began in the transitional, early post-CROWN period, during which the old paradigm was gradually fading away, while the new one had not yet fully formed (Table 3). Consequently, several key strategic decisions were driven primarily by the goal of retaining activity after lorlatinib, which has now been revealed as largely futile. According to the flagship ALKOVE-1 cohort, the results of which were presented as abstract at ASCO 2026, the ORR of 26% and mPFS of 4.6 months for neladalkib in lorlatinib-pretreated patients are comparable to those of platinum-based (immuno-)chemotherapy [69,84]. As neladalkib after lorlatinib works better for patients with detectable *ALK* resistance mutation [85], neladalkib could be prioritized after lorlatinib in case of acquired on-target resistance, e.g., *ALK* p.G1202R and other (compound) mutations. However, for the remaining majority, chemotherapy will generally be the safer and more effective option (Figure 1) [57]. For neladalkib directly after 1L lorlatinib, although its activity has not been separately reported yet, efficacy is probably even lower and of little practical relevance [69]. One particular risk with next-line therapies that do not work is early attritional death due to rapid clinical deterioration, which precludes administration of other available options [86]. These include, at least, chemotherapy, which offers an OS of 12–18 months after next-generation ALK TKI, particularly if combined with continued administration of ALK inhibitors (Figure 1) [87].

On the other hand, the activity of neladalkib in lorlatinib-naive pretreated patients compares favorably with that of lorlatinib (mPFS 14.5 vs. 6.6 mo after 2G TKI, respectively) [69,70]. This is explained by its lower IC50 for *ALK* p.G1202R and other *ALK* resistance mutations (Section 3.2). Therefore, neladalkib becomes the preferred option after, e.g., alectinib or brigatinib, but the survival benefit compared to the sequence of lorlatinib followed by neladalkib will be only 2–3 months in a palliative situation, which will near extinction as the 1L use of lorlatinib increases further (Figure 1). In the new era of preventable resistance, use in the 1L becomes the only viable option for any novel TKI, but also extremely difficult at the same time.

A randomized phase 3 1L trial of neladalkib to demonstrate superiority over lorlatinb could continue for a very long time with an uncertain outcome, as the IC50 against WT ALK is similar for both drugs (Section 3.2), while a non-inferiority design would require many more of these rare patients. Neladalkib is currently circumventing this impasse by a randomized comparison with alectinib in the ALKAZAR trial [88], but this entails other significant risks. As lorlatinib de facto improves OS compared to alectinib (Section 2.2) and is available in most countries participating in ALKAZAR [88], randomization is becoming increasingly difficult. Furthermore, there is a risk of preferentially attracting less fit, lorlatinib-ineligible patients, who may skew study outcomes and create additional problems: for wide adoption, neladalkib will be required to show at least equipoise vs. lorlatinib, i.e., a similar very low long-term PFSDR < 3%, as confirmed by the 7-year CROWN update. However, this may be impossible if many ALKAZAR patients are lorlatinib-ineligible, i.e., less fit, with an inherently higher propensity to develop events.

A further problem stems from the chosen recommended phase 2 dose (RP2D) for neladalkib, which results in a 12x lower steady-state maximum plasma-free concentration compared to lorlatinib (40 nM with 150 mg daily vs. 483 nM with 100 mg daily, respectively) [61]. With once-daily dosing for both drugs, the trough and intracranial free drug concentrations for lorlatinib are also expected to be considerably higher (approximately 8–9x and 6x, respectively, as reported in the Supplementary Materials), despite the somewhat longer half-life (approximately 40–55 h, reported as abstract [89]) and higher unbound brain-to-plasma concentration ratio (K_p,uu,brain_ approximately 0.16 [61]) of neladalkib compared to lorlatinib (approximately 24 h [90] and 0.11 [91], respectively). This was a deliberate choice for neladalkib, whose RP2D was not governed by safety limitations, as was the case with lorlatinib [90], but calculated lower within the tolerable range aiming for a better balance between tolerability and sufficient control of *ALK* resistance mutations [61]. Indeed, the 12x lower steady-state maximum concentration of neladalkib is also associated with an estimated 10x lower TRKB inhibition compared to lorlatinib (3% vs. 31%, respectively) [61], which explains its lower rate of neurologic AE. At the same time, however, this raises the concern that the inhibitory effect of neladalkib on WT ALK may also be less potent, as the IC50 of both drugs is similar (ca. 1 nM [61]). From another perspective, the better ALK/TRK(B) selectivity of neladalkib vs. lorlatinib holds true only for *ALK* resistance mutations, but not for WT ALK [61]. As a deeper suppression of WT ALK appears to play a key role in the prevention of acquired resistance (Section 3.2), the 6–12x lower plasma-free drug concentration of neladalkib may represent a disadvantage, which could be further amplified whenever dose reductions become necessary, e.g., due to hepatotoxicity [69], in contrast to lorlatinib, whose relative overdosing allows dose adjustments for AEs without jeopardizing disease control [17]. Empirical, definitive answers for these issues will be provided by the ALKAZAR trial.

Neladalkib, being developed “soon after the first” drug (lorlatinib) to inaugurate the new paradigm for ALK+ NSCLC, exemplifies the major challenges of this transitional era (Table 3), but is not alone. Other excellent drugs may also face similar problems as the new paradigm spreads to further oncogenic drivers in the future. For example, if 1L taletrectinib becomes the new standard of care for ROS1+ NSCLC and, as highly potent against p.G2032R [92], completely abrogates on-target resistance, the recently approved next-generation inhibitor zidesamtinib may see very limited use for pretreated patients despite the impressive ORR of 43% after taletrectinib in later lines in the ARROS-1 study (reported as abstract) [93]. Frontline use will then become the only viable option and available drugs will be prioritized mainly based on their 1L PFS—but it will not be helpful here, either, that the RP2D for zidesamtinib was selected at the lower end of the tolerability range, with the aim of achieving a better balance between safety and treatment of acquired resistance, while its IC50 for WT ROS1 is very close to that of taletrectinib [94].

### 4.3. Pitfalls and Unmet Needs in Drug Development and Regulatory Requirements

Drug development will be catalytically impacted by the new paradigm (Table 3). One key issue that may warrant reconsideration is the dosing strategy. Since its inception in 2021 [95], the United States Food and Drug Administration (FDA) Optimus program has reduced the dose of several new drugs, e.g., for the EGFR exon 20 inhibitor sunvozertinib from 300 mg in the phase 2 and early 2L Chinese NMPA label, to 200 mg/d in the later 2L FDA label and the global 1L WU-KONG28 study [96,97]. While the aim is to improve quality of life for the large number of patients receiving conventional TKI with palliative intent, this policy is not compatible with the new paradigm, which calls for exploiting the full potential of exceptional new drugs and striving for a PFS plateau and long-term disease inactivation whenever possible (Table 3). Prevention of acquired resistance improves with deeper suppression of the primary oncogene using higher TKI doses or drug combinations, as discussed in Section 3 and demonstrated by several preclinical and clinical studies [75,95,98,99,100]. Therefore, primary and universal dose reductions of targeted drugs, if enforced by Optimus, may potentially attenuate efficacy, as indicated, for example, ex negativo by the clinical benefit of escalating even traditional full doses from the pre-Optimus era: e.g., in ALK+ NSCLC itself, increasing the dose of alectinib beyond 600 mg twice daily can restore intracranial disease control [101]. Similarly, patients with metastatic gastrointestinal stromal tumors (GIST) progressing under 400 mg imatinib daily dose also experience new responses upon escalation to 800 mg [102], while patients with metastatic GIST starting treatment with 400 mg imatinib daily showed significantly shorter PFS (but not OS) compared to 800 mg in a randomized study [103]. Dose intensity has been associated with better clinical efficacy also in other settings, e.g., longer PFS for renal cell carcinoma under sunitinib in retrospective analyses [104,105], or higher icORR of 40% (4/10) with 300 mg/d vs. 0% (0/5) with 200 mg/d of sunvozertinib in WU-KONG1B [96]. Instead of a preemptive universal dose reduction for all patients, the CROWN experience and 7-year update demonstrate the merits of a more individualized approach, with dose reductions only as necessary due to emerging AE [106,107]. Full adoption of the new paradigm and commitment to achieving the PFS plateau will likely require even more flexible and individualized TKI dosing in the future, which only therapeutic drug monitoring (TDM) can offer [108]. Otherwise, we may never be able to make the necessary dose adjustments for many patients who may need them. One potential use case for TDM is the emerging challenge of ensuring adequate therapeutic drug levels over the several years of continuous treatment within the new paradigm, despite potential interferences, e.g., from altered metabolism or interactions, whose probability increases over time (Table 3). Maintaining proper exposure could also help improve safety, as an upward drift of plasma levels for lorlatinib increases the likelihood of side effects, such as hyperlipidemia or neurologic problems [90,109], while patients suffering from grade 3+ neurotoxicity are characterized by higher plasma concentrations of the drug [110].

Another important issue concerns the design of clinical trials. With drugs of extraordinary efficacy, like lorlatinib, as the new standard of care, evaluation of novel TKI against them will need to rely on alternative endpoints, such as landmark PFS rates instead of the mPFS, or the “slope” endpoints PFSDR/PDR, which are obtainable within a reasonable time and more robust (Section 2.3). In addition, more sample size-efficient designs will be necessary to reduce the number of patients required for establishing superiority or futility, while maintaining control of statistical error [111]. The impracticability of conventional clinical trials within the new paradigm is illustrated by the fact that a CROWN-like study (with 1:1 randomization, 90% power, enrollment of approximately 160 patients per year across 104 centers in 23 countries, and an event-driven primary analysis [21]), assuming proportional hazards, no loss to follow-up, and a control-arm mPFS of 9 years, would require 846 events and enrollment of, e.g., 960 patients over 6 years to provide results after a further 24.8 years for the non-inferiority of an equally active novel TKI using an HR margin of 1.25 and a one-sided a = 0.025. Alternatively, 508 events would be required, with, e.g., 640 patients enrolled over 4 years, to provide results after a further 21.9 years for the superiority of a novel TKI with a true HR of 0.75 vs. lorlatinib using a two-sided a = 0.05 (details in the Supplementary Material).

## 5. What the New Path Forward Now Looks Like

### 5.1. Combined ALK-Directed Approaches

If prevention rather than treatment of acquired resistance in ALK+ NSCLC is becoming the main goal within the new paradigm, we need to redefine our priorities accordingly. Based on the experience from CROWN, three properties of an ALK inhibitor are necessary and sufficient to this end: (i) strong activity against WT ALK; (ii) good brain penetration; and (iii) activity against single *ALK* resistance mutations, including the p.G1202R. In contrast, the activity against *ALK* compound mutations or p.L1256F seems to be irrelevant, because these apparently cannot arise under a drug like lorlatinib, which fulfills the three essential conditions; also, separate coverage of *EML4-ALK* V3 and/or *TP53*^mut^ can be removed from the previous agenda [112] as redundant, because a deep suppression of WT ALK already achieves this, according to the CROWN results (Section 3.2 and Section 3.3). Lorlatinib and CROWN are of paramount importance not only due to the unprecedented patient outcomes, but also because they illuminate the path forward.

To further improve survival beyond what is possible with currently available TKI monotherapies, combination regimens will be necessary. Considering the key role of ALK signaling as the common root of on-target and off-target resistance (Figure 2), the most reasonable next step would be to combine lorlatinib with a second, synergistic ALK-directed drug. One promising candidate for this would be the TPD TRI-611, which has demonstrated potency against WT and mutated ALK, intracranial activity and synergy with lorlatinib in preclinical models (presented as abstract) [82]. By achieving even deeper ALK suppression, this combination could hopefully stifle the small residual “leak” of acquired resistance observed after the third year in CROWN and facilitate total deactivation of ALK+ NSCLC in the late phase (Figure 2). Chemotherapy, on the other hand, is a less attractive partner, because benefits from it in stage IV NSCLC have generally been transient, and because its genotoxic effect could jeopardize the low TMB, which has been highlighted as a key favorable molecular characteristic of the ALK+ disease in CROWN and previous analyses (e.g., in abstract form from the ABP study, NCT04318938) [4,17,113].

Is it realistic to expect that the combination of lorlatinib with another ALK-specific drug could indeed eliminate late off-target resistance and completely deactivate the disease for the > 50% of patients with more favorable biology who enter the plateau phase after the third year? It appears to be, because even lorlatinib alone could demonstrate a pCR rate of almost 50% in the neoadjuvant LORIN trial [47], and because we have already seen that chronic myeloid leukemia, another malignancy with low TMB and strong oncogenic addiction, can be controlled indefinitely by a single TKI in most patients [114].

### 5.2. Next-Generation Immunotherapies for ALK+ NSCLC

Nevertheless, the exclusive targeting of ALK alone is unlikely to help the remaining 20–35% of ALK+ patients who fail lorlatinib earlier, during the first 1–3 years of treatment (columns 2 and 3 of Table 2). Emerging data from the CROWN and other studies reveal that patients with early progression under next-generation ALK TKI have more genetically unstable tumors with a higher frequency of *TP53* mutations and TMB, which correlate with each other [4,17,113]. Furthermore, emerging clinical and preclinical data suggest that alterations of other tumor suppressor genes (TSG), such as *CDKN2A/B*, *PTEN*, *RB1*, and *STK11*, which are less frequent and therefore challenging to analyze in clinical cohorts, are also associated with higher aggressiveness and increased tumor progression risk [115,116]. Given the complex biology of early progressors, narrow targeting of ALK itself alone will probably not be sufficient for durable tumor control. The most promising TKI partner are immunotherapies, which are generally known to work better for tumors with a higher TMB, as they use the entirety of genetic alterations as a broader attack surface [117]. While PD-(L)1 inhibitors are not active in ALK+ NSCLC due to particular properties of the tumor microenvironment [118], immunogenic peptides derived from WT ALK have been successfully used for vaccination in preclinical models and resulted in improved systemic and intracranial control of ALK-driven tumors synergistically with lorlatinib [119]. Other genetic alterations, such as specific *TP53* mutations and private or shared neoantigens, could be used as additional targets, as these have also been successfully exploited to control metastatic tumors using genetically engineered TCR-T cells in several proof-of-concept studies [120,121]. Besides the anticipated greater activity against early off-target resistance in the most aggressive tumors, which are also more immunogenic due to their higher TMB (Figure 2), novel immunotherapies could also complement ALK-directed drugs in eliminating the residual late escape during the plateau phase, thereby facilitating cure of more patients with ALK+ NSCLC.

## 6. Limitations

As the new paradigm for ALK+ NSCLC continues to emerge, some results from the CROWN study are not yet available, and the number of patients who have received lorlatinib for extended periods is still limited. Consequently, some parameters, like the conditional PFSDR and PDR, could only be estimated approximately, while the long-term safety profile remains incompletely characterized. Furthermore, the full spectrum of acquired resistance mechanisms to lorlatinib and the distinct biological properties of tumors selected to enter the late plateau phase cannot yet be fully defined due to the small number of patients with available molecular profiling and the technical limitations of ctDNA assays used in CROWN. In addition, some findings discussed in this review, including the resistance mechanisms identified in MARIPOSA and FLAURA2, results from the LORIN, ALK mutation neopeptide vaccination (ARCHER), ALKOVE-1, ARROS-1, and ABP studies, preclinical TRI-611 data, the 7-year ALESIA update, updated CROWN PFS curves according to molecular features, and the CROWN patients with false-positive immunohistochemistry, have thus far been reported only as congress abstracts or presentations. Finally, cross-trial comparisons of PFS and OS have inherent limitations and should be interpreted cautiously, particularly because most 2G ALK TKI trials have not reported updated PFS results beyond the third follow-up year.

## 7. Conclusions

The 7-year CROWN update is the most impressive milestone in ALK+ NSCLC since discovery of the oncogene 20 years ago and radically changes our paradigm. For the first time ever, a targeted drug eliminates acquired on-target resistance to abolish TKI sequencing and creates a PFS plateau that de facto indicates a survival advantage over alternative treatments even before OS in the corresponding study becomes formally evaluable. In the new era of extraordinary drug efficacy, drug development, the design of clinical trials, pathways of marketing authorization, and dosing optimization policies will need to be reconsidered. Lorlatinib will dominate the therapeutic landscape of ALK+ NSCLC for the next years until the advent of combination therapies, which are the most reasonable next step. Universal state-of-the-art DNA and RNA NGS for all newly diagnosed advanced NSCLC is now even more important in order to identify and treat ALK+ patients accordingly. Recent therapeutic advances will hopefully also spread to earlier-stage NSCLC and be complemented by novel targeted and immunotherapeutic approaches to further improve long-term control and facilitate cure of ALK-driven cancer.

## Figures and Tables

**Table 1:** The “rule-of-two” for the exponential increase in efficacy across the three ALK TKI generations is based on the HR for PFS in the respective randomized phase 3 first-line studies (in bold).

Drug (TKI Generation)	Comparator	PFS HR	mPFS (mo) *	ORR%	icORR% ^$^	CR%	icCR ^$^	Ref.
Crizotinib (1G)	chemotherapy	**0.45**	10.9	74%	23–50%	2%	0–8%	[22]
Alectinib (2G)	crizotinib	**0.50**	25.7	83%	81%	4%	38%	[18]
Brigatinib (2G)	crizotinib	**0.49**	24.0	71%	78%	4%	11%	[20]
Lorlatinib (3G)	crizotinib	**0.28**	n.r.	76%	82%	3%	71%	[21]

* according to the independent review committee (IRC); ^$^ among patients with measurable brain lesions (NB. for crizotinib: from the control arms of 2/3G studies). n.r.: not reached; Ref.: reference.

**Table 3:** The new paradigm for drugs with extraordinary efficacy after CROWN (on the left) contrasted to the current paradigm for targeted drugs (on the right). For the element of each line, further details are provided in the article section indicated in parentheses.

New Paradigm	Old Paradigm
Main treatment goal = disease inactivation (Section 2.3 and Section 4.3)	Main goal = quality of life + survival prolongation
Long-term disease control with a single ALK TKI line (Section 4.1)	TKI sequencing to maximize survival
New (combination) regimens to prevent acquired resistance (Section 5.1 and Section 5.2)	Next-generation TKI to treat acquired resistance
Ability to prevent ≠ ability to treat resistance (Section 3.2)	The tackling of resistance as a single drug property
Landmark PFS rates and slope endpoints: PFSDR/PDR (Section 2.3 and Section 4.3)	ORR, mPFS, mOS as main efficacy endpoints
OS superiority demonstrated by PFS before maturity ^1^ (Section 2.2)	OS superiority requires analysis of OS after PFS
Excellent “soon-after-first” new drugs may fail ^1^ (Section 4.2)	Better new drugs replace older drugs in 2L and then 1L
Individualized dosing to aim for the PFS plateau ^2^ (Section 4.3)	Universal lower doses to improve safety (Optimus)
Earlier readout, more sample-size efficient designs ^2^ (Section 4.3)	Strict event maturity + superiority/inferiority testing

^1^ Temporary “irregularities” during the transitional time; ^2^ Future changes that will gradually become inevitable.

## Data Availability

No new data were created or analyzed in this study. Data sharing is not applicable to this article.

## Supplementary Materials

The following supporting information can be downloaded at: https://www.mdpi.com/article/10.3390/cancers18183044/s1, File S1: Supplementary Material; File S2: Non-inferiority calculation; File S3: Superiority calculation. References [122,123,124,125,126,127] are cited in the Supplementary Materials.

## Abbreviations

The following abbreviations are used in this manuscript:

ALK	anaplastic lymphoma kinase
TKI	tyrosine kinase inhibitors
NSCLC	non-small-cell lung cancer
TMB	tumor mutational burden
LAT	local ablative therapy
(m)OS	(median) overall survival
EML4	echinoderm microtubule-associated protein-like 4
TP53	tumor protein 53
NGS	next-generation sequencing
CNA	copy number alterations
CT	computed tomography
MRI	magnetic resonance imaging
PD-(L)1	programmed death-ligand 1
ADC	antibody–drug conjugate
CHT	chemotherapy
IO	immunooncological treatments (immunotherapies)
GIST	gastrointestinal stromal tumor
BM	brain metastases
V	variant
G	generation
DNA	deoxyribonucleic acid
RNA	ribonucleic acid
ctDNA	circulating tumor DNA
cfDNA	cell-free DNA
(m)PFS(DR)	(median) progression-free survival (drop rate)
PD(R)	progressive disease (rate)
HR	hazard ratio
(ic)ORR	(intracranial) overall response rate
(ic/p)CR	(intracranial/pathologic) complete remission
IRC	independent review committee
Ref	reference
n/a	not available or not applicable
n.r.	not reached
AE	adverse events
MRD	minimal-residual disease
WT	wild-type
IC50	half maximal inhibitory concentration
EGFR	epidermal growth factor receptor
MET	MNNG human osteosarcoma experimental transforming
MNNG	N-methyl-N′-nitro-N-nitrosoguanidine
TPD	targeted protein degrader
RP2D	recommended phase 2 dose
ROS1	rat osteogenic sarcoma 1
TDM	therapeutic drug monitoring
FDA	United States Food and Drug Administration
NMPA	Chinese National Medical Products Administration
TSG	tumor suppressor gene

## References

[1] Fan L., Feng Y., Wan H., Shi G., Niu W. (2014). Clinicopathological and Demographical Characteristics of Non-Small Cell Lung Cancer Patients with ALK Rearrangements: A Systematic Review an d Meta-Analysis. PLoS ONE.

[2] Sabir S.R., Yeoh S., Jackson G., Bayliss R. (2017). EML4-ALK Variants: Biological and Molecular Properties, and the Implications for Patients. Cancers.

[3] Soda M., Choi Y.L., Enomoto M., Takada S., Yamashita Y., Ishikawa S., Fujiwara S., Watanabe H., Kurashina K., Hatanaka H. (2007). Identification of the transforming EML4-ALK fusion gene in non-small-cell lung cancer. Nature.

[4] Christopoulos P., Budczies J., Kirchner M., Dietz S., Sültmann H., Thomas M., Stenzinger A. (2019). Defining molecular risk in ALK+ NSCLC. Oncotarget.

[5] Hendriks L.E., Cortiula F., Mariamidze E., Martins Branco D., Passaro A., Reguart N., Reck M. (2026). ESMO Oncogene-Addicted Non-Small Cell Lung Cancer Living Guideline v1. 3. https://www.esmo.org/guidelines/living-guidelines/esmo-living-guideline-oncogene-addicted-metastatic-non-small-cell-lung-cancer.

[6] Lin J.J., Riely G.J., Shaw A.T. (2017). Targeting ALK: Precision Medicine Takes on Drug Resistance. Cancer Discov..

[7] Elsayed M., Christopoulos P. (2021). Therapeutic Sequencing in ALK+ NSCLC. Pharmaceuticals.

[8] Mok T., Camidge D.R., Gadgeel S.M., Rosell R., Dziadziuszko R., Kim D.W., Pérol M., Ou S.H.I., Ahn J.S., Shaw A.T. (2020). Updated overall survival and final progression-free survival data for patients with treatment-naive advanced ALK-positive non-small-cell lun g cancer in the ALEX study. Ann. Oncol..

[9] Ettinger D.S., Wood D.E., Aisner D.L., Akerley W., Bauman J., Chirieac L.R., D’Amico T.A., DeCamp M.M., Dilling T.J., Dobelbower M. (2017). NCCN Clinical Practice Guidelines in Oncology: Non-Small Cell Lung Cancer. Version 2.2018.

[10] McCoach C.E., Blakely C.M., Banks K.C., Levy B., Chue B.M., Raymond V.M., Le A.T., Lee C.E., Diaz J., Waqar S.N. (2018). Clinical Utility of Cell-Free DNA for the Detection of ALK Fusions and Genomic Mechanisms of ALK Inhibitor Resistance in Non-Small Cell Lung Cancer. Clin. Cancer Res..

[11] Horn L., Whisenant J.G., Wakelee H., Reckamp K.L., Qiao H., Leal T.A., Du L., Hernandez J., Huang V., Blumenschein G.R. (2019). Monitoring Therapeutic Response and Resistance: Analysis of Circulating Tumor DNA in Patients With ALK+ Lung Cancer. J. Thorac. Oncol..

[12] Camidge D.R., Kim H.R., Ahn M.-J., Yang J.C.H., Han J.-Y., Hochmair M.J., Lee K.H., Delmonte A., Garcia Campelo M.R., Kim D.-W. (2021). Brigatinib Versus Crizotinib in ALK Inhibitor-Naive Advanced ALK-Posit ive NSCLC: Final Results of Phase 3 ALTA-1L Trial. J. Thorac. Oncol..

[13] Dietz S., Christopoulos P., Yuan Z., Angeles A.K., Gu L., Volckmar A.-L., Ogrodnik S.J., Janke F., Fratte C.D., Zemojtel T. (2020). Longitudinal therapy monitoring of ALK-positive lung cancer by combine d copy number and targeted mutation profiling of cell-free DNA. EBioMedicine.

[14] Angeles A.K., Christopoulos P., Yuan Z., Bauer S., Janke F., Ogrodnik S.J., Reck M., Schlesner M., Meister M., Schneider M.A. (2021). Early identification of disease progression in ALK-rearranged lung can er using circulating tumor DNA analysis. npj Prec. Oncol..

[15] Angeles A.K., Janke F., Daum A.-K., Reck M., Schneider M.A., Thomas M., Christopoulos P., Sültmann H. (2023). Integrated circulating tumour DNA and cytokine analysis for therapy monitoring of ALK-rearranged lung adenocarcinoma. Br. J. Cancer.

[16] Kwon M., Ku B.M., Olsen S., Park S., Lefterova M., Odegaard J., Jung H.-A., Sun J.-M., Lee S.-H., Ahn J.S. (2022). Longitudinal monitoring by next-generation sequencing of plasma cell-free DNA in ALK rearranged NSCLC patients treated with ALK tyrosine kin ase inhibitors. Cancer Med..

[17] Shaw A.T., Solomon B.J., Felip E., Bauer T.M., Liu G., Goto Y., Wu Y.L., Soo R.A., Mazieres J., Kim D.W. (2026). Lorlatinib versus crizotinib as first-line treatment for advanced ALK- positive non-small-cell lung cancer: 7-year update from the phase III CROWN study. Ann. Oncol..

[18] Peters S., Camidge D.R., Shaw A.T., Gadgeel S., Ahn J.S., Kim D.-W., Ou S.-H.I., Pérol M., Dziadziuszko R., Rosell R. (2017). Alectinib versus Crizotinib in Untreated ALK-Positive Non-Small-Cell Lung Cancer. N. Engl. J. Med..

[19] Shaw A.T., Varghese A.M., Solomon B.J., Costa D.B., Novello S., Mino-Kenudson M., Awad M.M., Engelman J.A., Riely G.J., Monica V. (2013). Pemetrexed-based chemotherapy in patients with advanced, ALK-positive non-small cell lung cancer. Ann. Oncol..

[20] Camidge D.R., Kim H.R., Ahn M.-J., Yang J.C.-H., Han J.-Y., Lee J.-S., Hochmair M.J., Li J.Y.-C., Chang G.-C., Lee K.H. (2018). Brigatinib versus Crizotinib in ALK-Positive Non-Small-Cell Lung Cancer. N. Engl. J. Med..

[21] Shaw A.T., Bauer T.M., Marinis F., Felip E., Goto Y., Liu G., Mazieres J., Kim D.-W., Mok T., Polli A. (2020). First-Line Lorlatinib or Crizotinib in Advanced ALK-Positive Lung Cancer. N. Engl. J. Med..

[22] Solomon B.J., Mok T., Kim D.-W., Wu Y.-L., Nakagawa K., Mekhail T., Felip E., Cappuzzo F., Paolini J., Usari T. (2014). First-Line Crizotinib versus Chemotherapy in ALK-Positive Lung Cancer. N. Engl. J. Med..

[23] Soria J.-C., Ohe Y., Vansteenkiste J., Reungwetwattana T., Chewaskulyong B., Lee K.H., Dechaphunkul A., Imamura F., Nogami N., Kurata T. (2018). Osimertinib in Untreated EGFR-Mutated Advanced Non-Small-Cell Lung Cancer. N. Engl. J. Med..

[24] Solomon B.J., Bauer T.M., Mok T.S.K., Liu G., Mazieres J., Marinis F., Goto Y., Kim D.-W., Wu Y.-L., Jassem J. (2023). Efficacy and safety of first-line lorlatinib versus crizotinib in patients with advanced, ALK-positive non-small-cell lung cancer: Updated analysis of data from the phase 3, randomised, open-label CROWN study. Lancet Respir. Med..

[25] Mok T., Solomon B.J., Campelo M.R.G., Wu Y.L., Streich G., Zemanova M., Zalcman G., Bearz A., Chang G.C., Setti M. (2024). MA06.07 Patterns of Progression with Lorlatinib and Insights into Subsequent Anticancer Therapy Efficacy in Advanced ALK+ NSCLC. J. Thorac. Oncol..

[26] Solomon B.J., Liu G., Felip E., Mok T.S.K., Soo R.A., Mazieres J., Shaw A.T., Marinis F., Goto Y., Wu Y.-L. (2024). Lorlatinib Versus Crizotinib in Patients With Advanced ALK-Positive Non-Small Cell Lung Cancer: 5-Year Outcomes From the Phase III CROWN Study. J. Clin. Oncol..

[27] Solomon B.J., Liu G., Felip E., Mok T.S.K., Soo R.A., Mazieres J., Shaw A.T., Marinis F., Goto Y., Wu Y.-L. (2024). Lorlatinib vs crizotinib in treatment-naïve patients with advanced ALK+ non-small cell lung cancer: 5-year progression-free survival and safety from the CROWN study. J. Clin. Oncol..

[28] United Nations, Department of Economic and Social Affairs Population Division World Population Prospects 2024, Online Edition. https://population.un.org/wpp/downloads?folder=Standard%20Projections&group=Mortality.

[29] Cali Daylan A.E., Halmos B. (2023). Long-term benefit of immunotherapy in metastatic non-small cell lung cancer: The tale of the tail. Transl. Lung Cancer Res..

[30] Frost N., Joosten M., Franzen J., Wiesweg M., Rasokat A., Kulhavy J., Kollmeier J., Reinmuth N., Grohé C., Roeper J. (2026). PET/CT-guided management of immune checkpoint blockade and multi-modal profiling following treatment in long-term responders with metastatic lung cancer in the National Network Genomic Medicine Lung Cancer Germany (nNGM). Ann. Oncol..

[31] Bauer T., Abrahami D., Polli A., Chu H., Ramachandran P., Chandler C., Tan M., Truscott J., Mazieres J., Garcia C.A. (2026). Long-term efficacy and safety of lorlatinib versus alectinib in anaplastic lymphoma kinase-positive advanced/metastatic non-small cell lung cancer: Matching-adjusted indirect comparison. J. Comp. Eff. Res..

[32] Mologni L. (2015). Expanding the portfolio of anti-ALK weapons. Transl. Lung Cancer Res..

[33] Soria J.-C., Tan D.S.W., Chiari R., Wu Y.-L., Paz-Ares L., Wolf J., Geater S.L., Orlov S., Cortinovis D., Yu C.-J. (2017). First-line ceritinib versus platinum-based chemotherapy in advanced ALK-rearranged non-small-cell lung cancer (ASCEND-4): A randomised, open-label, phase 3 study. Lancet.

[34] Nakagawa K., Hida T., Nokihara H., Morise M., Azuma K., Kim Y.H., Seto T., Takiguchi Y., Nishio M., Yoshioka H. (2020). Final progression-free survival results from the J-ALEX study of alectinib versus crizotinib in ALK-positive non-small-cell lung cancer. Lung Cancer.

[35] Kuhn T.S. (1970). The Structure of Scientific Revolutions.

[36] Peters S., Camidge R., Dziadziuszko R., Gadgeel S., Shaw A.T., Kim D.W., Pérol M., Rosell D.R., Cheema P., Wan-Teck Lim D. (2026). Alectinib versus crizotinib in previously untreated ALK-positive advanced non-small cell lung cancer: Final overall survival analysis of the phase III ALEX study. Ann. Oncol..

[37] Zhao M., Shao T., Shao H., Zhou C., Tang W. (2024). Identifying optimal ALK inhibitors in first- and second-line treatment of patients with advanced ALK-positive non-small-cell lung cancer: A systematic review and network meta-analysis. BMC Cancer.

[38] Zheng B., Jiang H., Yang W., Li Y., Liang B., Zhu J., Chen N., Chen M., Zhang M. (2023). A Bayesian network meta-analysis of ALK inhibitor treatments in patients with ALK-positive non-small cell lung cancer. Cancer Med..

[39] Hotta K., Hida T., Nokihara H., Morise M., Kim Y.H., Azuma K., Seto T., Takiguchi Y., Nishio M., Yoshioka H. (2022). Final overall survival analysis from the phase III J-ALEX study of alectinib versus crizotinib in ALK inhibitor-naïve Japanese patients with ALK-positive non-small-cell lung cancer. ESMO Open.

[40] Zhou C., Lu Y., Kim S.-W., Reungwetwattana T., Zhou J., Zhang Y., He J., Yang J.-J., Cheng Y., Lee S.-H. (2024). Alectinib Versus Crizotinib in Asian Patients With Treatment-Naïve Advanced ALK-Positive NSCLC: Five-Year Update From the Phase 3 ALESIA Study. JTO Clin. Res. Rep..

[41] Zhou C., Lu Y., Kim S.W., Baisamut T.R., Zhou J., Zhang Y., He J., Yang J.J., Cheng Y., Lee S.H. (2024). 629MO ALESIA 7-year update: Alectinib vs crizotinib in Asian patients (pts) with treatment-naive advanced ALK+ non-small cell lung cancer. Ann. Oncol..

[42] Ecclestone C., Chow R., Pulenzas N., Zhang L., Leahey A., Hamer J., DeAngelis C., Bedard G., McDonald R., Bhatia A. (2016). Quality of life and symptom burden in patients with metastatic breast cancer. Support. Care Cancer.

[43] Albertsen P.C., Aaronson N.K., Muller M.J., Keller S.D., Ware J.E. (1997). Health-related quality of life among patients with metastatic prostate cancer. Urology.

[44] Evren I., Brouns E.R., Wils L.J., Poell J.B., Peeters C.F.W., Brakenhoff R.H., Bloemena E., Visscher J.G.A.M. (2020). Annual malignant transformation rate of oral leukoplakia remains consistent: A long-term follow-up study. Oral Oncol..

[45] Akhlaghi T., Maclachlan K.H., Wiggins A., Usmani S.Z., Derkach A., Hultcrantz M. (2025). Prognosis and patterns of progression in smoldering multiple myeloma. Blood Adv..

[46] Leonetti A., Boni L., Gnetti L., Cortinovis D.L., Pasello G., Mazzoni F., Bearz A., Gelsomino F., Passiglia F., Pilotto S. (2025). Alectinib as neoadjuvant treatment in potentially resectable stage III ALK-positive NSCLC: Final analysis of ALNEO phase II trial (GOIRC-01- 2020-ML42316). J. Clin. Oncol..

[47] Zhang C., Hu Y., Jiang B.-Y., Yan L., Ren S.-Y., Chen J.-H., Zhou Q., Li D.-Q., Liu W., Wu Y.-L. (2026). Neoadjuvant lorlatinib in stage III NSCLC harboring ALK fusion: A phase 2 multicenter study (LORIN). J. Clin. Oncol..

[48] Campelo R.G., Schulz C., Min Y.J., Aydiner A., Hussein M.A., Pietro A., Polli A., Martini J.-F., Kim H.R. (2025). Depth of response and progression-free survival in patients with advanced ALK-positive non–small-cell lung cancer treated with lorlatinib. J. Clin. Oncol..

[49] Camidge D.R., Kim H.R., Ahn M.-J., Yang J.C.-H., Han J.-Y., Hochmair M., Lee K.H., Delmonte A., Campelo R.G., Kim D.-W. (2022). Association of depth of target lesion response to brigatinib with outcomes in patients with ALK inhibitor-naive ALK+ NSCLC in ALTA-1L. J. Clin. Oncol..

[50] Soo R.A., Martini J.-F., Wekken A.J., Teraoka S., Ferrara R., Shaw A.T., Shepard D., Calella A.M., Polli A., Toffalorio F. (2023). Early Circulating Tumor DNA Dynamics and Efficacy of Lorlatinib in Patients With Treatment-Naive, Advanced, ALK-Positive NSCLC. J. Thorac. Oncol..

[51] Christopoulos P. (2023). An ever-stronger case for circulating tumor DNA monitoring in ALK+ lung cancer. AME Clin. Trials Rev..

[52] Tunali I., Gillies R.J., Schabath M.B. (2021). Application of Radiomics and Artificial Intelligence for Lung Cancer Precision Medicine. Cold Spring Harb. Perspect. Med..

[53] Wang Y., Shao W., Li H., Zhao P., Tian L., Zhang L., Lan S., Zhong R., Zhang S., Cheng Y. (2025). Clinical application of minimal residual disease detection by ctDNA testing in non-small cell lung cancer: A narrative review. Transl. Lung Cancer Res..

[54] Dimitrakopoulou-Strauss A., Sachpekidis C., Hassel J.C., Christopoulos P. (2023). Positron Emission Tomography-Based Immunoimaging for Cancer Patient Stratification: Toward a More Holistic Approach. Cancer Biother. Radiopharm..

[55] Schütte W., Gütz S., Nehls W. S3 Leitlinie: Prävention, Diagnostik, Therapie und Nachsorge des Lungenkarzinoms. https://www.leitlinienprogramm-onkologie.de/leitlinien/lungenkarzinom/.

[56] National Comprehensive Cancer Network (2026). NCCN Clinical Practice Guidelines in Oncology (NCCN Guidelines): Non-Small Cell Lung Cancer.

[57] Shiba-Ishii A., Johnson T.W., Dagogo-Jack I., Mino-Kenudson M., Johnson T.R., Wei P., Weinrich S.L., McTigue M.A., Walcott M.A., Nguyen-Phuong L. (2022). Analysis of lorlatinib analogs reveals a roadmap for targeting diverse compound resistance mutations in ALK-positive lung cancer. Nat. Cancer.

[58] Gainor J.F., Dardaei L., Yoda S., Friboulet L., Leshchiner I., Katayama R., Dagogo-Jack I., Gadgeel S., Schultz K., Singh M. (2016). Molecular Mechanisms of Resistance to First- and Second-Generation ALK Inhibitors in ALK-Rearranged Lung Cancer. Cancer Discov..

[59] Yoda S., Lin J.J., Lawrence M.S., Burke B.J., Friboulet L., Langenbucher A., Dardaei L., Prutisto-Chang K., Dagogo-Jack I., Timofeevski S. (2018). Sequential ALK Inhibitors Can Select for Lorlatinib-Resistant Compound ALK Mutations in ALK-Positive Lung Cancer. Cancer Discov..

[60] Okada K., Araki M., Sakashita T., Ma B., Kanada R., Yanagitani N., Horiike A., Koike S., Oh-hara T., Watanabe K. (2019). Prediction of ALK mutations mediating ALK-TKIs resistance and drug re-purposing to overcome the resistance. EBioMedicine.

[61] Lin J.J., Horan J.C., Tangpeerachaikul A., Swalduz A., Valdivia A., Johnson M.L., Besse B., Camidge D.R., Fujino T., Yoda S. (2024). NVL-655 Is a Selective and Brain-Penetrant Inhibitor of Diverse ALK-Mutant Oncoproteins, Including Lorlatinib-Resistant Compound Mutations. Cancer Discov..

[62] Lin J.J., Zhu V.W., Yoda S., Yeap B.Y., Schrock A.B., Dagogo-Jack I., Jessop N.A., Jiang G.Y., Le L.P., Gowen K. (2018). Impact of EML4-ALK Variant on Resistance Mechanisms and Clinical Outcomes in ALK-Positive Lung Cancer. J. Clin. Oncol..

[63] Christopoulos P., Kirchner M., Endris V., Stenzinger A., Thomas M. (2018). EML4-ALK V3, treatment resistance, and survival: Refining the diagnosis of ALK+ NSCLC. J. Thor. Dis..

[64] Woo C.G., Seo S., Kim S.W., Jang S.J., Park K.S., Song J.Y., Lee B., Richards M.W., Bayliss R., Lee D.H. (2017). Differential protein stability and clinical responses of EML4-ALK fusion variants to various ALK inhibitors in advanced ALK-rearranged non-s mall cell lung cancer. Ann. Oncol..

[65] Christopoulos P., Endris V., Bozorgmehr F., Elsayed M., Kirchner M., Ristau J., Buchhalter I., Penzel R., Herth F.J., Heussel C.P. (2018). EML4-ALK fusion variant V3 is a high-risk feature conferring accelerated metastatic spread, early treatment failure and worse overall survival in ALK+ non-small cell lung cancer. Int. J. Cancer.

[66] Richards M.W., Law E.W.P., Rennalls L.V.P., Busacca S., O’Regan L., Fry A.M., Fennell D.A., Bayliss R. (2014). Crystal structure of EML1 reveals the basis for Hsp90 dependence of oncogenic EML4-ALK by disruption of an atypical β-propeller domain. Proc. Natl. Acad. Sci. USA.

[67] O’Regan L., Barone G., Adib R., Woo C.G., Jeong H.J., Richardson E.L., Richards M.W., Muller P.A.J., Collis S.J., Fennell D.A. (2020). EML4-ALK V3 oncogenic fusion proteins promote microtubule stabilizatio n and accelerated migration through NEK9 and NEK7. J. Cell Sci..

[68] Dornburg A., Wang Z., Wang J., Mo E.S., López-Giráldez F., Townsend J.P. (2021). Comparative Genomics within and across Bilaterians Illuminates the Evolutionary History of ALK and LTK Proto-Oncogene Origination and Divers ification. Genome Biol. Evol..

[69] Lin J.J., Felip E., Cho B.C., Besse B., Liu G., Landi L., Paz-Ares L.G., Bennati C., Swalduz A., Popat S. (2026). ALKOVE-1: Efficacy and safety of neladalkib in patients with advanced ALK+ NSCLC. J. Clin. Oncol..

[70] Felip E., Shaw A.T., Bearz A., Camidge D.R., Solomon B.J., Bauman J.R., Bauer T.M., Peters S., Toffalorio F., Abbattista A. (2021). Intracranial and extracranial efficacy of lorlatinib in patients with ALK-positive non-small-cell lung cancer previously treated with second-generation ALK TKIs. Ann. Oncol..

[71] Camidge D.R., Dziadziuszko R., Peters S., Mok T., Noe J., Nowicka M., Gadgeel S.M., Cheema P., Pavlakis N., Marinis F. (2019). Updated Efficacy and Safety Data and Impact of the EML4-ALK Fusion Var iant on the Efficacy of Alectinib in Untreated ALK-Positive Advanced N on-Small Cell Lung Cancer in the Global Phase III ALEX Study. J. Thorac. Oncol..

[72] Christopoulos P., Kirchner M., Bozorgmehr F., Endris V., Elsayed M., Budczies J., Ristau J., Penzel R., Herth F.J., Heussel C.P. (2019). Identification of a highly lethal V3+ TP53+ subset in ALK+ lung adenocarcinoma. Int. J. Cancer.

[73] Williams B.R., Prabhu V.R., Hunter K.E., Glazier C.M., Whittaker C.A., Housman D.E., Amon A. (2008). Aneuploidy affects proliferation and spontaneous immortalization in mammalian cells. Science.

[74] Tanimoto A., Matsumoto S., Takeuchi S., Arai S., Fukuda K., Nishiyama A., Yoh K., Ikeda T., Furuya N., Nishino K. (2021). Proteasome Inhibition Overcomes ALK-TKI Resistance in ALK-Rearranged/TP53-Mutant NSCLC via Noxa Expression. Clin. Cancer Res..

[75] Besse B., Lee S.H., Lu S., Stroyakovskiy D., Yazici O., Cid J.R.R., Hayashi H., Nguyen D., Yang J.C.H., Gottfried M. (2024). LBA55 Mechanisms of acquired resistance to first-line amivantamab plus lazertinib versus osimertinib in patients with EGFR-mutant advanced non-small cell lung cancer: An early analysis from the phase III MARIPOSA study. Ann. Oncol..

[76] Yang J.C., Robichaux J., Planchard D., Kobayashi K., Lee C.K., Sugawara S., Yang T.Y., Kim T.M., Kim S.W., Yanagitani N. (2024). MA12.03 FLAURA2: Resistance, and Impact of Baseline TP53 Alterations in Patients Treated With 1L Osimertinib ± Platinum-Pemetrexed. J. Thorac. Oncol..

[77] Desai A., Lovly C.M. (2023). Strategies to overcome resistance to ALK inhibitors in non-small cell lung cancer: A narrative review. Transl. Lung Cancer Res..

[78] Christopoulos P., Dietz S., Angeles A.K., Rheinheimer S., Kazdal D., Volckmar A.-L., Janke F., Endris V., Meister M., Kriegsmann M. (2021). Earlier extracranial progression and shorter survival in ALK-rearranged lung cancer with positive liquid rebiopsies. Transl. Lung Cancer Res..

[79] Dagogo-Jack I., Kiedrowski L.A., Heist R.S., Lin J.J., Meador C.B., Krueger E.A., Do A., Peterson J., Sequist L.V., Gainor J.F. (2023). Efficacy and Tolerability of ALK/MET Combinations in Patients With ALK-Rearranged Lung Cancer With Acquired MET Amplification: A Retrospective Analysis. JTO Clin. Res. Rep..

[80] Hu D., Hu Y., Lei S., Wu D., Wang Y. (2025). MET tyrosine kinase inhibitors in combination with EGFR tyrosine kinase inhibitors in NSCLC patients with EGFR mutations and acquired MET alterations: A systematic review and meta-analysis. BMC Cancer.

[81] Browning E.T., Weickhardt A.J., Camidge D.R. (2013). Response to Crizotinib Rechallenge after Initial Progression and Intervening Chemotherapy in ALK Lung Cancer. J. Thorac. Oncol..

[82] Conery A.R., La D.S., Alekseyenko A.A., Marcoux D., Bart A.G., Harlow M.L., Arsenault P.R., Cantone N.R., Casaubon R.L., Kamadurai H.B. (2026). Abstract 5787: TRI-611, a development stage molecular glue degrader of ALK, promotes the degradation of TKI-resistant ALK fusion proteins an d leads to regression of ALK TKI-refractory tumors. Cancer Res..

[83] Conroy M.R., Wang Y., Vogelstein B., Murray J.C., Marrone K.A., Scott S.C., Feliciano J.L., Hann C.L., Thoburn C., Pabani A. (2026). Prophylactic peptide vaccine targeting resistance mutations in advance d ALK-positive lung cancer: Primary analysis from the ARCHER trial. J. Clin. Oncol..

[84] Lin J.J., Schoenfeld A.J., Zhu V.W., Yeap B.Y., Chin E., Rooney M., Plodkowski A.J., Digumarthy S.R., Dagogo-Jack I., Gainor J.F. (2020). Efficacy of Platinum/Pemetrexed Combination Chemotherapy in ALK-Positive NSCLC Refractory to Second-Generation ALK Inhibitors. J. Thorac. Oncol..

[85] Drilon A.E., Lin J.J., Johnson M.L., Baik C.S., Paz-Ares L.G., Besse B., Mazieres J., Swalduz A., Minchom A.R., Reuss J. (2024). 1253O Phase I/II ALKOVE-1 study of NVL-655 in ALK+ solid tumours. Ann. Oncol..

[86] Elsayed M., Bozorgmehr F., Kazdal D., Volckmar A.-L., Sültmann H., Fischer J.R., Kriegsmann M., Stenzinger A., Thomas M., Christopoulos P. (2021). Feasibility and Challenges for Sequential Treatments in ALK-Rearranged Non-Small-Cell Lung Cancer. Front. Oncol..

[87] Waliany S., Roy S.G., Pecci F., Weber U.M., Sun F., Arter Z., Lomibao M., Alessi J.V., Nishino M., Luo F. (2025). Efficacy and Safety of Continuing Next-Generation ALK TKIs With Chemotherapy for Advanced ALK-Positive NSCLC: A Multicenter Retrospective Study. J. Natl. Compr. Cancer Netw..

[88] Popat S., Solomon B.J., Stinchcombe T., Liu G., Lopes G., Johnson M.L., Nagasaka M., Cali Daylan E., Baik C.S., D’Olimpio J.T. (2025). Neladalkib (NVL-655), a highly selective anaplastic lymphoma kinase (A LK) inhibitor, compared to alectinib in first-line treatment of patients with ALK-positive advanced non-small cell lung cancer: The phase 3 ALKAZAR study. J. Clin. Oncol..

[89] Lin J.J., Johnson M., Felip E., Ou S.-H.I., Besse B., Baik C., Mazieres J., Elamin Y., Reuss J.E., Minchom A. (2023). Abstract B154: Safety and preliminary activity of the selective ALK inhibitor NVL-655 in patients with ALK fusion-positive solid tumors. Mol. Cancer Ther..

[90] Shaw A.T., Felip E., Bauer T.M., Besse B., Navarro A., Postel-Vinay S., Gainor J.F., Johnson M., Dietrich J., James L.P. (2017). Lorlatinib in non-small-cell lung cancer with ALK or ROS1 rearrangement: An international, multicentre, open-label, single-arm first-in-man phase 1 trial. Lancet Oncol..

[91] Johnson T.W., Richardson P.F., Bailey S., Brooun A., Burke B.J., Collins M.R., Cui J.J., Deal J.G., Deng Y.-L., Dinh D. (2014). Discovery of (10R)-7-Amino-12-fluoro-2,10,16-trimethyl-15-oxo-10,15,16,17-tetrahydro-2H-8,4-(metheno)pyrazolo-[4,3-h]-[2,5,11]-benzoxa-diaza-cyclo-tetradecine-3-carbonitrile (PF-06463922), a Macrocyclic Inhibitor of Anaplastic Lymphoma Kinase (ALK) and c-ros Oncogene 1 (ROS1) with Preclinical Brain Exposure and Broad-Spectrum Potency against ALK-Resistant Mutations. J. Med. Chem..

[92] Samaeekia R., Arter Z., Nagasaka M. (2026). An evaluation of taletrectinib for the treatment of ROS1+ non-small cell lung cancer. Expert Rev. Anticancer Ther..

[93] Drilon A.E., Cho B.C., Lin J.J., Solomon B.J., Lin C.C., Langen A.J., Felip E., Neal J.W., Liu S.V., Soo R.A. (2025). PL02.15 Pivotal ARROS-1 Efficacy and Safety Data: Zidesamtinib in TKI Pre-treated Patients with Advanced/Metastatic ROS1+ NSCLC. J. Thorac. Oncol..

[94] Drilon A., Horan J.C., Tangpeerachaikul A., Besse B., Ou S.-H.I., Gadgeel S.M., Camidge D.R., Wekken A.J., Nguyen-Phuong L., Acker A. (2023). NVL-520 Is a Selective, TRK-Sparing, and Brain-Penetrant Inhibitor of ROS1 Fusions and Secondary Resistance Mutations. Cancer Discov..

[95] Murphy R., Halford S., Symeonides S.N. (2023). Project Optimus, an FDA initiative: Considerations for cancer drug development internationally, from an academic perspective. Front. Oncol..

[96] Yang J.C.-H., Wang M., Doucet L., Fan Y., Lv D., Sun M., Huang D., Greillier L., Planchard D., Hong Q. (2025). Phase II Dose-Randomized Study of Sunvozertinib in Platinum-Pretreated Non-Small Cell Lung Cancer With Epidermal Growth Factor Receptor Exon 20 Insertion Mutations (WU-KONG1B). J. Clin. Oncol..

[97] Zhou C., Greillier L., Liu G., John T., Xing L., Kowalski D., Memmott Regan M., Yazici O., Sun M., Shu Catherine A. (2026). First-Line Sunvozertinib in NSCLC with EGFR Exon 20 Insertion Mutations. N. Engl. J. Med..

[98] Foo J., Chmielecki J., Pao W., Michor F. (2012). Effects of pharmacokinetic processes and varied dosing schedules on the dynamics of acquired resistance to erlotinib in EGFR-mutant lung cancer. J. Thorac. Oncol..

[99] Pirazzoli V., Politi K. (2014). Generation of drug-resistant tumors using intermittent dosing of tyrosine kinase inhibitors in mouse. Cold Spring Harb. Protoc..

[100] Hayakawa H., Ichihara E., Ohashi K., Ninomiya T., Yasugi M., Takata S., Sakai K., Matsumoto K., Takigawa N., Tanimoto M. (2013). Lower gefitinib dose led to earlier resistance acquisition before emergence of T790M mutation in epidermal growth factor receptor-mutated lung cancer model. Cancer Sci..

[101] Cheung J.M., Kang J., Yeap B.Y., Peterson J.L., Do A., Gainor J.F., Digumarthy S.R., Lin J.J. (2024). Efficacy and Safety of Dose-Escalated Alectinib in Patients With Metastatic ALK-Positive NSCLC and Central Nervous System Relapse on Standard-Dose Alectinib. JTO Clin. Res. Rep..

[102] Zalcberg J.R., Verweij J., Casali P.G., Le Cesne A., Reichardt P., Blay J.Y., Schlemmer M., Van Glabbeke M., Brown M., Judson I.R. (2005). Outcome of patients with advanced gastrointestinal stromal tumours crossing over to a daily imatinib dose of 800 mg after progression on 400 mg. Eur. J. Cancer.

[103] Verweij J., Casali P.G., Zalcberg J., LeCesne A., Reichardt P., Blay J.Y., Issels R., van Oosterom A., Hogendoorn P.C., Van Glabbeke M. (2004). Progression-free survival in gastrointestinal stromal tumours with high-dose imatinib: Randomised trial. Lancet.

[104] Raphael J., Thawer A., Bjarnason G.A. (2018). Sunitinib dose-escalation after disease progression in metastatic renal cell carcinoma. Urol. Oncol..

[105] Maraz A., Cserhati A., Uhercsak G., Szilagyi E., Varga Z., Revesz J., Koszo R., Varga L., Kahan Z. (2018). Dose escalation can maximize therapeutic potential of sunitinib in patients with metastatic renal cell carcinoma. BMC Cancer.

[106] Reed M., Rosales A.-L.S., Chioda M.D., Parker L., Devgan G., Kettle J. (2020). Consensus Recommendations for Management and Counseling of Adverse Eve nts Associated With Lorlatinib: A Guide for Healthcare Practitioners. Adv. Ther..

[107] Liu G., Mazieres J., Stratmann J., Ou S.-H.I., Mok T., Grizzard M., Goto Y., Felip E., Solomon B.J., Bauer T.M. (2024). A pragmatic guide for management of adverse events associated with lorlatinib. Lung Cancer.

[108] Longuespée R., Kunz J., Fresnais M., Foerster K.I., Burhenne J., Thomas M., Kazdal D., Stenzinger A., Christopoulos P., Haefeli W.E. (2024). Therapeutic drug monitoring of osimertinib in non-small cell lung cancer and short bowel syndrome: A case report. Br. J. Clin. Pharmacol..

[109] Chen J., Ruiz-Garcia A., James L.P., Peltz G., Thurm H., Clancy J., Hibma J. (2021). Lorlatinib Exposure-Response Analyses for Safety and Efficacy in a Phase I/II Trial to Support Benefit-Risk Assessment in Non-Small Cell Lung Cancer. Clin. Pharmacol. Ther..

[110] Igawa Y., Yoshida T., Makihara R., Torasawa M., Tateishi A., Matsumoto Y., Shinno Y., Okuma Y., Goto Y., Horinouchi H. (2024). Association between lorlatinib blood concentration and adverse events and clinical impact of dose modification. Lung Cancer.

[111] Cui L. (2024). Sample size adaptation designs and efficiency comparison with group sequential designs. Stat. Med..

[112] Lee A.T.M., Ou S.-H.I. (2024). Overcoming Central β-Sheet #6 (Cβ6) ALK Mutation (L1256F), TP53 Mutations and Short Forms of EML4-ALK v3/b and v5a/b Splice Variants are the Unmet Need That a Re-Imagined 5th-Generation (5G) ALK TKI Must Deliver. Lung Cancer Targets Ther..

[113] Christopoulos P., Budczies J., Wiesweg M., Eul B., Frost N., Misch D., Atmaca A., Schulz C., Sandner E., Heilmann M. (2025). 1980P Tumor mutational burden (TMB) and proliferation as negative prognostic factors for ALK+ lung cancer: First results from the randomized ABP phase II trial. Ann. Oncol..

[114] Hochhaus A., Talpaz M., Saglio G., Cortes J.E., Hughes T., Apperley J., Hantschel O., Rea D., Schuld P., Kantarjian H. (2026). From fatal disease to functional cure: 25 years of tyrosine kinase inh ibition in chronic myeloid leukemia. Leukemia.

[115] Lara-Mejía L., Cardona A.F., Mas L., Martin C., Samtani S., Corrales L., Cruz-Rico G., Remon J., Galvez-Nino M., Ruiz R. (2024). Impact of Concurrent Genomic Alterations on Clinical Outcomes in Patie nts With ALK-Rearranged NSCLC. J. Thorac. Oncol..

[116] Diaz-Jimenez A., Shuldiner E.G., Somogyi K., Shih K.Y., González-Velasco Ó., Najajreh M., Kim S., Akkas F., Murray C.W., Andrejka L. (2026). EML4−ALK Variant-Specific Genetic Interactions Shape Lung Tumorigenesis. Cancer Discov..

[117] Budczies J., Kazdal D., Menzel M., Beck S., Kluck K., Altbürger C., Schwab C., Allgäuer M., Ahadova A., Kloor M. (2024). Tumour mutational burden: Clinical utility, challenges and emerging improvements. Nat. Rev. Clin. Oncol..

[118] Budczies J., Kirchner M., Kluck K., Kazdal D., Glade J., Allgäuer M., Kriegsmann M., Heußel C.-P., Herth F.J., Winter H. (2021). Deciphering the immunosuppressive tumor microenvironment in ALK- and EGFR-positive lung adenocarcinoma. Cancer Immunol. Immunother..

[119] Mota I., Patrucco E., Mastini C., Mahadevan N.R., Thai T.C., Bergaggio E., Cheong T.-C., Leonardi G., Karaca-Atabay E., Campisi M. (2023). ALK peptide vaccination restores the immunogenicity of ALK-rearranged non-small cell lung cancer. Nat. Cancer.

[120] Leidner R., Silva N.S., Huang H., Sprott D., Zheng C., Shih Y.-P., Leung A., Payne R., Sutcliffe K., Cramer J. (2022). Neoantigen T-Cell Receptor Gene Therapy in Pancreatic Cancer. N. Engl. J. Med..

[121] Gaissmaier L., Elshiaty M., Christopoulos P. (2020). Breaking Bottlenecks for the TCR Therapy of Cancer. Cells.

[122] Team R.C. (2026). R: A Language and Environment for Statistical Computing.

[123] Wassmer G., Pahlke F. (2026). rpact: Confirmatory Adaptive Clinical Trial Design and Analysis.

[124] Lakatos E. (1988). Sample sizes based on the log-rank statistic in complex clinical trials. Biometrics.

[125] Schoenfeld D. (1981). The asymptotic properties of nonparametric tests for comparing survival distributions. Biometrika.

[126] Schoenfeld D.A. (1983). Sample-size formula for the proportional-hazards regression model. Biometrics.

[127] Jung S.H., Kang S.J., McCall L.M., Blumenstein B. (2005). Sample size computation for two-sample noninferiority log-rank test. J. Biopharm. Stat..

