# Supplementary material for: Current Management and the New Paradigm for ALK+ NSCLC After CROWN
Source: Cancers (Basel). 2026 Sep 19;18(18):3044. doi: 10.3390/cancers18183044 (PMC13605852; doi:10.3390/cancers18183044)
Supplement: Supplementary file 1 [file cancers-18-03044-s001.zip › File S1. Supplementary Material.pdf]

Supplementary Materials

1. Correction for the annual mortality of the general population

While the full classification of progression-free survival (PFS) events as progressive disease (PD) *vs.* disease-related or disease-unrelated deaths is not available, we can estimate the lower annual progression rate during the plateau phase after the 3rd year (with a stable annual PFS drop rate of 2.5%), as follows: among 7 PFS events between the CROWN 5- and 7-year analysis with an approximate PFS decrement of 5%, only 4 events were due to disease progression, while the remaining 3/7 represented unrelated deaths during disease remission [1]. This indicates an annual progression rate of approximately 1.4% (4/7x5%/2), *i.e.* approximately 1.1% lower than the average annual PFS drop rate (PFSDR) of 2.5% observed in years 6 and 7 (Table 2, column 3, unadjusted values), or 1.3%-1.4% lower than the adjusted annual PFSDR of 2.7-2.8% observed in years 6 and 7 (Table 2, column 3, adjusted values in parentheses). This difference is very similar to the age-specific mortality of 1.3% (1.8% for males, 0.9% for females) for the general population at the age of 65 years (*i.e.* probability of death at the age of 65 until the age of 66) in Europe and Northern America according to the most recent (year 2023) records of the United Nations, traceable in "estimates for both sexes" as q(x,n), year 2023, age 65, age interval 1 at https://population.un.org/wpp/downloads?folder=Standard%20Projections&group=Mortality, WPP2024, please select the appropriate region; NB. the respective global mortality is higher at 1.7% overall, 2.1% for males and 1.3% for females) [2]. If we additionally consider that the experimental arm of CROWN included 56% females and 44% males at baseline, the adjusted age-specific mortality for Europe and Northern America becomes exactly 1.3% (0.56*0.9 + 0.44*1.8 = 1.3).

The reference age of 65 corresponds to the median initial age of 61 years in the experimental arm of CROWN and a hypothetical average 4 years of follow-up until entry into the plateau, since the age of patients at risk by follow-up year is not available.

Therefore, and acknowledging the presence of some favorable features (e.g. 54% never smokers) as well as the approximate nature of the result, the annual rate of progression during the plateau phase under lorlatinib in CROWN, was estimated by subtracting a conservative 1.0% from the observed annual PFS decrement.

The estimated progression rate < 2% per year during the plateau phase adopted in this work, based on the empirically observed PFSDR of 2.5% per year after the 3rd year, is not sensitive to the exact value of the mortality correction. For example even a clearly underestimated correction by only 0.6% per year, less than half of the contemporary overall age-specific annual mortality in Western countries, would result in an estimated annual progression rate of 1.9%.

2. Calculation for the rate of late to early progression for ALK+ NSCLC under lorlatinib and selected precursor conditions

For lorlatinib, the ratio of progression events between years 4-7 and years 1-3 can be calculated as 11/49 events * 3/4 years, without the mortality correction, which would reduce the ratio further (Table 2).

For leukoplakia, the progression rate remains constant over at least 10 years per reference [3].

For smoldering myeloma, the ratio of progression events between years 6-10 and years 1-5 can be directly calculated as approximately 46% ((43.6-29.8)/29.8) based on the respective rates of cumulative progression from Figure 1C of reference [4]; the ratio between years 4-7 and 1-3 (symmetric to the CROWN ratio) can be estimated as approximately 56% (16/21.5*3/4) by analyzing the slope of the progression incidence curve shown in Figure 1A of the same reference [4]. The cumulative and incremental rates of progression per year extracted from the curve of Figure 1A are tabulated below:

Year	Estimated Cumulative Progression Rate	Annual Incremental Progression Rate / year
Year 1	~0.07 – 0.08 (7.5%)	~7.5%
Year 2	~0.13 – 0.14 (13.5%)	~6.0%
Year 3	~0.21 – 0.22 (21.5%)	~8.0%
Year 4	~0.25 – 0.26 (25.5%)	~4.0%
Year 5	~0.29 – 0.30 (29.5%)	~4.0%
Year 6	~0.34 – 0.35 (34.5%)	~5.0%
Year 7	~0.37 – 0.38 (37.5%)	~3.0%
Year 8	~0.39 – 0.40 (39.5%)	~2.0%
Year 9	~0.42 – 0.43 (42.5%)	~3.0%
Year 10	~0.43 – 0.44 (43.5%)	~1.0%

3. Approximation for the lorlatinib-to-neladalkib ratios for trough and intracranial free drug concentrations

For an approximate comparison, terminal elimination was modeled as monoexponential, with $C(t) = C_0 e^{\{-kt\}}$, $k = \frac{\ln(2)}{t_{1/2}}$, and a 24-hour dosing interval. Using steady-state maximum unbound plasma concentrations of 483 nM for lorlatinib and 40 nM for neladalkib, and terminal half-lives of 24 hours and 40–55 hours, respectively, as explained in the main text [5], the estimated trough concentrations immediately before the next dose were 241.5 nM for lorlatinib and 26.4–29.6 nM for neladalkib. The corresponding plasma trough concentration ratio was therefore 8.2–9.2-fold.

After adjustment for $K_{p,uu,brain}$ values of 0.11 and 0.16, respectively, using the formula $\text{Ratio}_{\text{brain}} = \text{Ratio}_{\text{plasma}} \times \frac{0.11}{0.16}$, the estimated corresponding intracranial free-drug concentration ratio was 5.6–6.3-fold, or approximately 6-fold.

4. The inadequacy of current clinical trial design methods withing the new paradigm of extraordinary drug efficacy

In order to detect **non-inferiority with a hazard ratio (HR) margin of 1.25 for a drug with equal potency (i.e. true HR = 1)** $vs.$ **lorlatinib under the simplifying assumptions of equal allocation, proportional hazards, no dropouts and a fixed-horizon survival analysis, 846 events are needed for a power of 90% with a one-sided alpha of 2.5%**. Assuming enrollment of 160 patients per year, similar to CROWN with 104 centers in 23 countries [6], at least 5.3 years of enrollment would be necessary. Below are some alternatives for the duration of enrollment and follow-up (FU) assuming a median PFS of 9 years for the control arm and an exponential PFS distribution:

a. 960 patients enrolled in 6 years → 24.8 years of FU after completion of enrollment for PFS maturity.

b. 1120 patients enrolled in 7 years → 14.9 years of FU after completion of enrollment for PFS maturity.

c. 1280 patients enrolled in 8 years → 10.2 years of FU after completion of enrollment for PFS maturity.

d. 1440 patients enrolled in 9 years → 7.3 years of FU after completion of enrollment for PFS maturity.

e. 1600 patients enrolled in 10 years → 5.1 years of FU after completion of enrollment for PFS maturity.

In order to detect **superiority of a novel drug with a true HR of 0.75 *vs.* lorlatinib under the simplifying assumptions of equal allocation, proportional hazards, no dropouts and a fixed-horizon survival analysis, 508 events are needed for a power of 90% with a two-sided alpha of 5%**. Assuming enrollment of 160 patients per year, similar to CROWN with 104 centers in 23 countries [6], at least 3.2 years of enrollment would be necessary. Below are some alternatives for the duration of enrollment and FU assuming a median PFS of 9 years for the control arm and an exponential PFS distribution:

a. 640 patients enrolled in 4 years → 21.9 years of FU after completion of enrollment for PFS maturity.

b. 800 patients enrolled in 5 years → 12.7 years of FU after completion of enrollment for PFS maturity.

c. 960 patients enrolled in 6 years → 8.4 years of FU after completion of enrollment for PFS maturity.

d. 1120 patients enrolled in 7 years → 5.7 years of FU after completion of enrollment for PFS maturity.

e. 1280 patients enrolled in 8 years → 3.7 years of FU after completion of enrollment for PFS maturity.

The calculations were performed with PASS 2024 v24.0.9 (NCSS, LLC, Kaysville, Utah, USA) and R v4.6.1 using the package rpact v4.4.0 [7,8]. The full output tables from PASS are provided as Supplementary Files 2 and 3. For superiority studies, PASS calculates the sample size using the Lakatos method [9], which, under our simplifying assumptions—proportional hazards and no dropouts—produces very similar results to the Schoenfeld formula [10,11]. The calculation for the non-inferiority study is based on a modification/extension by Jung *et al.* [12].

These calculations assume that PFS times follow an exponential distribution, corresponding to a constant hazard of progression or death over time. Replacing this assumption with the plateau-like PFS pattern observed for lorlatinib in CROWN (20% PFSDR in the first year, 10% in the second year, 5% in the third year, and 2.5% annually thereafter, according to Table 2) is not expected to materially change the number of events required for a given power, type I error rate, assumed treatment-effect HR or non-inferiority margin, and allocation ratio under the proportional-hazards framework, but would substantially prolong the calendar time required to reach event maturity.

References for the Supplementary materials

1. Shaw, A.T.; Solomon, B.J.; Felip, E.; Bauer, T.M.; Liu, G.; Goto, Y.; Wu, Y.L.; Soo, R.A.; Mazieres, J.; Kim, D.W.; et al. Lorlatinib versus crizotinib as first-line treatment for advanced ALK-positive non-small-cell lung cancer: 7-year update from the phase III CROWN study. *Ann. Oncol.* **2026**, S0923–7534(0926)00876–00878, doi:10.1016/j.annonc.2026.05.692.
2. United Nations, D.o.E.; Social Affairs, P.D. World Population Prospects 2024, Online Edition. *World Population Prospects 2024, Online Edition.*, Available online: https://population.un.org/wpp/downloads?folder=Standard%20Projections&group=Mortality (accessed on 29 August 2026).

3. Evren, I.; Brouns, E.R.; Wils, L.J.; Poell, J.B.; Peeters, C.F.W.; Brakenhoff, R.H.; Bloemena, E.; Visscher, J.G.A.M. Annual malignant transformation rate of oral leukoplakia remains consi tent: A long-term follow-up study. *Oral Oncol.* **2020**, *110*, 105014, doi:10.1016/j.oraloncology.2020.105014.
4. Akhlaghi, T.; Maclachlan, K.H.; Wiggins, A.; Usmani, S.Z.; Derkach, A.; Hultcrantz, M. Prognosis and patterns of progression in smoldering multiple myeloma. *Blood Advances* **2025**, *10*, 1344–1347, doi:10.1182/bloodadvances.2025018021.
5. Lin, J.J.; Horan, J.C.; Tangpeerachaikul, A.; Swalduz, A.; Valdivia, A.; Johnson, M.L.; Besse, B.; Camidge, D.R.; Fujino, T.; Yoda, S.; et al. NVL-655 Is a Selective and Brain-Penetrant Inhibitor of Diverse ALK-Mutant Oncoproteins, Including Lorlatinib-Resistant Compound Mutations. *Cancer Discov.* **2024**, *14*, 2367–2386, doi:10.1158/2159-8290.CD-24-0231.
6. Shaw, A.T.; Bauer, T.M.; Marinis, F.; Felip, E.; Goto, Y.; Liu, G.; Mazieres, J.; Kim, D.-W.; Mok, T.; Polli, A.; et al. First-Line Lorlatinib or Crizotinib in Advanced ALK-Positive Lung Canc r. *N. Engl. J. Med.* **2020**, *383*, 2018–2029, doi:10.1056/NEJMoa2027187.
7. Team, R.C. *R: A Language and Environment for Statistical Computing*, R Foundation for Statistical Computing: Vienna, Austria, 2026.
8. Wassmer, G.; Pahlke, F. *rpact: Confirmatory Adaptive Clinical Trial Design and Analysis*, R Foundation for Statistical Computing: 2026.
9. Lakatos, E. Sample sizes based on the log-rank statistic in complex clinical trials. *Biometrics* **1988**, *44*, 229–241.
10. Schoenfeld, D. The asymptotic properties of nonparametric tests for comparing survival distributions. *Biometrika* **1981**, *68*, 316–319, doi:10.1093/biomet/68.1.316.
11. Schoenfeld, D.A. Sample-size formula for the proportional-hazards regression model. *Biometrics* **1983**, *39*, 499–503.
12. Jung, S.H.; Kang, S.J.; McCall, L.M.; Blumenstein, B. Sample size computation for two-sample noninferiority log-rank test. *J. Biopharm. Stat.* **2005**, *15*, 969–979, doi:10.1080/10543400500265736.
