# Supplementary material for: Current Management and the New Paradigm for ALK+ NSCLC After CROWN
Source: Cancers (Basel). 2026 Sep 19;18(18):3044. doi: 10.3390/cancers18183044 (PMC13605852; doi:10.3390/cancers18183044)
Supplement: Supplementary file 1 [file cancers-18-03044-s001.zip › File S2. Noninferiority calculation.pdf]

Non-Inferiority Logrank Tests

Numeric Results (Sample Size)

Solve For: Power
Test Type: Two-Sample Logrank Test
Groups: 1 = Reference, 2 = Treatment
Hypotheses: H0: HR ≥ HR0 vs. H1: HR < HR0

	Sample Size			Hazard Ratio		Ref. Group Hazard Rate		Time		Proportion Lost*		Proportion Switching Groups*		
Power	N1	N2	N	Non-Inferiority HR0	Actual HR1	h1	Accrual Pattern	Accrual	Total	Ref.	Treat.	Ref. to Treat.	Treat. to Ref.	Alpha
0,7636	480	480	960	1,25	1	0,077	Equal	6	15	0	0	0	0	0,025
0,8242	560	560	1120	1,25	1	0,077	Equal	6	15	0	0	0	0	0,025
0,8708	640	640	1280	1,25	1	0,077	Equal	6	15	0	0	0	0	0,025
0,9061	720	720	1440	1,25	1	0,077	Equal	6	15	0	0	0	0	0,025
0,9323	800	800	1600	1,25	1	0,077	Equal	6	15	0	0	0	0	0,025
0,7832	480	480	960	1,25	1	0,077	Equal	6	16	0	0	0	0	0,025
0,8418	560	560	1120	1,25	1	0,077	Equal	6	16	0	0	0	0	0,025
0,8860	640	640	1280	1,25	1	0,077	Equal	6	16	0	0	0	0	0,025
0,9188	720	720	1440	1,25	1	0,077	Equal	6	16	0	0	0	0	0,025
0,9427	800	800	1600	1,25	1	0,077	Equal	6	16	0	0	0	0	0,025
0,8001	480	480	960	1,25	1	0,077	Equal	6	17	0	0	0	0	0,025
0,8567	560	560	1120	1,25	1	0,077	Equal	6	17	0	0	0	0	0,025
0,8986	640	640	1280	1,25	1	0,077	Equal	6	17	0	0	0	0	0,025
0,9291	720	720	1440	1,25	1	0,077	Equal	6	17	0	0	0	0	0,025
0,9509	800	800	1600	1,25	1	0,077	Equal	6	17	0	0	0	0	0,025
0,8147	480	480	960	1,25	1	0,077	Equal	6	18	0	0	0	0	0,025
0,8694	560	560	1120	1,25	1	0,077	Equal	6	18	0	0	0	0	0,025
0,9091	640	640	1280	1,25	1	0,077	Equal	6	18	0	0	0	0	0,025
0,9376	720	720	1440	1,25	1	0,077	Equal	6	18	0	0	0	0	0,025
0,9575	800	800	1600	1,25	1	0,077	Equal	6	18	0	0	0	0	0,025
0,8275	480	480	960	1,25	1	0,077	Equal	6	19	0	0	0	0	0,025
0,8802	560	560	1120	1,25	1	0,077	Equal	6	19	0	0	0	0	0,025
0,9180	640	640	1280	1,25	1	0,077	Equal	6	19	0	0	0	0	0,025
0,9446	720	720	1440	1,25	1	0,077	Equal	6	19	0	0	0	0	0,025
0,9629	800	800	1600	1,25	1	0,077	Equal	6	19	0	0	0	0	0,025
0,8386	480	480	960	1,25	1	0,077	Equal	6	20	0	0	0	0	0,025
0,8895	560	560	1120	1,25	1	0,077	Equal	6	20	0	0	0	0	0,025

Non-Inferiority Logrank Tests

Numeric Results (Sample Size) (Continued)

Solve For: Power
Test Type: Two-Sample Logrank Test
Groups: 1 = Reference, 2 = Treatment
Hypotheses: H0: HR ≥ HR0 vs. H1: HR < HR0

Power	Sample Size N1	Sample Size N2	Sample Size N	Hazard Ratio Non-Inferiority HR0	Hazard Ratio Actual HR1	Ref. Group Hazard Rate h1	Accrual Pattern	Time Accrual	Time Total	Proportion Lost* Ref.	Proportion Lost* Treat.	Proportion Switching Groups* Ref. to Treat.	Proportion Switching Groups* Treat. to Ref.	Alpha
0,9255	640	640	1280	1,25	1	0,077	Equal	6	20	0	0	0	0	0,025
0,9504	720	720	1440	1,25	1	0,077	Equal	6	20	0	0	0	0	0,025
0,9673	800	800	1600	1,25	1	0,077	Equal	6	20	0	0	0	0	0,025
0,8483	480	480	960	1,25	1	0,077	Equal	6	21	0	0	0	0	0,025
0,8975	560	560	1120	1,25	1	0,077	Equal	6	21	0	0	0	0	0,025
0,9318	640	640	1280	1,25	1	0,077	Equal	6	21	0	0	0	0	0,025
0,9553	720	720	1440	1,25	1	0,077	Equal	6	21	0	0	0	0	0,025
0,9710	800	800	1600	1,25	1	0,077	Equal	6	21	0	0	0	0	0,025
0,8568	480	480	960	1,25	1	0,077	Equal	6	22	0	0	0	0	0,025
0,9045	560	560	1120	1,25	1	0,077	Equal	6	22	0	0	0	0	0,025
0,9373	640	640	1280	1,25	1	0,077	Equal	6	22	0	0	0	0	0,025
0,9594	720	720	1440	1,25	1	0,077	Equal	6	22	0	0	0	0	0,025
0,9740	800	800	1600	1,25	1	0,077	Equal	6	22	0	0	0	0	0,025
0,8643	480	480	960	1,25	1	0,077	Equal	6	23	0	0	0	0	0,025
0,9105	560	560	1120	1,25	1	0,077	Equal	6	23	0	0	0	0	0,025
0,9419	640	640	1280	1,25	1	0,077	Equal	6	23	0	0	0	0	0,025
0,9629	720	720	1440	1,25	1	0,077	Equal	6	23	0	0	0	0	0,025
0,9765	800	800	1600	1,25	1	0,077	Equal	6	23	0	0	0	0	0,025
0,8709	480	480	960	1,25	1	0,077	Equal	6	24	0	0	0	0	0,025
0,9158	560	560	1120	1,25	1	0,077	Equal	6	24	0	0	0	0	0,025
0,9460	640	640	1280	1,25	1	0,077	Equal	6	24	0	0	0	0	0,025
0,9658	720	720	1440	1,25	1	0,077	Equal	6	24	0	0	0	0	0,025
0,9787	800	800	1600	1,25	1	0,077	Equal	6	24	0	0	0	0	0,025
0,8768	480	480	960	1,25	1	0,077	Equal	6	25	0	0	0	0	0,025
0,9205	560	560	1120	1,25	1	0,077	Equal	6	25	0	0	0	0	0,025
0,9495	640	640	1280	1,25	1	0,077	Equal	6	25	0	0	0	0	0,025
0,9684	720	720	1440	1,25	1	0,077	Equal	6	25	0	0	0	0	0,025

Non-Inferiority Logrank Tests

Numeric Results (Sample Size) (Continued)

Solve For: Power
Test Type: Two-Sample Logrank Test
Groups: 1 = Reference, 2 = Treatment
Hypotheses: H0: HR ≥ HR0 vs. H1: HR < HR0

	Sample Size			Hazard Ratio		Ref. Group Hazard Rate		Time		Proportion Lost*		Proportion Switching Groups*		
Power	N1	N2	N	Non-Inferiority HR0	Actual HR1	h1	Accrual Pattern	Accrual	Total	Ref.	Treat.	Ref. to Treat.	Treat. to Ref.	Alpha
0,9805	800	800	1600	1,25	1	0,077	Equal	6	25	0	0	0	0	0,025
0,8821	480	480	960	1,25	1	0,077	Equal	6	26	0	0	0	0	0,025
0,9246	560	560	1120	1,25	1	0,077	Equal	6	26	0	0	0	0	0,025
0,9526	640	640	1280	1,25	1	0,077	Equal	6	26	0	0	0	0	0,025
0,9706	720	720	1440	1,25	1	0,077	Equal	6	26	0	0	0	0	0,025
0,9820	800	800	1600	1,25	1	0,077	Equal	6	26	0	0	0	0	0,025
0,8867	480	480	960	1,25	1	0,077	Equal	6	27	0	0	0	0	0,025
0,9282	560	560	1120	1,25	1	0,077	Equal	6	27	0	0	0	0	0,025
0,9552	640	640	1280	1,25	1	0,077	Equal	6	27	0	0	0	0	0,025
0,9725	720	720	1440	1,25	1	0,077	Equal	6	27	0	0	0	0	0,025
0,9833	800	800	1600	1,25	1	0,077	Equal	6	27	0	0	0	0	0,025
0,8909	480	480	960	1,25	1	0,077	Equal	6	28	0	0	0	0	0,025
0,9314	560	560	1120	1,25	1	0,077	Equal	6	28	0	0	0	0	0,025
0,9576	640	640	1280	1,25	1	0,077	Equal	6	28	0	0	0	0	0,025
0,9742	720	720	1440	1,25	1	0,077	Equal	6	28	0	0	0	0	0,025
0,9845	800	800	1600	1,25	1	0,077	Equal	6	28	0	0	0	0	0,025
0,8947	480	480	960	1,25	1	0,077	Equal	6	29	0	0	0	0	0,025
0,9342	560	560	1120	1,25	1	0,077	Equal	6	29	0	0	0	0	0,025
0,9597	640	640	1280	1,25	1	0,077	Equal	6	29	0	0	0	0	0,025
0,9756	720	720	1440	1,25	1	0,077	Equal	6	29	0	0	0	0	0,025
0,9855	800	800	1600	1,25	1	0,077	Equal	6	29	0	0	0	0	0,025
0,8980	480	480	960	1,25	1	0,077	Equal	6	30	0	0	0	0	0,025
0,9368	560	560	1120	1,25	1	0,077	Equal	6	30	0	0	0	0	0,025
0,9615	640	640	1280	1,25	1	0,077	Equal	6	30	0	0	0	0	0,025
0,9769	720	720	1440	1,25	1	0,077	Equal	6	30	0	0	0	0	0,025
0,9863	800	800	1600	1,25	1	0,077	Equal	6	30	0	0	0	0	0,025
0,9010	480	480	960	1,25	1	0,077	Equal	6	31	0	0	0	0	0,025

Non-Inferiority Logrank Tests

Numeric Results (Sample Size) (Continued)

Solve For: Power
Test Type: Two-Sample Logrank Test
Groups: 1 = Reference, 2 = Treatment
Hypotheses: H0: HR ≥ HR0 vs. H1: HR < HR0

	Sample Size			Hazard Ratio		Ref. Group Hazard Rate	Accrual	Time		Proportion Lost*		Proportion Switching Groups*		
Power	N1	N2	N	Non-Inferiority HR0	Actual HR1	h1	Pattern	Accrual	Total	Ref.	Treat.	Ref. to Treat.	Treat. to Ref.	Alpha
0,9390	560	560	1120	1,25	1	0,077	Equal	6	31	0	0	0	0	0,025
0,9631	640	640	1280	1,25	1	0,077	Equal	6	31	0	0	0	0	0,025
0,9780	720	720	1440	1,25	1	0,077	Equal	6	31	0	0	0	0	0,025
0,9871	800	800	1600	1,25	1	0,077	Equal	6	31	0	0	0	0	0,025
0,7516	480	480	960	1,25	1	0,077	Equal	7	15	0	0	0	0	0,025
0,8133	560	560	1120	1,25	1	0,077	Equal	7	15	0	0	0	0	0,025
0,8613	640	640	1280	1,25	1	0,077	Equal	7	15	0	0	0	0	0,025
0,8980	720	720	1440	1,25	1	0,077	Equal	7	15	0	0	0	0	0,025
0,9257	800	800	1600	1,25	1	0,077	Equal	7	15	0	0	0	0	0,025
0,7729	480	480	960	1,25	1	0,077	Equal	7	16	0	0	0	0	0,025
0,8326	560	560	1120	1,25	1	0,077	Equal	7	16	0	0	0	0	0,025
0,8781	640	640	1280	1,25	1	0,077	Equal	7	16	0	0	0	0	0,025
0,9122	720	720	1440	1,25	1	0,077	Equal	7	16	0	0	0	0	0,025
0,9374	800	800	1600	1,25	1	0,077	Equal	7	16	0	0	0	0	0,025
0,7912	480	480	960	1,25	1	0,077	Equal	7	17	0	0	0	0	0,025
0,8489	560	560	1120	1,25	1	0,077	Equal	7	17	0	0	0	0	0,025
0,8921	640	640	1280	1,25	1	0,077	Equal	7	17	0	0	0	0	0,025
0,9237	720	720	1440	1,25	1	0,077	Equal	7	17	0	0	0	0	0,025
0,9467	800	800	1600	1,25	1	0,077	Equal	7	17	0	0	0	0	0,025
0,8071	480	480	960	1,25	1	0,077	Equal	7	18	0	0	0	0	0,025
0,8627	560	560	1120	1,25	1	0,077	Equal	7	18	0	0	0	0	0,025
0,9037	640	640	1280	1,25	1	0,077	Equal	7	18	0	0	0	0	0,025
0,9332	720	720	1440	1,25	1	0,077	Equal	7	18	0	0	0	0	0,025
0,9541	800	800	1600	1,25	1	0,077	Equal	7	18	0	0	0	0	0,025
0,8208	480	480	960	1,25	1	0,077	Equal	7	19	0	0	0	0	0,025
0,8745	560	560	1120	1,25	1	0,077	Equal	7	19	0	0	0	0	0,025
0,9134	640	640	1280	1,25	1	0,077	Equal	7	19	0	0	0	0	0,025

Non-Inferiority Logrank Tests

Numeric Results (Sample Size) (Continued)

Solve For: Power
Test Type: Two-Sample Logrank Test
Groups: 1 = Reference, 2 = Treatment
Hypotheses: H0: HR ≥ HR0 vs. H1: HR < HR0

	Sample Size			Hazard Ratio		Ref. Group Hazard Rate		Time		Proportion Lost*		Proportion Switching Groups*		
Power	N1	N2	N	Non-Inferiority HR0	Actual HR1	h1	Accrual Pattern	Accrual	Total	Ref.	Treat.	Ref. to Treat.	Treat. to Ref.	Alpha
0,9409	720	720	1440	1,25	1	0,077	Equal	7	19	0	0	0	0	0,025
0,9602	800	800	1600	1,25	1	0,077	Equal	7	19	0	0	0	0	0,025
0,8327	480	480	960	1,25	1	0,077	Equal	7	20	0	0	0	0	0,025
0,8846	560	560	1120	1,25	1	0,077	Equal	7	20	0	0	0	0	0,025
0,9216	640	640	1280	1,25	1	0,077	Equal	7	20	0	0	0	0	0,025
0,9474	720	720	1440	1,25	1	0,077	Equal	7	20	0	0	0	0	0,025
0,9651	800	800	1600	1,25	1	0,077	Equal	7	20	0	0	0	0	0,025
0,8432	480	480	960	1,25	1	0,077	Equal	7	21	0	0	0	0	0,025
0,8933	560	560	1120	1,25	1	0,077	Equal	7	21	0	0	0	0	0,025
0,9285	640	640	1280	1,25	1	0,077	Equal	7	21	0	0	0	0	0,025
0,9527	720	720	1440	1,25	1	0,077	Equal	7	21	0	0	0	0	0,025
0,9691	800	800	1600	1,25	1	0,077	Equal	7	21	0	0	0	0	0,025
0,8523	480	480	960	1,25	1	0,077	Equal	7	22	0	0	0	0	0,025
0,9008	560	560	1120	1,25	1	0,077	Equal	7	22	0	0	0	0	0,025
0,9344	640	640	1280	1,25	1	0,077	Equal	7	22	0	0	0	0	0,025
0,9572	720	720	1440	1,25	1	0,077	Equal	7	22	0	0	0	0	0,025
0,9724	800	800	1600	1,25	1	0,077	Equal	7	22	0	0	0	0	0,025
0,8604	480	480	960	1,25	1	0,077	Equal	7	23	0	0	0	0	0,025
0,9074	560	560	1120	1,25	1	0,077	Equal	7	23	0	0	0	0	0,025
0,9395	640	640	1280	1,25	1	0,077	Equal	7	23	0	0	0	0	0,025
0,9610	720	720	1440	1,25	1	0,077	Equal	7	23	0	0	0	0	0,025
0,9752	800	800	1600	1,25	1	0,077	Equal	7	23	0	0	0	0	0,025
0,8674	480	480	960	1,25	1	0,077	Equal	7	24	0	0	0	0	0,025
0,9130	560	560	1120	1,25	1	0,077	Equal	7	24	0	0	0	0	0,025
0,9439	640	640	1280	1,25	1	0,077	Equal	7	24	0	0	0	0	0,025
0,9643	720	720	1440	1,25	1	0,077	Equal	7	24	0	0	0	0	0,025
0,9775	800	800	1600	1,25	1	0,077	Equal	7	24	0	0	0	0	0,025

Non-Inferiority Logrank Tests

Numeric Results (Sample Size) (Continued)

Solve For: Power
Test Type: Two-Sample Logrank Test
Groups: 1 = Reference, 2 = Treatment
Hypotheses: H0: HR ≥ HR0 vs. H1: HR < HR0

	Sample Size			Hazard Ratio		Ref. Group Hazard Rate		Time		Proportion Lost*		Proportion Switching Groups*		
Power	N1	N2	N	Non-Inferiority HR0	Actual HR1	h1	Accrual Pattern	Accrual	Total	Ref.	Treat.	Ref. to Treat.	Treat. to Ref.	Alpha
0,8737	480	480	960	1,25	1	0,077	Equal	7	25	0	0	0	0	0,025
0,9180	560	560	1120	1,25	1	0,077	Equal	7	25	0	0	0	0	0,025
0,9477	640	640	1280	1,25	1	0,077	Equal	7	25	0	0	0	0	0,025
0,9671	720	720	1440	1,25	1	0,077	Equal	7	25	0	0	0	0	0,025
0,9795	800	800	1600	1,25	1	0,077	Equal	7	25	0	0	0	0	0,025
0,8793	480	480	960	1,25	1	0,077	Equal	7	26	0	0	0	0	0,025
0,9224	560	560	1120	1,25	1	0,077	Equal	7	26	0	0	0	0	0,025
0,9509	640	640	1280	1,25	1	0,077	Equal	7	26	0	0	0	0	0,025
0,9694	720	720	1440	1,25	1	0,077	Equal	7	26	0	0	0	0	0,025
0,9812	800	800	1600	1,25	1	0,077	Equal	7	26	0	0	0	0	0,025
0,8843	480	480	960	1,25	1	0,077	Equal	7	27	0	0	0	0	0,025
0,9263	560	560	1120	1,25	1	0,077	Equal	7	27	0	0	0	0	0,025
0,9538	640	640	1280	1,25	1	0,077	Equal	7	27	0	0	0	0	0,025
0,9715	720	720	1440	1,25	1	0,077	Equal	7	27	0	0	0	0	0,025
0,9826	800	800	1600	1,25	1	0,077	Equal	7	27	0	0	0	0	0,025
0,8887	480	480	960	1,25	1	0,077	Equal	7	28	0	0	0	0	0,025
0,9297	560	560	1120	1,25	1	0,077	Equal	7	28	0	0	0	0	0,025
0,9563	640	640	1280	1,25	1	0,077	Equal	7	28	0	0	0	0	0,025
0,9733	720	720	1440	1,25	1	0,077	Equal	7	28	0	0	0	0	0,025
0,9839	800	800	1600	1,25	1	0,077	Equal	7	28	0	0	0	0	0,025
0,8927	480	480	960	1,25	1	0,077	Equal	7	29	0	0	0	0	0,025
0,9327	560	560	1120	1,25	1	0,077	Equal	7	29	0	0	0	0	0,025
0,9586	640	640	1280	1,25	1	0,077	Equal	7	29	0	0	0	0	0,025
0,9749	720	720	1440	1,25	1	0,077	Equal	7	29	0	0	0	0	0,025
0,9850	800	800	1600	1,25	1	0,077	Equal	7	29	0	0	0	0	0,025
0,8962	480	480	960	1,25	1	0,077	Equal	7	30	0	0	0	0	0,025
0,9354	560	560	1120	1,25	1	0,077	Equal	7	30	0	0	0	0	0,025

Non-Inferiority Logrank Tests

Numeric Results (Sample Size) (Continued)

Solve For: Power
Test Type: Two-Sample Logrank Test
Groups: 1 = Reference, 2 = Treatment
Hypotheses: H0: HR ≥ HR0 vs. H1: HR < HR0

	Sample Size			Hazard Ratio		Ref. Group Hazard Rate	Accrual	Time		Proportion Lost*		Proportion Switching Groups*		
Power	N1	N2	N	Non-Inferiority HR0	Actual HR1	h1	Pattern	Accrual	Total	Ref.	Treat.	Ref. to Treat.	Treat. to Ref.	Alpha
0,9605	640	640	1280	1,25	1	0,077	Equal	7	30	0	0	0	0	0,025
0,9762	720	720	1440	1,25	1	0,077	Equal	7	30	0	0	0	0	0,025
0,9859	800	800	1600	1,25	1	0,077	Equal	7	30	0	0	0	0	0,025
0,8994	480	480	960	1,25	1	0,077	Equal	7	31	0	0	0	0	0,025
0,9378	560	560	1120	1,25	1	0,077	Equal	7	31	0	0	0	0	0,025
0,9623	640	640	1280	1,25	1	0,077	Equal	7	31	0	0	0	0	0,025
0,9774	720	720	1440	1,25	1	0,077	Equal	7	31	0	0	0	0	0,025
0,9867	800	800	1600	1,25	1	0,077	Equal	7	31	0	0	0	0	0,025
0,7385	480	480	960	1,25	1	0,077	Equal	8	15	0	0	0	0	0,025
0,8012	560	560	1120	1,25	1	0,077	Equal	8	15	0	0	0	0	0,025
0,8506	640	640	1280	1,25	1	0,077	Equal	8	15	0	0	0	0	0,025
0,8888	720	720	1440	1,25	1	0,077	Equal	8	15	0	0	0	0	0,025
0,9180	800	800	1600	1,25	1	0,077	Equal	8	15	0	0	0	0	0,025
0,7617	480	480	960	1,25	1	0,077	Equal	8	16	0	0	0	0	0,025
0,8225	560	560	1120	1,25	1	0,077	Equal	8	16	0	0	0	0	0,025
0,8693	640	640	1280	1,25	1	0,077	Equal	8	16	0	0	0	0	0,025
0,9048	720	720	1440	1,25	1	0,077	Equal	8	16	0	0	0	0	0,025
0,9313	800	800	1600	1,25	1	0,077	Equal	8	16	0	0	0	0	0,025
0,7815	480	480	960	1,25	1	0,077	Equal	8	17	0	0	0	0	0,025
0,8403	560	560	1120	1,25	1	0,077	Equal	8	17	0	0	0	0	0,025
0,8847	640	640	1280	1,25	1	0,077	Equal	8	17	0	0	0	0	0,025
0,9177	720	720	1440	1,25	1	0,077	Equal	8	17	0	0	0	0	0,025
0,9418	800	800	1600	1,25	1	0,077	Equal	8	17	0	0	0	0	0,025
0,7987	480	480	960	1,25	1	0,077	Equal	8	18	0	0	0	0	0,025
0,8554	560	560	1120	1,25	1	0,077	Equal	8	18	0	0	0	0	0,025
0,8976	640	640	1280	1,25	1	0,077	Equal	8	18	0	0	0	0	0,025
0,9282	720	720	1440	1,25	1	0,077	Equal	8	18	0	0	0	0	0,025

Non-Inferiority Logrank Tests

Numeric Results (Sample Size) (Continued)

Solve For: Power
Test Type: Two-Sample Logrank Test
Groups: 1 = Reference, 2 = Treatment
Hypotheses: H0: HR ≥ HR0 vs. H1: HR < HR0

	Sample Size			Hazard Ratio		Ref. Group Hazard Rate	Accrual	Time		Proportion Lost*		Proportion Switching Groups*		
Power	N1	N2	N	Non-Inferiority HR0	Actual HR1	h1	Pattern	Accrual	Total	Ref.	Treat.	Ref. to Treat.	Treat. to Ref.	Alpha
0,9503	800	800	1600	1,25	1	0,077	Equal	8	18	0	0	0	0	0,025
0,8135	480	480	960	1,25	1	0,077	Equal	8	19	0	0	0	0	0,025
0,8683	560	560	1120	1,25	1	0,077	Equal	8	19	0	0	0	0	0,025
0,9083	640	640	1280	1,25	1	0,077	Equal	8	19	0	0	0	0	0,025
0,9369	720	720	1440	1,25	1	0,077	Equal	8	19	0	0	0	0	0,025
0,9570	800	800	1600	1,25	1	0,077	Equal	8	19	0	0	0	0	0,025
0,8264	480	480	960	1,25	1	0,077	Equal	8	20	0	0	0	0	0,025
0,8793	560	560	1120	1,25	1	0,077	Equal	8	20	0	0	0	0	0,025
0,9172	640	640	1280	1,25	1	0,077	Equal	8	20	0	0	0	0	0,025
0,9440	720	720	1440	1,25	1	0,077	Equal	8	20	0	0	0	0	0,025
0,9625	800	800	1600	1,25	1	0,077	Equal	8	20	0	0	0	0	0,025
0,8376	480	480	960	1,25	1	0,077	Equal	8	21	0	0	0	0	0,025
0,8887	560	560	1120	1,25	1	0,077	Equal	8	21	0	0	0	0	0,025
0,9248	640	640	1280	1,25	1	0,077	Equal	8	21	0	0	0	0	0,025
0,9499	720	720	1440	1,25	1	0,077	Equal	8	21	0	0	0	0	0,025
0,9670	800	800	1600	1,25	1	0,077	Equal	8	21	0	0	0	0	0,025
0,8474	480	480	960	1,25	1	0,077	Equal	8	22	0	0	0	0	0,025
0,8968	560	560	1120	1,25	1	0,077	Equal	8	22	0	0	0	0	0,025
0,9313	640	640	1280	1,25	1	0,077	Equal	8	22	0	0	0	0	0,025
0,9548	720	720	1440	1,25	1	0,077	Equal	8	22	0	0	0	0	0,025
0,9707	800	800	1600	1,25	1	0,077	Equal	8	22	0	0	0	0	0,025
0,8561	480	480	960	1,25	1	0,077	Equal	8	23	0	0	0	0	0,025
0,9039	560	560	1120	1,25	1	0,077	Equal	8	23	0	0	0	0	0,025
0,9368	640	640	1280	1,25	1	0,077	Equal	8	23	0	0	0	0	0,025
0,9590	720	720	1440	1,25	1	0,077	Equal	8	23	0	0	0	0	0,025
0,9737	800	800	1600	1,25	1	0,077	Equal	8	23	0	0	0	0	0,025
0,8637	480	480	960	1,25	1	0,077	Equal	8	24	0	0	0	0	0,025

Non-Inferiority Logrank Tests

Numeric Results (Sample Size) (Continued)

Solve For: Power
Test Type: Two-Sample Logrank Test
Groups: 1 = Reference, 2 = Treatment
Hypotheses: H0: HR ≥ HR0 vs. H1: HR < HR0

	Sample Size			Hazard Ratio		Ref. Group Hazard Rate		Time		Proportion Lost*		Proportion Switching Groups*		
Power	N1	N2	N	Non-Inferiority HR0	Actual HR1	h1	Accrual Pattern	Accrual	Total	Ref.	Treat.	Ref. to Treat.	Treat. to Ref.	Alpha
0,9100	560	560	1120	1,25	1	0,077	Equal	8	24	0	0	0	0	0,025
0,9416	640	640	1280	1,25	1	0,077	Equal	8	24	0	0	0	0	0,025
0,9626	720	720	1440	1,25	1	0,077	Equal	8	24	0	0	0	0	0,025
0,9763	800	800	1600	1,25	1	0,077	Equal	8	24	0	0	0	0	0,025
0,8704	480	480	960	1,25	1	0,077	Equal	8	25	0	0	0	0	0,025
0,9154	560	560	1120	1,25	1	0,077	Equal	8	25	0	0	0	0	0,025
0,9457	640	640	1280	1,25	1	0,077	Equal	8	25	0	0	0	0	0,025
0,9656	720	720	1440	1,25	1	0,077	Equal	8	25	0	0	0	0	0,025
0,9785	800	800	1600	1,25	1	0,077	Equal	8	25	0	0	0	0	0,025
0,8763	480	480	960	1,25	1	0,077	Equal	8	26	0	0	0	0	0,025
0,9201	560	560	1120	1,25	1	0,077	Equal	8	26	0	0	0	0	0,025
0,9492	640	640	1280	1,25	1	0,077	Equal	8	26	0	0	0	0	0,025
0,9682	720	720	1440	1,25	1	0,077	Equal	8	26	0	0	0	0	0,025
0,9803	800	800	1600	1,25	1	0,077	Equal	8	26	0	0	0	0	0,025
0,8816	480	480	960	1,25	1	0,077	Equal	8	27	0	0	0	0	0,025
0,9242	560	560	1120	1,25	1	0,077	Equal	8	27	0	0	0	0	0,025
0,9523	640	640	1280	1,25	1	0,077	Equal	8	27	0	0	0	0	0,025
0,9704	720	720	1440	1,25	1	0,077	Equal	8	27	0	0	0	0	0,025
0,9819	800	800	1600	1,25	1	0,077	Equal	8	27	0	0	0	0	0,025
0,8863	480	480	960	1,25	1	0,077	Equal	8	28	0	0	0	0	0,025
0,9279	560	560	1120	1,25	1	0,077	Equal	8	28	0	0	0	0	0,025
0,9550	640	640	1280	1,25	1	0,077	Equal	8	28	0	0	0	0	0,025
0,9723	720	720	1440	1,25	1	0,077	Equal	8	28	0	0	0	0	0,025
0,9832	800	800	1600	1,25	1	0,077	Equal	8	28	0	0	0	0	0,025
0,8906	480	480	960	1,25	1	0,077	Equal	8	29	0	0	0	0	0,025
0,9311	560	560	1120	1,25	1	0,077	Equal	8	29	0	0	0	0	0,025
0,9574	640	640	1280	1,25	1	0,077	Equal	8	29	0	0	0	0	0,025

Non-Inferiority Logrank Tests

Numeric Results (Sample Size) (Continued)

Solve For: Power
Test Type: Two-Sample Logrank Test
Groups: 1 = Reference, 2 = Treatment
Hypotheses: H0: HR ≥ HR0 vs. H1: HR < HR0

	Sample Size			Hazard Ratio		Ref. Group Hazard Rate		Time		Proportion Lost*		Proportion Switching Groups*		
Power	N1	N2	N	Non-Inferiority HR0	Actual HR1	h1	Accrual Pattern	Accrual	Total	Ref.	Treat.	Ref. to Treat.	Treat. to Ref.	Alpha
0,9740	720	720	1440	1,25	1	0,077	Equal	8	29	0	0	0	0	0,025
0,9844	800	800	1600	1,25	1	0,077	Equal	8	29	0	0	0	0	0,025
0,8943	480	480	960	1,25	1	0,077	Equal	8	30	0	0	0	0	0,025
0,9340	560	560	1120	1,25	1	0,077	Equal	8	30	0	0	0	0	0,025
0,9595	640	640	1280	1,25	1	0,077	Equal	8	30	0	0	0	0	0,025
0,9755	720	720	1440	1,25	1	0,077	Equal	8	30	0	0	0	0	0,025
0,9854	800	800	1600	1,25	1	0,077	Equal	8	30	0	0	0	0	0,025
0,8977	480	480	960	1,25	1	0,077	Equal	8	31	0	0	0	0	0,025
0,9366	560	560	1120	1,25	1	0,077	Equal	8	31	0	0	0	0	0,025
0,9613	640	640	1280	1,25	1	0,077	Equal	8	31	0	0	0	0	0,025
0,9768	720	720	1440	1,25	1	0,077	Equal	8	31	0	0	0	0	0,025
0,9863	800	800	1600	1,25	1	0,077	Equal	8	31	0	0	0	0	0,025
0,7240	480	480	960	1,25	1	0,077	Equal	9	15	0	0	0	0	0,025
0,7877	560	560	1120	1,25	1	0,077	Equal	9	15	0	0	0	0	0,025
0,8385	640	640	1280	1,25	1	0,077	Equal	9	15	0	0	0	0	0,025
0,8783	720	720	1440	1,25	1	0,077	Equal	9	15	0	0	0	0	0,025
0,9090	800	800	1600	1,25	1	0,077	Equal	9	15	0	0	0	0	0,025
0,7493	480	480	960	1,25	1	0,077	Equal	9	16	0	0	0	0	0,025
0,8111	560	560	1120	1,25	1	0,077	Equal	9	16	0	0	0	0	0,025
0,8594	640	640	1280	1,25	1	0,077	Equal	9	16	0	0	0	0	0,025
0,8964	720	720	1440	1,25	1	0,077	Equal	9	16	0	0	0	0	0,025
0,9243	800	800	1600	1,25	1	0,077	Equal	9	16	0	0	0	0	0,025
0,7709	480	480	960	1,25	1	0,077	Equal	9	17	0	0	0	0	0,025
0,8308	560	560	1120	1,25	1	0,077	Equal	9	17	0	0	0	0	0,025
0,8765	640	640	1280	1,25	1	0,077	Equal	9	17	0	0	0	0	0,025
0,9109	720	720	1440	1,25	1	0,077	Equal	9	17	0	0	0	0	0,025
0,9363	800	800	1600	1,25	1	0,077	Equal	9	17	0	0	0	0	0,025

Non-Inferiority Logrank Tests

Numeric Results (Sample Size) (Continued)

Solve For: Power
Test Type: Two-Sample Logrank Test
Groups: 1 = Reference, 2 = Treatment
Hypotheses: H0: HR ≥ HR0 vs. H1: HR < HR0

	Sample Size			Hazard Ratio		Ref.		Time		Proportion Lost*		Proportion Switching Groups*		
Power	N1	N2	N	Non-Inferiority HR0	Actual HR1	Group Hazard Rate h1	Accrual Pattern	Accrual	Total	Ref.	Treat.	Ref. to Treat.	Treat. to Ref.	Alpha
0,7895	480	480	960	1,25	1	0,077	Equal	9	18	0	0	0	0	0,025
0,8474	560	560	1120	1,25	1	0,077	Equal	9	18	0	0	0	0	0,025
0,8907	640	640	1280	1,25	1	0,077	Equal	9	18	0	0	0	0	0,025
0,9227	720	720	1440	1,25	1	0,077	Equal	9	18	0	0	0	0	0,025
0,9458	800	800	1600	1,25	1	0,077	Equal	9	18	0	0	0	0	0,025
0,8056	480	480	960	1,25	1	0,077	Equal	9	19	0	0	0	0	0,025
0,8614	560	560	1120	1,25	1	0,077	Equal	9	19	0	0	0	0	0,025
0,9026	640	640	1280	1,25	1	0,077	Equal	9	19	0	0	0	0	0,025
0,9323	720	720	1440	1,25	1	0,077	Equal	9	19	0	0	0	0	0,025
0,9534	800	800	1600	1,25	1	0,077	Equal	9	19	0	0	0	0	0,025
0,8195	480	480	960	1,25	1	0,077	Equal	9	20	0	0	0	0	0,025
0,8734	560	560	1120	1,25	1	0,077	Equal	9	20	0	0	0	0	0,025
0,9125	640	640	1280	1,25	1	0,077	Equal	9	20	0	0	0	0	0,025
0,9402	720	720	1440	1,25	1	0,077	Equal	9	20	0	0	0	0	0,025
0,9596	800	800	1600	1,25	1	0,077	Equal	9	20	0	0	0	0	0,025
0,8316	480	480	960	1,25	1	0,077	Equal	9	21	0	0	0	0	0,025
0,8837	560	560	1120	1,25	1	0,077	Equal	9	21	0	0	0	0	0,025
0,9208	640	640	1280	1,25	1	0,077	Equal	9	21	0	0	0	0	0,025
0,9467	720	720	1440	1,25	1	0,077	Equal	9	21	0	0	0	0	0,025
0,9646	800	800	1600	1,25	1	0,077	Equal	9	21	0	0	0	0	0,025
0,8422	480	480	960	1,25	1	0,077	Equal	9	22	0	0	0	0	0,025
0,8925	560	560	1120	1,25	1	0,077	Equal	9	22	0	0	0	0	0,025
0,9278	640	640	1280	1,25	1	0,077	Equal	9	22	0	0	0	0	0,025
0,9522	720	720	1440	1,25	1	0,077	Equal	9	22	0	0	0	0	0,025
0,9687	800	800	1600	1,25	1	0,077	Equal	9	22	0	0	0	0	0,025
0,8514	480	480	960	1,25	1	0,077	Equal	9	23	0	0	0	0	0,025
0,9001	560	560	1120	1,25	1	0,077	Equal	9	23	0	0	0	0	0,025

Non-Inferiority Logrank Tests

Numeric Results (Sample Size) (Continued)

Solve For: Power
Test Type: Two-Sample Logrank Test
Groups: 1 = Reference, 2 = Treatment
Hypotheses: H0: HR ≥ HR0 vs. H1: HR < HR0

	Sample Size			Hazard Ratio		Ref. Group Hazard Rate		Time		Proportion Lost*		Proportion Switching Groups*		
Power	N1	N2	N	Non-Inferiority HR0	Actual HR1	h1	Accrual Pattern	Accrual	Total	Ref.	Treat.	Ref. to Treat.	Treat. to Ref.	Alpha
0,9339	640	640	1280	1,25	1	0,077	Equal	9	23	0	0	0	0	0,025
0,9568	720	720	1440	1,25	1	0,077	Equal	9	23	0	0	0	0	0,025
0,9721	800	800	1600	1,25	1	0,077	Equal	9	23	0	0	0	0	0,025
0,8596	480	480	960	1,25	1	0,077	Equal	9	24	0	0	0	0	0,025
0,9067	560	560	1120	1,25	1	0,077	Equal	9	24	0	0	0	0	0,025
0,9390	640	640	1280	1,25	1	0,077	Equal	9	24	0	0	0	0	0,025
0,9607	720	720	1440	1,25	1	0,077	Equal	9	24	0	0	0	0	0,025
0,9749	800	800	1600	1,25	1	0,077	Equal	9	24	0	0	0	0	0,025
0,8668	480	480	960	1,25	1	0,077	Equal	9	25	0	0	0	0	0,025
0,9125	560	560	1120	1,25	1	0,077	Equal	9	25	0	0	0	0	0,025
0,9435	640	640	1280	1,25	1	0,077	Equal	9	25	0	0	0	0	0,025
0,9640	720	720	1440	1,25	1	0,077	Equal	9	25	0	0	0	0	0,025
0,9773	800	800	1600	1,25	1	0,077	Equal	9	25	0	0	0	0	0,025
0,8731	480	480	960	1,25	1	0,077	Equal	9	26	0	0	0	0	0,025
0,9176	560	560	1120	1,25	1	0,077	Equal	9	26	0	0	0	0	0,025
0,9473	640	640	1280	1,25	1	0,077	Equal	9	26	0	0	0	0	0,025
0,9668	720	720	1440	1,25	1	0,077	Equal	9	26	0	0	0	0	0,025
0,9793	800	800	1600	1,25	1	0,077	Equal	9	26	0	0	0	0	0,025
0,8788	480	480	960	1,25	1	0,077	Equal	9	27	0	0	0	0	0,025
0,9220	560	560	1120	1,25	1	0,077	Equal	9	27	0	0	0	0	0,025
0,9506	640	640	1280	1,25	1	0,077	Equal	9	27	0	0	0	0	0,025
0,9692	720	720	1440	1,25	1	0,077	Equal	9	27	0	0	0	0	0,025
0,9810	800	800	1600	1,25	1	0,077	Equal	9	27	0	0	0	0	0,025
0,8838	480	480	960	1,25	1	0,077	Equal	9	28	0	0	0	0	0,025
0,9259	560	560	1120	1,25	1	0,077	Equal	9	28	0	0	0	0	0,025
0,9535	640	640	1280	1,25	1	0,077	Equal	9	28	0	0	0	0	0,025
0,9713	720	720	1440	1,25	1	0,077	Equal	9	28	0	0	0	0	0,025

Non-Inferiority Logrank Tests

Numeric Results (Sample Size) (Continued)

Solve For: Power
Test Type: Two-Sample Logrank Test
Groups: 1 = Reference, 2 = Treatment
Hypotheses: H0: HR ≥ HR0 vs. H1: HR < HR0

	Sample Size			Hazard Ratio		Ref. Group Hazard Rate		Time		Proportion Lost*		Proportion Switching Groups*		
Power	N1	N2	N	Non-Inferiority HR0	Actual HR1	h1	Accrual Pattern	Accrual	Total	Ref.	Treat.	Ref. to Treat.	Treat. to Ref.	Alpha
0,9825	800	800	1600	1,25	1	0,077	Equal	9	28	0	0	0	0	0,025
0,8883	480	480	960	1,25	1	0,077	Equal	9	29	0	0	0	0	0,025
0,9294	560	560	1120	1,25	1	0,077	Equal	9	29	0	0	0	0	0,025
0,9561	640	640	1280	1,25	1	0,077	Equal	9	29	0	0	0	0	0,025
0,9731	720	720	1440	1,25	1	0,077	Equal	9	29	0	0	0	0	0,025
0,9838	800	800	1600	1,25	1	0,077	Equal	9	29	0	0	0	0	0,025
0,8923	480	480	960	1,25	1	0,077	Equal	9	30	0	0	0	0	0,025
0,9324	560	560	1120	1,25	1	0,077	Equal	9	30	0	0	0	0	0,025
0,9583	640	640	1280	1,25	1	0,077	Equal	9	30	0	0	0	0	0,025
0,9747	720	720	1440	1,25	1	0,077	Equal	9	30	0	0	0	0	0,025
0,9848	800	800	1600	1,25	1	0,077	Equal	9	30	0	0	0	0	0,025
0,8959	480	480	960	1,25	1	0,077	Equal	9	31	0	0	0	0	0,025
0,9352	560	560	1120	1,25	1	0,077	Equal	9	31	0	0	0	0	0,025
0,9603	640	640	1280	1,25	1	0,077	Equal	9	31	0	0	0	0	0,025
0,9761	720	720	1440	1,25	1	0,077	Equal	9	31	0	0	0	0	0,025
0,9858	800	800	1600	1,25	1	0,077	Equal	9	31	0	0	0	0	0,025
0,7081	480	480	960	1,25	1	0,077	Equal	10	15	0	0	0	0	0,025
0,7727	560	560	1120	1,25	1	0,077	Equal	10	15	0	0	0	0	0,025
0,8248	640	640	1280	1,25	1	0,077	Equal	10	15	0	0	0	0	0,025
0,8662	720	720	1440	1,25	1	0,077	Equal	10	15	0	0	0	0	0,025
0,8987	800	800	1600	1,25	1	0,077	Equal	10	15	0	0	0	0	0,025
0,7356	480	480	960	1,25	1	0,077	Equal	10	16	0	0	0	0	0,025
0,7985	560	560	1120	1,25	1	0,077	Equal	10	16	0	0	0	0	0,025
0,8482	640	640	1280	1,25	1	0,077	Equal	10	16	0	0	0	0	0,025
0,8867	720	720	1440	1,25	1	0,077	Equal	10	16	0	0	0	0	0,025
0,9162	800	800	1600	1,25	1	0,077	Equal	10	16	0	0	0	0	0,025
0,7592	480	480	960	1,25	1	0,077	Equal	10	17	0	0	0	0	0,025

Non-Inferiority Logrank Tests

Numeric Results (Sample Size) (Continued)

Solve For: Power
Test Type: Two-Sample Logrank Test
Groups: 1 = Reference, 2 = Treatment
Hypotheses: H0: HR ≥ HR0 vs. H1: HR < HR0

	Sample Size			Hazard Ratio		Ref. Group Hazard Rate	Accrual	Time		Proportion Lost*		Proportion Switching Groups*		
Power	N1	N2	N	Non-Inferiority HR0	Actual HR1	h1	Pattern	Accrual	Total	Ref.	Treat.	Ref. to Treat.	Treat. to Ref.	Alpha
0,8202	560	560	1120	1,25	1	0,077	Equal	10	17	0	0	0	0	0,025
0,8673	640	640	1280	1,25	1	0,077	Equal	10	17	0	0	0	0	0,025
0,9031	720	720	1440	1,25	1	0,077	Equal	10	17	0	0	0	0	0,025
0,9299	800	800	1600	1,25	1	0,077	Equal	10	17	0	0	0	0	0,025
0,7794	480	480	960	1,25	1	0,077	Equal	10	18	0	0	0	0	0,025
0,8384	560	560	1120	1,25	1	0,077	Equal	10	18	0	0	0	0	0,025
0,8831	640	640	1280	1,25	1	0,077	Equal	10	18	0	0	0	0	0,025
0,9164	720	720	1440	1,25	1	0,077	Equal	10	18	0	0	0	0	0,025
0,9408	800	800	1600	1,25	1	0,077	Equal	10	18	0	0	0	0	0,025
0,7968	480	480	960	1,25	1	0,077	Equal	10	19	0	0	0	0	0,025
0,8538	560	560	1120	1,25	1	0,077	Equal	10	19	0	0	0	0	0,025
0,8962	640	640	1280	1,25	1	0,077	Equal	10	19	0	0	0	0	0,025
0,9271	720	720	1440	1,25	1	0,077	Equal	10	19	0	0	0	0	0,025
0,9494	800	800	1600	1,25	1	0,077	Equal	10	19	0	0	0	0	0,025
0,8119	480	480	960	1,25	1	0,077	Equal	10	20	0	0	0	0	0,025
0,8669	560	560	1120	1,25	1	0,077	Equal	10	20	0	0	0	0	0,025
0,9071	640	640	1280	1,25	1	0,077	Equal	10	20	0	0	0	0	0,025
0,9360	720	720	1440	1,25	1	0,077	Equal	10	20	0	0	0	0	0,025
0,9563	800	800	1600	1,25	1	0,077	Equal	10	20	0	0	0	0	0,025
0,8250	480	480	960	1,25	1	0,077	Equal	10	21	0	0	0	0	0,025
0,8781	560	560	1120	1,25	1	0,077	Equal	10	21	0	0	0	0	0,025
0,9163	640	640	1280	1,25	1	0,077	Equal	10	21	0	0	0	0	0,025
0,9432	720	720	1440	1,25	1	0,077	Equal	10	21	0	0	0	0	0,025
0,9619	800	800	1600	1,25	1	0,077	Equal	10	21	0	0	0	0	0,025
0,8364	480	480	960	1,25	1	0,077	Equal	10	22	0	0	0	0	0,025
0,8877	560	560	1120	1,25	1	0,077	Equal	10	22	0	0	0	0	0,025
0,9240	640	640	1280	1,25	1	0,077	Equal	10	22	0	0	0	0	0,025

Non-Inferiority Logrank Tests

Numeric Results (Sample Size) (Continued)

Solve For: Power
Test Type: Two-Sample Logrank Test
Groups: 1 = Reference, 2 = Treatment
Hypotheses: H0: HR ≥ HR0 vs. H1: HR < HR0

	Sample Size			Hazard Ratio		Ref. Group Hazard Rate		Time		Proportion Lost*		Proportion Switching Groups*		
Power	N1	N2	N	Non-Inferiority HR0	Actual HR1	h1	Accrual Pattern	Accrual	Total	Ref.	Treat.	Ref. to Treat.	Treat. to Ref.	Alpha
0,9493	720	720	1440	1,25	1	0,077	Equal	10	22	0	0	0	0	0,025
0,9665	800	800	1600	1,25	1	0,077	Equal	10	22	0	0	0	0	0,025
0,8464	480	480	960	1,25	1	0,077	Equal	10	23	0	0	0	0	0,025
0,8960	560	560	1120	1,25	1	0,077	Equal	10	23	0	0	0	0	0,025
0,9306	640	640	1280	1,25	1	0,077	Equal	10	23	0	0	0	0	0,025
0,9543	720	720	1440	1,25	1	0,077	Equal	10	23	0	0	0	0	0,025
0,9703	800	800	1600	1,25	1	0,077	Equal	10	23	0	0	0	0	0,025
0,8551	480	480	960	1,25	1	0,077	Equal	10	24	0	0	0	0	0,025
0,9031	560	560	1120	1,25	1	0,077	Equal	10	24	0	0	0	0	0,025
0,9362	640	640	1280	1,25	1	0,077	Equal	10	24	0	0	0	0	0,025
0,9586	720	720	1440	1,25	1	0,077	Equal	10	24	0	0	0	0	0,025
0,9734	800	800	1600	1,25	1	0,077	Equal	10	24	0	0	0	0	0,025
0,8628	480	480	960	1,25	1	0,077	Equal	10	25	0	0	0	0	0,025
0,9094	560	560	1120	1,25	1	0,077	Equal	10	25	0	0	0	0	0,025
0,9411	640	640	1280	1,25	1	0,077	Equal	10	25	0	0	0	0	0,025
0,9622	720	720	1440	1,25	1	0,077	Equal	10	25	0	0	0	0	0,025
0,9760	800	800	1600	1,25	1	0,077	Equal	10	25	0	0	0	0	0,025
0,8697	480	480	960	1,25	1	0,077	Equal	10	26	0	0	0	0	0,025
0,9148	560	560	1120	1,25	1	0,077	Equal	10	26	0	0	0	0	0,025
0,9452	640	640	1280	1,25	1	0,077	Equal	10	26	0	0	0	0	0,025
0,9653	720	720	1440	1,25	1	0,077	Equal	10	26	0	0	0	0	0,025
0,9782	800	800	1600	1,25	1	0,077	Equal	10	26	0	0	0	0	0,025
0,8757	480	480	960	1,25	1	0,077	Equal	10	27	0	0	0	0	0,025
0,9196	560	560	1120	1,25	1	0,077	Equal	10	27	0	0	0	0	0,025
0,9488	640	640	1280	1,25	1	0,077	Equal	10	27	0	0	0	0	0,025
0,9679	720	720	1440	1,25	1	0,077	Equal	10	27	0	0	0	0	0,025
0,9801	800	800	1600	1,25	1	0,077	Equal	10	27	0	0	0	0	0,025

Non-Inferiority Logrank Tests

Numeric Results (Sample Size) (Continued)

Solve For: Power
Test Type: Two-Sample Logrank Test
Groups: 1 = Reference, 2 = Treatment
Hypotheses: H0: HR ≥ HR0 vs. H1: HR < HR0

	Sample Size			Hazard Ratio				Time		Proportion Lost*		Proportion Switching Groups*		
Power	N1	N2	N	Non-Inferiority HR0	Actual HR1	Ref. Group Hazard Rate h1	Accrual Pattern	Accrual	Total	Ref.	Treat.	Ref. to Treat.	Treat. to Ref.	Alpha
0,8811	480	480	960	1,25	1	0,077	Equal	10	28	0	0	0	0	0,025
0,9238	560	560	1120	1,25	1	0,077	Equal	10	28	0	0	0	0	0,025
0,9520	640	640	1280	1,25	1	0,077	Equal	10	28	0	0	0	0	0,025
0,9702	720	720	1440	1,25	1	0,077	Equal	10	28	0	0	0	0	0,025
0,9817	800	800	1600	1,25	1	0,077	Equal	10	28	0	0	0	0	0,025
0,8858	480	480	960	1,25	1	0,077	Equal	10	29	0	0	0	0	0,025
0,9275	560	560	1120	1,25	1	0,077	Equal	10	29	0	0	0	0	0,025
0,9547	640	640	1280	1,25	1	0,077	Equal	10	29	0	0	0	0	0,025
0,9721	720	720	1440	1,25	1	0,077	Equal	10	29	0	0	0	0	0,025
0,9831	800	800	1600	1,25	1	0,077	Equal	10	29	0	0	0	0	0,025
0,8901	480	480	960	1,25	1	0,077	Equal	10	30	0	0	0	0	0,025
0,9308	560	560	1120	1,25	1	0,077	Equal	10	30	0	0	0	0	0,025
0,9571	640	640	1280	1,25	1	0,077	Equal	10	30	0	0	0	0	0,025
0,9738	720	720	1440	1,25	1	0,077	Equal	10	30	0	0	0	0	0,025
0,9843	800	800	1600	1,25	1	0,077	Equal	10	30	0	0	0	0	0,025
0,8939	480	480	960	1,25	1	0,077	Equal	10	31	0	0	0	0	0,025
0,9337	560	560	1120	1,25	1	0,077	Equal	10	31	0	0	0	0	0,025
0,9592	640	640	1280	1,25	1	0,077	Equal	10	31	0	0	0	0	0,025
0,9753	720	720	1440	1,25	1	0,077	Equal	10	31	0	0	0	0	0,025
0,9853	800	800	1600	1,25	1	0,077	Equal	10	31	0	0	0	0	0,025

* The reported proportions are during a single time period.

Non-Inferiority Logrank Tests

Power	The probability of rejecting a false null hypothesis when the alternative hypothesis is true.
N1, N2, N	The sample sizes of the reference group, treatment group, and both groups, respectively.
HR0	The Non-Inferiority Hazard Ratio is the upper bound for the hazard ratio that still leads to the conclusion of non-inferiority.
HR1	The Actual Hazard Ratio is assumed to be the actual value of the hazard ratio. This is always set to 1.
h1	The Reference Group Hazard Rate is the hazard (instantaneous failure) rate of the reference group. Its scale is events per time period.
Accrual Pattern	The pattern of accrual used for each time period.
Accrual Time	The number of time periods (years or months) during which accrual takes place.
Total Time	The total number of time periods in the study. Follow-up time = (Total Time) - (Accrual Time).
Reference Proportion Lost	The proportion of the reference group that is lost (drops out) during a single time period (e.g., year or month).
Treatment Proportion Lost	The proportion of the treatment group that is lost (drops out) during a single time period (e.g., year or month).
Reference to Treatment	Drop-In. The proportion of the reference group that switch to a group with a hazard rate equal to the treatment group.
Treatment to Reference	Noncompliance. The proportion of the treatment group that switch to a group with a hazard rate equal to the reference group.
Alpha	The probability of rejecting a true null hypothesis.

Non-Inferiority Logrank Tests

Numeric Results (Events)

Solve For: Power
Test Type: Two-Sample Logrank Test
Groups: 1 = Reference, 2 = Treatment
Hypotheses: H0: HR ≥ HR0 vs. H1: HR < HR0

	Number of Events			Hazard Ratio		Ref. Group Hazard Rate		Time		Proportion Lost*		Proportion Switching Groups*		
Power	E1	E2	E	Non-Inferiority HR0	Actual HR1	h1	Accrual Pattern	Accrual	Total	Ref.	Treat.	Ref. to Treat.	Treat. to Ref.	Alpha
0,7636	287,8	287,8	575,6	1,25	1	0,077	Equal	10	31	0	0	0	0	0,025
0,8242	335,7	335,7	671,5	1,25	1	0,077	Equal	10	31	0	0	0	0	0,025
0,8708	383,7	383,7	767,4	1,25	1	0,077	Equal	10	31	0	0	0	0	0,025
0,9061	431,7	431,7	863,3	1,25	1	0,077	Equal	10	31	0	0	0	0	0,025
0,9323	479,6	479,6	959,3	1,25	1	0,077	Equal	10	31	0	0	0	0	0,025
0,7832	302,0	302,0	604,0	1,25	1	0,077	Equal	10	31	0	0	0	0	0,025
0,8418	352,4	352,4	704,7	1,25	1	0,077	Equal	10	31	0	0	0	0	0,025
0,8860	402,7	402,7	805,4	1,25	1	0,077	Equal	10	31	0	0	0	0	0,025
0,9188	453,0	453,0	906,1	1,25	1	0,077	Equal	10	31	0	0	0	0	0,025
0,9427	503,4	503,4	1006,7	1,25	1	0,077	Equal	10	31	0	0	0	0	0,025
0,8001	315,2	315,2	630,4	1,25	1	0,077	Equal	10	31	0	0	0	0	0,025
0,8567	367,8	367,8	735,5	1,25	1	0,077	Equal	10	31	0	0	0	0	0,025
0,8986	420,3	420,3	840,6	1,25	1	0,077	Equal	10	31	0	0	0	0	0,025
0,9291	472,8	472,8	945,6	1,25	1	0,077	Equal	10	31	0	0	0	0	0,025
0,9509	525,4	525,4	1050,7	1,25	1	0,077	Equal	10	31	0	0	0	0	0,025
0,8147	327,4	327,4	654,9	1,25	1	0,077	Equal	10	31	0	0	0	0	0,025
0,8694	382,0	382,0	764,0	1,25	1	0,077	Equal	10	31	0	0	0	0	0,025
0,9091	436,6	436,6	873,1	1,25	1	0,077	Equal	10	31	0	0	0	0	0,025
0,9376	491,1	491,1	982,3	1,25	1	0,077	Equal	10	31	0	0	0	0	0,025
0,9575	545,7	545,7	1091,4	1,25	1	0,077	Equal	10	31	0	0	0	0	0,025
0,8275	338,7	338,7	677,5	1,25	1	0,077	Equal	10	31	0	0	0	0	0,025
0,8802	395,2	395,2	790,4	1,25	1	0,077	Equal	10	31	0	0	0	0	0,025
0,9180	451,6	451,6	903,3	1,25	1	0,077	Equal	10	31	0	0	0	0	0,025
0,9446	508,1	508,1	1016,2	1,25	1	0,077	Equal	10	31	0	0	0	0	0,025
0,9629	564,6	564,6	1129,1	1,25	1	0,077	Equal	10	31	0	0	0	0	0,025
0,8386	349,2	349,2	698,4	1,25	1	0,077	Equal	10	31	0	0	0	0	0,025
0,8895	407,4	407,4	814,8	1,25	1	0,077	Equal	10	31	0	0	0	0	0,025

Non-Inferiority Logrank Tests

Numeric Results (Events) (Continued)

Solve For: Power
Test Type: Two-Sample Logrank Test
Groups: 1 = Reference, 2 = Treatment
Hypotheses: H0: HR ≥ HR0 vs. H1: HR < HR0

	Number of Events			Hazard Ratio		Ref. Group Hazard Rate		Time		Proportion Lost*		Proportion Switching Groups*		
Power	E1	E2	E	Non-Inferiority HR0	Actual HR1	h1	Accrual Pattern	Accrual	Total	Ref.	Treat.	Ref. to Treat.	Treat. to Ref.	Alpha
0,9255	465,6	465,6	931,2	1,25	1	0,077	Equal	10	31	0	0	0	0	0,025
0,9504	523,8	523,8	1047,6	1,25	1	0,077	Equal	10	31	0	0	0	0	0,025
0,9673	582,0	582,0	1164,0	1,25	1	0,077	Equal	10	31	0	0	0	0	0,025
0,8483	358,9	358,9	717,8	1,25	1	0,077	Equal	10	31	0	0	0	0	0,025
0,8975	418,7	418,7	837,4	1,25	1	0,077	Equal	10	31	0	0	0	0	0,025
0,9318	478,5	478,5	957,1	1,25	1	0,077	Equal	10	31	0	0	0	0	0,025
0,9553	538,3	538,3	1076,7	1,25	1	0,077	Equal	10	31	0	0	0	0	0,025
0,9710	598,2	598,2	1196,3	1,25	1	0,077	Equal	10	31	0	0	0	0	0,025
0,8568	367,9	367,9	735,7	1,25	1	0,077	Equal	10	31	0	0	0	0	0,025
0,9045	429,2	429,2	858,4	1,25	1	0,077	Equal	10	31	0	0	0	0	0,025
0,9373	490,5	490,5	981,0	1,25	1	0,077	Equal	10	31	0	0	0	0	0,025
0,9594	551,8	551,8	1103,6	1,25	1	0,077	Equal	10	31	0	0	0	0	0,025
0,9740	613,1	613,1	1226,2	1,25	1	0,077	Equal	10	31	0	0	0	0	0,025
0,8643	376,2	376,2	752,4	1,25	1	0,077	Equal	10	31	0	0	0	0	0,025
0,9105	438,9	438,9	877,8	1,25	1	0,077	Equal	10	31	0	0	0	0	0,025
0,9419	501,6	501,6	1003,2	1,25	1	0,077	Equal	10	31	0	0	0	0	0,025
0,9629	564,3	564,3	1128,5	1,25	1	0,077	Equal	10	31	0	0	0	0	0,025
0,9765	627,0	627,0	1253,9	1,25	1	0,077	Equal	10	31	0	0	0	0	0,025
0,8709	383,9	383,9	767,8	1,25	1	0,077	Equal	10	31	0	0	0	0	0,025
0,9158	447,9	447,9	895,7	1,25	1	0,077	Equal	10	31	0	0	0	0	0,025
0,9460	511,8	511,8	1023,7	1,25	1	0,077	Equal	10	31	0	0	0	0	0,025
0,9658	575,8	575,8	1151,6	1,25	1	0,077	Equal	10	31	0	0	0	0	0,025
0,9787	639,8	639,8	1279,6	1,25	1	0,077	Equal	10	31	0	0	0	0	0,025
0,8768	391,0	391,0	782,0	1,25	1	0,077	Equal	10	31	0	0	0	0	0,025
0,9205	456,2	456,2	912,3	1,25	1	0,077	Equal	10	31	0	0	0	0	0,025
0,9495	521,3	521,3	1042,7	1,25	1	0,077	Equal	10	31	0	0	0	0	0,025
0,9684	586,5	586,5	1173,0	1,25	1	0,077	Equal	10	31	0	0	0	0	0,025

Non-Inferiority Logrank Tests

Numeric Results (Events) (Continued)

Solve For: Power
Test Type: Two-Sample Logrank Test
Groups: 1 = Reference, 2 = Treatment
Hypotheses: H0: HR ≥ HR0 vs. H1: HR < HR0

	Number of Events			Hazard Ratio		Ref. Group Hazard Rate	Accrual	Time		Proportion Lost*		Proportion Switching Groups*		
Power	E1	E2	E	Non-Inferiority HR0	Actual HR1	h1	Pattern	Accrual	Total	Ref.	Treat.	Ref. to Treat.	Treat. to Ref.	Alpha
0,9805	651,7	651,7	1303,3	1,25	1	0,077	Equal	10	31	0	0	0	0	0,025
0,8821	397,6	397,6	795,2	1,25	1	0,077	Equal	10	31	0	0	0	0	0,025
0,9246	463,9	463,9	927,7	1,25	1	0,077	Equal	10	31	0	0	0	0	0,025
0,9526	530,1	530,1	1060,3	1,25	1	0,077	Equal	10	31	0	0	0	0	0,025
0,9706	596,4	596,4	1192,8	1,25	1	0,077	Equal	10	31	0	0	0	0	0,025
0,9820	662,7	662,7	1325,3	1,25	1	0,077	Equal	10	31	0	0	0	0	0,025
0,8867	403,7	403,7	807,4	1,25	1	0,077	Equal	10	31	0	0	0	0	0,025
0,9282	471,0	471,0	942,0	1,25	1	0,077	Equal	10	31	0	0	0	0	0,025
0,9552	538,3	538,3	1076,5	1,25	1	0,077	Equal	10	31	0	0	0	0	0,025
0,9725	605,6	605,6	1211,1	1,25	1	0,077	Equal	10	31	0	0	0	0	0,025
0,9833	672,8	672,8	1345,7	1,25	1	0,077	Equal	10	31	0	0	0	0	0,025
0,8909	409,4	409,4	818,7	1,25	1	0,077	Equal	10	31	0	0	0	0	0,025
0,9314	477,6	477,6	955,2	1,25	1	0,077	Equal	10	31	0	0	0	0	0,025
0,9576	545,8	545,8	1091,6	1,25	1	0,077	Equal	10	31	0	0	0	0	0,025
0,9742	614,0	614,0	1228,1	1,25	1	0,077	Equal	10	31	0	0	0	0	0,025
0,9845	682,3	682,3	1364,5	1,25	1	0,077	Equal	10	31	0	0	0	0	0,025
0,8947	414,6	414,6	829,2	1,25	1	0,077	Equal	10	31	0	0	0	0	0,025
0,9342	483,7	483,7	967,4	1,25	1	0,077	Equal	10	31	0	0	0	0	0,025
0,9597	552,8	552,8	1105,6	1,25	1	0,077	Equal	10	31	0	0	0	0	0,025
0,9756	621,9	621,9	1243,8	1,25	1	0,077	Equal	10	31	0	0	0	0	0,025
0,9855	691,0	691,0	1382,0	1,25	1	0,077	Equal	10	31	0	0	0	0	0,025
0,8980	419,4	419,4	838,9	1,25	1	0,077	Equal	10	31	0	0	0	0	0,025
0,9368	489,3	489,3	978,7	1,25	1	0,077	Equal	10	31	0	0	0	0	0,025
0,9615	559,3	559,3	1118,5	1,25	1	0,077	Equal	10	31	0	0	0	0	0,025
0,9769	629,2	629,2	1258,3	1,25	1	0,077	Equal	10	31	0	0	0	0	0,025
0,9863	699,1	699,1	1398,1	1,25	1	0,077	Equal	10	31	0	0	0	0	0,025
0,9010	423,9	423,9	847,9	1,25	1	0,077	Equal	10	31	0	0	0	0	0,025

Non-Inferiority Logrank Tests

Numeric Results (Events) (Continued)

Solve For: Power
Test Type: Two-Sample Logrank Test
Groups: 1 = Reference, 2 = Treatment
Hypotheses: H0: HR ≥ HR0 vs. H1: HR < HR0

	Number of Events			Hazard Ratio		Ref. Group Hazard Rate	Accrual	Time		Proportion Lost*		Proportion Switching Groups*		
Power	E1	E2	E	Non-Inferiority HR0	Actual HR1	h1	Pattern	Accrual	Total	Ref.	Treat.	Ref. to Treat.	Treat. to Ref.	Alpha
0,9390	494,6	494,6	989,2	1,25	1	0,077	Equal	10	31	0	0	0	0	0,025
0,9631	565,2	565,2	1130,5	1,25	1	0,077	Equal	10	31	0	0	0	0	0,025
0,9780	635,9	635,9	1271,8	1,25	1	0,077	Equal	10	31	0	0	0	0	0,025
0,9871	706,5	706,5	1413,1	1,25	1	0,077	Equal	10	31	0	0	0	0	0,025
0,7516	279,6	279,6	559,2	1,25	1	0,077	Equal	10	31	0	0	0	0	0,025
0,8133	326,2	326,2	652,4	1,25	1	0,077	Equal	10	31	0	0	0	0	0,025
0,8613	372,8	372,8	745,6	1,25	1	0,077	Equal	10	31	0	0	0	0	0,025
0,8980	419,4	419,4	838,8	1,25	1	0,077	Equal	10	31	0	0	0	0	0,025
0,9257	466,0	466,0	932,0	1,25	1	0,077	Equal	10	31	0	0	0	0	0,025
0,7729	294,4	294,4	588,9	1,25	1	0,077	Equal	10	31	0	0	0	0	0,025
0,8326	343,5	343,5	687,0	1,25	1	0,077	Equal	10	31	0	0	0	0	0,025
0,8781	392,6	392,6	785,2	1,25	1	0,077	Equal	10	31	0	0	0	0	0,025
0,9122	441,7	441,7	883,3	1,25	1	0,077	Equal	10	31	0	0	0	0	0,025
0,9374	490,7	490,7	981,5	1,25	1	0,077	Equal	10	31	0	0	0	0	0,025
0,7912	308,2	308,2	616,4	1,25	1	0,077	Equal	10	31	0	0	0	0	0,025
0,8489	359,6	359,6	719,1	1,25	1	0,077	Equal	10	31	0	0	0	0	0,025
0,8921	410,9	410,9	821,9	1,25	1	0,077	Equal	10	31	0	0	0	0	0,025
0,9237	462,3	462,3	924,6	1,25	1	0,077	Equal	10	31	0	0	0	0	0,025
0,9467	513,7	513,7	1027,3	1,25	1	0,077	Equal	10	31	0	0	0	0	0,025
0,8071	320,9	320,9	641,9	1,25	1	0,077	Equal	10	31	0	0	0	0	0,025
0,8627	374,4	374,4	748,8	1,25	1	0,077	Equal	10	31	0	0	0	0	0,025
0,9037	427,9	427,9	855,8	1,25	1	0,077	Equal	10	31	0	0	0	0	0,025
0,9332	481,4	481,4	962,8	1,25	1	0,077	Equal	10	31	0	0	0	0	0,025
0,9541	534,9	534,9	1069,8	1,25	1	0,077	Equal	10	31	0	0	0	0	0,025
0,8208	332,7	332,7	665,4	1,25	1	0,077	Equal	10	31	0	0	0	0	0,025
0,8745	388,2	388,2	776,3	1,25	1	0,077	Equal	10	31	0	0	0	0	0,025
0,9134	443,6	443,6	887,3	1,25	1	0,077	Equal	10	31	0	0	0	0	0,025

Non-Inferiority Logrank Tests

Numeric Results (Events) (Continued)

Solve For: Power
Test Type: Two-Sample Logrank Test
Groups: 1 = Reference, 2 = Treatment
Hypotheses: H0: HR ≥ HR0 vs. H1: HR < HR0

	Number of Events			Hazard Ratio		Ref. Group Hazard Rate	Accrual	Time		Proportion Lost*		Proportion Switching Groups*		
Power	E1	E2	E	Non-Inferiority HR0	Actual HR1	h1	Pattern	Accrual	Total	Ref.	Treat.	Ref. to Treat.	Treat. to Ref.	Alpha
0,9409	499,1	499,1	998,2	1,25	1	0,077	Equal	10	31	0	0	0	0	0,025
0,9602	554,5	554,5	1109,1	1,25	1	0,077	Equal	10	31	0	0	0	0	0,025
0,8327	343,6	343,6	687,3	1,25	1	0,077	Equal	10	31	0	0	0	0	0,025
0,8846	400,9	400,9	801,8	1,25	1	0,077	Equal	10	31	0	0	0	0	0,025
0,9216	458,2	458,2	916,4	1,25	1	0,077	Equal	10	31	0	0	0	0	0,025
0,9474	515,5	515,5	1030,9	1,25	1	0,077	Equal	10	31	0	0	0	0	0,025
0,9651	572,7	572,7	1145,4	1,25	1	0,077	Equal	10	31	0	0	0	0	0,025
0,8432	353,7	353,7	707,5	1,25	1	0,077	Equal	10	31	0	0	0	0	0,025
0,8933	412,7	412,7	825,4	1,25	1	0,077	Equal	10	31	0	0	0	0	0,025
0,9285	471,7	471,7	943,3	1,25	1	0,077	Equal	10	31	0	0	0	0	0,025
0,9527	530,6	530,6	1061,2	1,25	1	0,077	Equal	10	31	0	0	0	0	0,025
0,9691	589,6	589,6	1179,1	1,25	1	0,077	Equal	10	31	0	0	0	0	0,025
0,8523	363,1	363,1	726,2	1,25	1	0,077	Equal	10	31	0	0	0	0	0,025
0,9008	423,6	423,6	847,2	1,25	1	0,077	Equal	10	31	0	0	0	0	0,025
0,9344	484,1	484,1	968,3	1,25	1	0,077	Equal	10	31	0	0	0	0	0,025
0,9572	544,6	544,6	1089,3	1,25	1	0,077	Equal	10	31	0	0	0	0	0,025
0,9724	605,2	605,2	1210,3	1,25	1	0,077	Equal	10	31	0	0	0	0	0,025
0,8604	371,8	371,8	743,5	1,25	1	0,077	Equal	10	31	0	0	0	0	0,025
0,9074	433,7	433,7	867,4	1,25	1	0,077	Equal	10	31	0	0	0	0	0,025
0,9395	495,7	495,7	991,4	1,25	1	0,077	Equal	10	31	0	0	0	0	0,025
0,9610	557,6	557,6	1115,3	1,25	1	0,077	Equal	10	31	0	0	0	0	0,025
0,9752	619,6	619,6	1239,2	1,25	1	0,077	Equal	10	31	0	0	0	0	0,025
0,8674	379,8	379,8	759,6	1,25	1	0,077	Equal	10	31	0	0	0	0	0,025
0,9130	443,1	443,1	886,2	1,25	1	0,077	Equal	10	31	0	0	0	0	0,025
0,9439	506,4	506,4	1012,8	1,25	1	0,077	Equal	10	31	0	0	0	0	0,025
0,9643	569,7	569,7	1139,3	1,25	1	0,077	Equal	10	31	0	0	0	0	0,025
0,9775	633,0	633,0	1265,9	1,25	1	0,077	Equal	10	31	0	0	0	0	0,025

Non-Inferiority Logrank Tests

Numeric Results (Events) (Continued)

Solve For: Power
Test Type: Two-Sample Logrank Test
Groups: 1 = Reference, 2 = Treatment
Hypotheses: H0: HR ≥ HR0 vs. H1: HR < HR0

	Number of Events			Hazard Ratio		Ref. Group Hazard Rate	Accrual	Time		Proportion Lost*		Proportion Switching Groups*		
Power	E1	E2	E	Non-Inferiority HR0	Actual HR1	h1	Pattern	Accrual	Total	Ref.	Treat.	Ref. to Treat.	Treat. to Ref.	Alpha
0,8737	387,2	387,2	774,4	1,25	1	0,077	Equal	10	31	0	0	0	0	0,025
0,9180	451,7	451,7	903,5	1,25	1	0,077	Equal	10	31	0	0	0	0	0,025
0,9477	516,3	516,3	1032,6	1,25	1	0,077	Equal	10	31	0	0	0	0	0,025
0,9671	580,8	580,8	1161,6	1,25	1	0,077	Equal	10	31	0	0	0	0	0,025
0,9795	645,3	645,3	1290,7	1,25	1	0,077	Equal	10	31	0	0	0	0	0,025
0,8793	394,1	394,1	788,2	1,25	1	0,077	Equal	10	31	0	0	0	0	0,025
0,9224	459,8	459,8	919,5	1,25	1	0,077	Equal	10	31	0	0	0	0	0,025
0,9509	525,4	525,4	1050,9	1,25	1	0,077	Equal	10	31	0	0	0	0	0,025
0,9694	591,1	591,1	1182,3	1,25	1	0,077	Equal	10	31	0	0	0	0	0,025
0,9812	656,8	656,8	1313,6	1,25	1	0,077	Equal	10	31	0	0	0	0	0,025
0,8843	400,5	400,5	800,9	1,25	1	0,077	Equal	10	31	0	0	0	0	0,025
0,9263	467,2	467,2	934,4	1,25	1	0,077	Equal	10	31	0	0	0	0	0,025
0,9538	533,9	533,9	1067,9	1,25	1	0,077	Equal	10	31	0	0	0	0	0,025
0,9715	600,7	600,7	1201,4	1,25	1	0,077	Equal	10	31	0	0	0	0	0,025
0,9826	667,4	667,4	1334,8	1,25	1	0,077	Equal	10	31	0	0	0	0	0,025
0,8887	406,3	406,3	812,7	1,25	1	0,077	Equal	10	31	0	0	0	0	0,025
0,9297	474,1	474,1	948,1	1,25	1	0,077	Equal	10	31	0	0	0	0	0,025
0,9563	541,8	541,8	1083,6	1,25	1	0,077	Equal	10	31	0	0	0	0	0,025
0,9733	609,5	609,5	1219,0	1,25	1	0,077	Equal	10	31	0	0	0	0	0,025
0,9839	677,2	677,2	1354,5	1,25	1	0,077	Equal	10	31	0	0	0	0	0,025
0,8927	411,8	411,8	823,6	1,25	1	0,077	Equal	10	31	0	0	0	0	0,025
0,9327	480,4	480,4	960,9	1,25	1	0,077	Equal	10	31	0	0	0	0	0,025
0,9586	549,1	549,1	1098,2	1,25	1	0,077	Equal	10	31	0	0	0	0	0,025
0,9749	617,7	617,7	1235,4	1,25	1	0,077	Equal	10	31	0	0	0	0	0,025
0,9850	686,3	686,3	1372,7	1,25	1	0,077	Equal	10	31	0	0	0	0	0,025
0,8962	416,9	416,9	833,7	1,25	1	0,077	Equal	10	31	0	0	0	0	0,025
0,9354	486,3	486,3	972,7	1,25	1	0,077	Equal	10	31	0	0	0	0	0,025

Non-Inferiority Logrank Tests

Numeric Results (Events) (Continued)

Solve For: Power
Test Type: Two-Sample Logrank Test
Groups: 1 = Reference, 2 = Treatment
Hypotheses: H0: HR ≥ HR0 vs. H1: HR < HR0

	Number of Events			Hazard Ratio		Ref. Group Hazard Rate		Time		Proportion Lost*		Proportion Switching Groups*		
**Power**	**E1**	**E2**	**E**	**Non-Inferiority HR0**	**Actual HR1**	**h1**	**Accrual Pattern**	**Accrual**	**Total**	**Ref.**	**Treat.**	**Ref. to Treat.**	**Treat. to Ref.**	**Alpha**
0,9605	555,8	555,8	1111,6	1,25	1	0,077	Equal	10	31	0	0	0	0	0,025
0,9762	625,3	625,3	1250,6	1,25	1	0,077	Equal	10	31	0	0	0	0	0,025
0,9859	694,8	694,8	1389,5	1,25	1	0,077	Equal	10	31	0	0	0	0	0,025
0,8994	421,5	421,5	843,1	1,25	1	0,077	Equal	10	31	0	0	0	0	0,025
0,9378	491,8	491,8	983,6	1,25	1	0,077	Equal	10	31	0	0	0	0	0,025
0,9623	562,1	562,1	1124,1	1,25	1	0,077	Equal	10	31	0	0	0	0	0,025
0,9774	632,3	632,3	1264,6	1,25	1	0,077	Equal	10	31	0	0	0	0	0,025
0,9867	702,6	702,6	1405,1	1,25	1	0,077	Equal	10	31	0	0	0	0	0,025
0,7385	271,0	271,0	541,9	1,25	1	0,077	Equal	10	31	0	0	0	0	0,025
0,8012	316,1	316,1	632,2	1,25	1	0,077	Equal	10	31	0	0	0	0	0,025
0,8506	361,3	361,3	722,6	1,25	1	0,077	Equal	10	31	0	0	0	0	0,025
0,8888	406,4	406,4	812,9	1,25	1	0,077	Equal	10	31	0	0	0	0	0,025
0,9180	451,6	451,6	903,2	1,25	1	0,077	Equal	10	31	0	0	0	0	0,025
0,7617	286,5	286,5	572,9	1,25	1	0,077	Equal	10	31	0	0	0	0	0,025
0,8225	334,2	334,2	668,4	1,25	1	0,077	Equal	10	31	0	0	0	0	0,025
0,8693	381,9	381,9	763,9	1,25	1	0,077	Equal	10	31	0	0	0	0	0,025
0,9048	429,7	429,7	859,4	1,25	1	0,077	Equal	10	31	0	0	0	0	0,025
0,9313	477,4	477,4	954,8	1,25	1	0,077	Equal	10	31	0	0	0	0	0,025
0,7815	300,8	300,8	601,6	1,25	1	0,077	Equal	10	31	0	0	0	0	0,025
0,8403	350,9	350,9	701,9	1,25	1	0,077	Equal	10	31	0	0	0	0	0,025
0,8847	401,1	401,1	802,1	1,25	1	0,077	Equal	10	31	0	0	0	0	0,025
0,9177	451,2	451,2	902,4	1,25	1	0,077	Equal	10	31	0	0	0	0	0,025
0,9418	501,3	501,3	1002,7	1,25	1	0,077	Equal	10	31	0	0	0	0	0,025
0,7987	314,1	314,1	628,2	1,25	1	0,077	Equal	10	31	0	0	0	0	0,025
0,8554	366,4	366,4	732,8	1,25	1	0,077	Equal	10	31	0	0	0	0	0,025
0,8976	418,8	418,8	837,5	1,25	1	0,077	Equal	10	31	0	0	0	0	0,025
0,9282	471,1	471,1	942,2	1,25	1	0,077	Equal	10	31	0	0	0	0	0,025

Non-Inferiority Logrank Tests

Numeric Results (Events) (Continued)

Solve For: Power
Test Type: Two-Sample Logrank Test
Groups: 1 = Reference, 2 = Treatment
Hypotheses: H0: HR ≥ HR0 vs. H1: HR < HR0

	Number of Events			Hazard Ratio		Ref. Group Hazard Rate		Time		Proportion Lost*		Proportion Switching Groups*		
Power	E1	E2	E	Non-Inferiority HR0	Actual HR1	h1	Accrual Pattern	Accrual	Total	Ref.	Treat.	Ref. to Treat.	Treat. to Ref.	Alpha
0,9503	523,5	523,5	1046,9	1,25	1	0,077	Equal	10	31	0	0	0	0	0,025
0,8135	326,4	326,4	652,7	1,25	1	0,077	Equal	10	31	0	0	0	0	0,025
0,8683	380,8	380,8	761,5	1,25	1	0,077	Equal	10	31	0	0	0	0	0,025
0,9083	435,2	435,2	870,3	1,25	1	0,077	Equal	10	31	0	0	0	0	0,025
0,9369	489,6	489,6	979,1	1,25	1	0,077	Equal	10	31	0	0	0	0	0,025
0,9570	544,0	544,0	1087,9	1,25	1	0,077	Equal	10	31	0	0	0	0	0,025
0,8264	337,8	337,8	675,5	1,25	1	0,077	Equal	10	31	0	0	0	0	0,025
0,8793	394,1	394,1	788,1	1,25	1	0,077	Equal	10	31	0	0	0	0	0,025
0,9172	450,3	450,3	900,7	1,25	1	0,077	Equal	10	31	0	0	0	0	0,025
0,9440	506,6	506,6	1013,3	1,25	1	0,077	Equal	10	31	0	0	0	0	0,025
0,9625	562,9	562,9	1125,9	1,25	1	0,077	Equal	10	31	0	0	0	0	0,025
0,8376	348,3	348,3	696,6	1,25	1	0,077	Equal	10	31	0	0	0	0	0,025
0,8887	406,3	406,3	812,7	1,25	1	0,077	Equal	10	31	0	0	0	0	0,025
0,9248	464,4	464,4	928,8	1,25	1	0,077	Equal	10	31	0	0	0	0	0,025
0,9499	522,4	522,4	1044,9	1,25	1	0,077	Equal	10	31	0	0	0	0	0,025
0,9670	580,5	580,5	1161,0	1,25	1	0,077	Equal	10	31	0	0	0	0	0,025
0,8474	358,1	358,1	716,1	1,25	1	0,077	Equal	10	31	0	0	0	0	0,025
0,8968	417,7	417,7	835,5	1,25	1	0,077	Equal	10	31	0	0	0	0	0,025
0,9313	477,4	477,4	954,8	1,25	1	0,077	Equal	10	31	0	0	0	0	0,025
0,9548	537,1	537,1	1074,2	1,25	1	0,077	Equal	10	31	0	0	0	0	0,025
0,9707	596,8	596,8	1193,5	1,25	1	0,077	Equal	10	31	0	0	0	0	0,025
0,8561	367,1	367,1	734,2	1,25	1	0,077	Equal	10	31	0	0	0	0	0,025
0,9039	428,3	428,3	856,6	1,25	1	0,077	Equal	10	31	0	0	0	0	0,025
0,9368	489,5	489,5	978,9	1,25	1	0,077	Equal	10	31	0	0	0	0	0,025
0,9590	550,6	550,6	1101,3	1,25	1	0,077	Equal	10	31	0	0	0	0	0,025
0,9737	611,8	611,8	1223,7	1,25	1	0,077	Equal	10	31	0	0	0	0	0,025
0,8637	375,5	375,5	750,9	1,25	1	0,077	Equal	10	31	0	0	0	0	0,025

Non-Inferiority Logrank Tests

Numeric Results (Events) (Continued)

Solve For: Power
Test Type: Two-Sample Logrank Test
Groups: 1 = Reference, 2 = Treatment
Hypotheses: H0: HR ≥ HR0 vs. H1: HR < HR0

	Number of Events			Hazard Ratio		Ref. Group Hazard Rate	Accrual	Time		Proportion Lost*		Proportion Switching Groups*		
Power	E1	E2	E	Non-Inferiority HR0	Actual HR1	h1	Pattern	Accrual	Total	Ref.	Treat.	Ref. to Treat.	Treat. to Ref.	Alpha
0,9100	438,0	438,0	876,1	1,25	1	0,077	Equal	10	31	0	0	0	0	0,025
0,9416	500,6	500,6	1001,2	1,25	1	0,077	Equal	10	31	0	0	0	0	0,025
0,9626	563,2	563,2	1126,4	1,25	1	0,077	Equal	10	31	0	0	0	0	0,025
0,9763	625,8	625,8	1251,5	1,25	1	0,077	Equal	10	31	0	0	0	0	0,025
0,8704	383,2	383,2	766,4	1,25	1	0,077	Equal	10	31	0	0	0	0	0,025
0,9154	447,1	447,1	894,2	1,25	1	0,077	Equal	10	31	0	0	0	0	0,025
0,9457	510,9	510,9	1021,9	1,25	1	0,077	Equal	10	31	0	0	0	0	0,025
0,9656	574,8	574,8	1149,6	1,25	1	0,077	Equal	10	31	0	0	0	0	0,025
0,9785	638,7	638,7	1277,4	1,25	1	0,077	Equal	10	31	0	0	0	0	0,025
0,8763	390,4	390,4	780,8	1,25	1	0,077	Equal	10	31	0	0	0	0	0,025
0,9201	455,4	455,4	910,9	1,25	1	0,077	Equal	10	31	0	0	0	0	0,025
0,9492	520,5	520,5	1041,0	1,25	1	0,077	Equal	10	31	0	0	0	0	0,025
0,9682	585,6	585,6	1171,2	1,25	1	0,077	Equal	10	31	0	0	0	0	0,025
0,9803	650,6	650,6	1301,3	1,25	1	0,077	Equal	10	31	0	0	0	0	0,025
0,8816	397,0	397,0	794,1	1,25	1	0,077	Equal	10	31	0	0	0	0	0,025
0,9242	463,2	463,2	926,4	1,25	1	0,077	Equal	10	31	0	0	0	0	0,025
0,9523	529,4	529,4	1058,7	1,25	1	0,077	Equal	10	31	0	0	0	0	0,025
0,9704	595,5	595,5	1191,1	1,25	1	0,077	Equal	10	31	0	0	0	0	0,025
0,9819	661,7	661,7	1323,4	1,25	1	0,077	Equal	10	31	0	0	0	0	0,025
0,8863	403,2	403,2	806,4	1,25	1	0,077	Equal	10	31	0	0	0	0	0,025
0,9279	470,4	470,4	940,7	1,25	1	0,077	Equal	10	31	0	0	0	0	0,025
0,9550	537,6	537,6	1075,1	1,25	1	0,077	Equal	10	31	0	0	0	0	0,025
0,9723	604,8	604,8	1209,5	1,25	1	0,077	Equal	10	31	0	0	0	0	0,025
0,9832	672,0	672,0	1343,9	1,25	1	0,077	Equal	10	31	0	0	0	0	0,025
0,8906	408,9	408,9	817,7	1,25	1	0,077	Equal	10	31	0	0	0	0	0,025
0,9311	477,0	477,0	954,0	1,25	1	0,077	Equal	10	31	0	0	0	0	0,025
0,9574	545,2	545,2	1090,3	1,25	1	0,077	Equal	10	31	0	0	0	0	0,025

Non-Inferiority Logrank Tests

Numeric Results (Events) (Continued)

Solve For: Power
Test Type: Two-Sample Logrank Test
Groups: 1 = Reference, 2 = Treatment
Hypotheses: H0: HR ≥ HR0 vs. H1: HR < HR0

	Number of Events			Hazard Ratio		Ref. Group Hazard Rate		Time		Proportion Lost*		Proportion Switching Groups*		
Power	E1	E2	E	Non-Inferiority HR0	Actual HR1	h1	Accrual Pattern	Accrual	Total	Ref.	Treat.	Ref. to Treat.	Treat. to Ref.	Alpha
0,9740	613,3	613,3	1226,6	1,25	1	0,077	Equal	10	31	0	0	0	0	0,025
0,9844	681,4	681,4	1362,9	1,25	1	0,077	Equal	10	31	0	0	0	0	0,025
0,8943	414,1	414,1	828,3	1,25	1	0,077	Equal	10	31	0	0	0	0	0,025
0,9340	483,2	483,2	966,3	1,25	1	0,077	Equal	10	31	0	0	0	0	0,025
0,9595	552,2	552,2	1104,4	1,25	1	0,077	Equal	10	31	0	0	0	0	0,025
0,9755	621,2	621,2	1242,4	1,25	1	0,077	Equal	10	31	0	0	0	0	0,025
0,9854	690,2	690,2	1380,5	1,25	1	0,077	Equal	10	31	0	0	0	0	0,025
0,8977	419,0	419,0	838,0	1,25	1	0,077	Equal	10	31	0	0	0	0	0,025
0,9366	488,9	488,9	977,7	1,25	1	0,077	Equal	10	31	0	0	0	0	0,025
0,9613	558,7	558,7	1117,4	1,25	1	0,077	Equal	10	31	0	0	0	0	0,025
0,9768	628,5	628,5	1257,1	1,25	1	0,077	Equal	10	31	0	0	0	0	0,025
0,9863	698,4	698,4	1396,7	1,25	1	0,077	Equal	10	31	0	0	0	0	0,025
0,7240	261,8	261,8	523,7	1,25	1	0,077	Equal	10	31	0	0	0	0	0,025
0,7877	305,5	305,5	611,0	1,25	1	0,077	Equal	10	31	0	0	0	0	0,025
0,8385	349,1	349,1	698,3	1,25	1	0,077	Equal	10	31	0	0	0	0	0,025
0,8783	392,8	392,8	785,5	1,25	1	0,077	Equal	10	31	0	0	0	0	0,025
0,9090	436,4	436,4	872,8	1,25	1	0,077	Equal	10	31	0	0	0	0	0,025
0,7493	278,0	278,0	556,0	1,25	1	0,077	Equal	10	31	0	0	0	0	0,025
0,8111	324,3	324,3	648,7	1,25	1	0,077	Equal	10	31	0	0	0	0	0,025
0,8594	370,7	370,7	741,4	1,25	1	0,077	Equal	10	31	0	0	0	0	0,025
0,8964	417,0	417,0	834,0	1,25	1	0,077	Equal	10	31	0	0	0	0	0,025
0,9243	463,4	463,4	926,7	1,25	1	0,077	Equal	10	31	0	0	0	0	0,025
0,7709	293,0	293,0	586,0	1,25	1	0,077	Equal	10	31	0	0	0	0	0,025
0,8308	341,8	341,8	683,6	1,25	1	0,077	Equal	10	31	0	0	0	0	0,025
0,8765	390,6	390,6	781,3	1,25	1	0,077	Equal	10	31	0	0	0	0	0,025
0,9109	439,5	439,5	878,9	1,25	1	0,077	Equal	10	31	0	0	0	0	0,025
0,9363	488,3	488,3	976,6	1,25	1	0,077	Equal	10	31	0	0	0	0	0,025

Non-Inferiority Logrank Tests

Numeric Results (Events) (Continued)

Solve For: Power
Test Type: Two-Sample Logrank Test
Groups: 1 = Reference, 2 = Treatment
Hypotheses: H0: HR ≥ HR0 vs. H1: HR < HR0

	Number of Events			Hazard Ratio		Ref. Group Hazard Rate		Time		Proportion Lost*		Proportion Switching Groups*		
Power	E1	E2	E	Non-Inferiority HR0	Actual HR1	h1	Accrual Pattern	Accrual	Total	Ref.	Treat.	Ref. to Treat.	Treat. to Ref.	Alpha
0,7895	306,8	306,8	613,7	1,25	1	0,077	Equal	10	31	0	0	0	0	0,025
0,8474	358,0	358,0	716,0	1,25	1	0,077	Equal	10	31	0	0	0	0	0,025
0,8907	409,1	409,1	818,2	1,25	1	0,077	Equal	10	31	0	0	0	0	0,025
0,9227	460,3	460,3	920,5	1,25	1	0,077	Equal	10	31	0	0	0	0	0,025
0,9458	511,4	511,4	1022,8	1,25	1	0,077	Equal	10	31	0	0	0	0	0,025
0,8056	319,7	319,7	639,4	1,25	1	0,077	Equal	10	31	0	0	0	0	0,025
0,8614	373,0	373,0	745,9	1,25	1	0,077	Equal	10	31	0	0	0	0	0,025
0,9026	426,2	426,2	852,5	1,25	1	0,077	Equal	10	31	0	0	0	0	0,025
0,9323	479,5	479,5	959,0	1,25	1	0,077	Equal	10	31	0	0	0	0	0,025
0,9534	532,8	532,8	1065,6	1,25	1	0,077	Equal	10	31	0	0	0	0	0,025
0,8195	331,6	331,6	663,1	1,25	1	0,077	Equal	10	31	0	0	0	0	0,025
0,8734	386,8	386,8	773,6	1,25	1	0,077	Equal	10	31	0	0	0	0	0,025
0,9125	442,1	442,1	884,2	1,25	1	0,077	Equal	10	31	0	0	0	0	0,025
0,9402	497,3	497,3	994,7	1,25	1	0,077	Equal	10	31	0	0	0	0	0,025
0,9596	552,6	552,6	1105,2	1,25	1	0,077	Equal	10	31	0	0	0	0	0,025
0,8316	342,6	342,6	685,1	1,25	1	0,077	Equal	10	31	0	0	0	0	0,025
0,8837	399,7	399,7	799,3	1,25	1	0,077	Equal	10	31	0	0	0	0	0,025
0,9208	456,7	456,7	913,5	1,25	1	0,077	Equal	10	31	0	0	0	0	0,025
0,9467	513,8	513,8	1027,7	1,25	1	0,077	Equal	10	31	0	0	0	0	0,025
0,9646	570,9	570,9	1141,9	1,25	1	0,077	Equal	10	31	0	0	0	0	0,025
0,8422	352,7	352,7	705,5	1,25	1	0,077	Equal	10	31	0	0	0	0	0,025
0,8925	411,5	411,5	823,1	1,25	1	0,077	Equal	10	31	0	0	0	0	0,025
0,9278	470,3	470,3	940,7	1,25	1	0,077	Equal	10	31	0	0	0	0	0,025
0,9522	529,1	529,1	1058,2	1,25	1	0,077	Equal	10	31	0	0	0	0	0,025
0,9687	587,9	587,9	1175,8	1,25	1	0,077	Equal	10	31	0	0	0	0	0,025
0,8514	362,2	362,2	724,4	1,25	1	0,077	Equal	10	31	0	0	0	0	0,025
0,9001	422,5	422,5	845,1	1,25	1	0,077	Equal	10	31	0	0	0	0	0,025

Non-Inferiority Logrank Tests

Numeric Results (Events) (Continued)

Solve For: Power
Test Type: Two-Sample Logrank Test
Groups: 1 = Reference, 2 = Treatment
Hypotheses: H0: HR ≥ HR0 vs. H1: HR < HR0

	Number of Events			Hazard Ratio		Ref. Group Hazard Rate	Accrual	Time		Proportion Lost*		Proportion Switching Groups*		
Power	E1	E2	E	Non-Inferiority HR0	Actual HR1	h1	Pattern	Accrual	Total	Ref.	Treat.	Ref. to Treat.	Treat. to Ref.	Alpha
0,9339	482,9	482,9	965,8	1,25	1	0,077	Equal	10	31	0	0	0	0	0,025
0,9568	543,3	543,3	1086,5	1,25	1	0,077	Equal	10	31	0	0	0	0	0,025
0,9721	603,6	603,6	1207,3	1,25	1	0,077	Equal	10	31	0	0	0	0	0,025
0,8596	370,9	370,9	741,8	1,25	1	0,077	Equal	10	31	0	0	0	0	0,025
0,9067	432,7	432,7	865,5	1,25	1	0,077	Equal	10	31	0	0	0	0	0,025
0,9390	494,5	494,5	989,1	1,25	1	0,077	Equal	10	31	0	0	0	0	0,025
0,9607	556,4	556,4	1112,7	1,25	1	0,077	Equal	10	31	0	0	0	0	0,025
0,9749	618,2	618,2	1236,4	1,25	1	0,077	Equal	10	31	0	0	0	0	0,025
0,8668	379,0	379,0	758,0	1,25	1	0,077	Equal	10	31	0	0	0	0	0,025
0,9125	442,2	442,2	884,3	1,25	1	0,077	Equal	10	31	0	0	0	0	0,025
0,9435	505,3	505,3	1010,6	1,25	1	0,077	Equal	10	31	0	0	0	0	0,025
0,9640	568,5	568,5	1137,0	1,25	1	0,077	Equal	10	31	0	0	0	0	0,025
0,9773	631,7	631,7	1263,3	1,25	1	0,077	Equal	10	31	0	0	0	0	0,025
0,8731	386,5	386,5	773,0	1,25	1	0,077	Equal	10	31	0	0	0	0	0,025
0,9176	450,9	450,9	901,8	1,25	1	0,077	Equal	10	31	0	0	0	0	0,025
0,9473	515,3	515,3	1030,6	1,25	1	0,077	Equal	10	31	0	0	0	0	0,025
0,9668	579,7	579,7	1159,4	1,25	1	0,077	Equal	10	31	0	0	0	0	0,025
0,9793	644,1	644,1	1288,3	1,25	1	0,077	Equal	10	31	0	0	0	0	0,025
0,8788	393,4	393,4	786,8	1,25	1	0,077	Equal	10	31	0	0	0	0	0,025
0,9220	459,0	459,0	918,0	1,25	1	0,077	Equal	10	31	0	0	0	0	0,025
0,9506	524,5	524,5	1049,1	1,25	1	0,077	Equal	10	31	0	0	0	0	0,025
0,9692	590,1	590,1	1180,2	1,25	1	0,077	Equal	10	31	0	0	0	0	0,025
0,9810	655,7	655,7	1311,4	1,25	1	0,077	Equal	10	31	0	0	0	0	0,025
0,8838	399,8	399,8	799,7	1,25	1	0,077	Equal	10	31	0	0	0	0	0,025
0,9259	466,5	466,5	932,9	1,25	1	0,077	Equal	10	31	0	0	0	0	0,025
0,9535	533,1	533,1	1066,2	1,25	1	0,077	Equal	10	31	0	0	0	0	0,025
0,9713	599,7	599,7	1199,5	1,25	1	0,077	Equal	10	31	0	0	0	0	0,025

Non-Inferiority Logrank Tests

Numeric Results (Events) (Continued)

Solve For: Power
Test Type: Two-Sample Logrank Test
Groups: 1 = Reference, 2 = Treatment
Hypotheses: H0: HR ≥ HR0 vs. H1: HR < HR0

	Number of Events			Hazard Ratio		Ref. Group Hazard Rate	Accrual	Time		Proportion Lost*		Proportion Switching Groups*		
Power	E1	E2	E	Non-Inferiority HR0	Actual HR1	h1	Pattern	Accrual	Total	Ref.	Treat.	Ref. to Treat.	Treat. to Ref.	Alpha
0,9825	666,4	666,4	1332,8	1,25	1	0,077	Equal	10	31	0	0	0	0	0,025
0,8883	405,8	405,8	811,5	1,25	1	0,077	Equal	10	31	0	0	0	0	0,025
0,9294	473,4	473,4	946,8	1,25	1	0,077	Equal	10	31	0	0	0	0	0,025
0,9561	541,0	541,0	1082,0	1,25	1	0,077	Equal	10	31	0	0	0	0	0,025
0,9731	608,7	608,7	1217,3	1,25	1	0,077	Equal	10	31	0	0	0	0	0,025
0,9838	676,3	676,3	1352,6	1,25	1	0,077	Equal	10	31	0	0	0	0	0,025
0,8923	411,3	411,3	822,5	1,25	1	0,077	Equal	10	31	0	0	0	0	0,025
0,9324	479,8	479,8	959,6	1,25	1	0,077	Equal	10	31	0	0	0	0	0,025
0,9583	548,4	548,4	1096,7	1,25	1	0,077	Equal	10	31	0	0	0	0	0,025
0,9747	616,9	616,9	1233,8	1,25	1	0,077	Equal	10	31	0	0	0	0	0,025
0,9848	685,4	685,4	1370,9	1,25	1	0,077	Equal	10	31	0	0	0	0	0,025
0,8959	416,4	416,4	832,7	1,25	1	0,077	Equal	10	31	0	0	0	0	0,025
0,9352	485,8	485,8	971,5	1,25	1	0,077	Equal	10	31	0	0	0	0	0,025
0,9603	555,2	555,2	1110,3	1,25	1	0,077	Equal	10	31	0	0	0	0	0,025
0,9761	624,5	624,5	1249,1	1,25	1	0,077	Equal	10	31	0	0	0	0	0,025
0,9858	693,9	693,9	1387,9	1,25	1	0,077	Equal	10	31	0	0	0	0	0,025
0,7081	252,2	252,2	504,5	1,25	1	0,077	Equal	10	31	0	0	0	0	0,025
0,7727	294,3	294,3	588,5	1,25	1	0,077	Equal	10	31	0	0	0	0	0,025
0,8248	336,3	336,3	672,6	1,25	1	0,077	Equal	10	31	0	0	0	0	0,025
0,8662	378,3	378,3	756,7	1,25	1	0,077	Equal	10	31	0	0	0	0	0,025
0,8987	420,4	420,4	840,8	1,25	1	0,077	Equal	10	31	0	0	0	0	0,025
0,7356	269,1	269,1	538,2	1,25	1	0,077	Equal	10	31	0	0	0	0	0,025
0,7985	314,0	314,0	627,9	1,25	1	0,077	Equal	10	31	0	0	0	0	0,025
0,8482	358,8	358,8	717,6	1,25	1	0,077	Equal	10	31	0	0	0	0	0,025
0,8867	403,7	403,7	807,3	1,25	1	0,077	Equal	10	31	0	0	0	0	0,025
0,9162	448,5	448,5	897,0	1,25	1	0,077	Equal	10	31	0	0	0	0	0,025
0,7592	284,7	284,7	569,5	1,25	1	0,077	Equal	10	31	0	0	0	0	0,025

Non-Inferiority Logrank Tests

Numeric Results (Events) (Continued)

Solve For: Power
Test Type: Two-Sample Logrank Test
Groups: 1 = Reference, 2 = Treatment
Hypotheses: H0: HR ≥ HR0 vs. H1: HR < HR0

	Number of Events			Hazard Ratio		Ref. Group Hazard Rate	Accrual	Time		Proportion Lost*		Proportion Switching Groups*		
Power	E1	E2	E	Non-Inferiority HR0	Actual HR1	h1	Pattern	Accrual	Total	Ref.	Treat.	Ref. to Treat.	Treat. to Ref.	Alpha
0,8202	332,2	332,2	664,4	1,25	1	0,077	Equal	10	31	0	0	0	0	0,025
0,8673	379,6	379,6	759,3	1,25	1	0,077	Equal	10	31	0	0	0	0	0,025
0,9031	427,1	427,1	854,2	1,25	1	0,077	Equal	10	31	0	0	0	0	0,025
0,9299	474,6	474,6	949,1	1,25	1	0,077	Equal	10	31	0	0	0	0	0,025
0,7794	299,2	299,2	598,4	1,25	1	0,077	Equal	10	31	0	0	0	0	0,025
0,8384	349,1	349,1	698,2	1,25	1	0,077	Equal	10	31	0	0	0	0	0,025
0,8831	398,9	398,9	797,9	1,25	1	0,077	Equal	10	31	0	0	0	0	0,025
0,9164	448,8	448,8	897,6	1,25	1	0,077	Equal	10	31	0	0	0	0	0,025
0,9408	498,7	498,7	997,4	1,25	1	0,077	Equal	10	31	0	0	0	0	0,025
0,7968	312,6	312,6	625,2	1,25	1	0,077	Equal	10	31	0	0	0	0	0,025
0,8538	364,7	364,7	729,4	1,25	1	0,077	Equal	10	31	0	0	0	0	0,025
0,8962	416,8	416,8	833,6	1,25	1	0,077	Equal	10	31	0	0	0	0	0,025
0,9271	468,9	468,9	937,8	1,25	1	0,077	Equal	10	31	0	0	0	0	0,025
0,9494	521,0	521,0	1042,0	1,25	1	0,077	Equal	10	31	0	0	0	0	0,025
0,8119	325,0	325,0	650,0	1,25	1	0,077	Equal	10	31	0	0	0	0	0,025
0,8669	379,2	379,2	758,4	1,25	1	0,077	Equal	10	31	0	0	0	0	0,025
0,9071	433,3	433,3	866,7	1,25	1	0,077	Equal	10	31	0	0	0	0	0,025
0,9360	487,5	487,5	975,0	1,25	1	0,077	Equal	10	31	0	0	0	0	0,025
0,9563	541,7	541,7	1083,4	1,25	1	0,077	Equal	10	31	0	0	0	0	0,025
0,8250	336,5	336,5	673,0	1,25	1	0,077	Equal	10	31	0	0	0	0	0,025
0,8781	392,6	392,6	785,2	1,25	1	0,077	Equal	10	31	0	0	0	0	0,025
0,9163	448,7	448,7	897,3	1,25	1	0,077	Equal	10	31	0	0	0	0	0,025
0,9432	504,7	504,7	1009,5	1,25	1	0,077	Equal	10	31	0	0	0	0	0,025
0,9619	560,8	560,8	1121,7	1,25	1	0,077	Equal	10	31	0	0	0	0	0,025
0,8364	347,1	347,1	694,3	1,25	1	0,077	Equal	10	31	0	0	0	0	0,025
0,8877	405,0	405,0	810,0	1,25	1	0,077	Equal	10	31	0	0	0	0	0,025
0,9240	462,8	462,8	925,7	1,25	1	0,077	Equal	10	31	0	0	0	0	0,025

Non-Inferiority Logrank Tests

Numeric Results (Events) (Continued)

Solve For: Power
Test Type: Two-Sample Logrank Test
Groups: 1 = Reference, 2 = Treatment
Hypotheses: H0: HR ≥ HR0 vs. H1: HR < HR0

	Number of Events			Hazard Ratio		Ref. Group Hazard Rate		Time		Proportion Lost*		Proportion Switching Groups*		
Power	E1	E2	E	Non-Inferiority HR0	Actual HR1	h1	Accrual Pattern	Accrual	Total	Ref.	Treat.	Ref. to Treat.	Treat. to Ref.	Alpha
0,9493	520,7	520,7	1041,4	1,25	1	0,077	Equal	10	31	0	0	0	0	0,025
0,9665	578,6	578,6	1157,1	1,25	1	0,077	Equal	10	31	0	0	0	0	0,025
0,8464	357,0	357,0	714,0	1,25	1	0,077	Equal	10	31	0	0	0	0	0,025
0,8960	416,5	416,5	833,0	1,25	1	0,077	Equal	10	31	0	0	0	0	0,025
0,9306	476,0	476,0	951,9	1,25	1	0,077	Equal	10	31	0	0	0	0	0,025
0,9543	535,5	535,5	1070,9	1,25	1	0,077	Equal	10	31	0	0	0	0	0,025
0,9703	595,0	595,0	1189,9	1,25	1	0,077	Equal	10	31	0	0	0	0	0,025
0,8551	366,1	366,1	732,2	1,25	1	0,077	Equal	10	31	0	0	0	0	0,025
0,9031	427,1	427,1	854,2	1,25	1	0,077	Equal	10	31	0	0	0	0	0,025
0,9362	488,1	488,1	976,3	1,25	1	0,077	Equal	10	31	0	0	0	0	0,025
0,9586	549,1	549,1	1098,3	1,25	1	0,077	Equal	10	31	0	0	0	0	0,025
0,9734	610,2	610,2	1220,3	1,25	1	0,077	Equal	10	31	0	0	0	0	0,025
0,8628	374,5	374,5	749,1	1,25	1	0,077	Equal	10	31	0	0	0	0	0,025
0,9094	437,0	437,0	873,9	1,25	1	0,077	Equal	10	31	0	0	0	0	0,025
0,9411	499,4	499,4	998,8	1,25	1	0,077	Equal	10	31	0	0	0	0	0,025
0,9622	561,8	561,8	1123,6	1,25	1	0,077	Equal	10	31	0	0	0	0	0,025
0,9760	624,2	624,2	1248,5	1,25	1	0,077	Equal	10	31	0	0	0	0	0,025
0,8697	382,4	382,4	764,7	1,25	1	0,077	Equal	10	31	0	0	0	0	0,025
0,9148	446,1	446,1	892,2	1,25	1	0,077	Equal	10	31	0	0	0	0	0,025
0,9452	509,8	509,8	1019,6	1,25	1	0,077	Equal	10	31	0	0	0	0	0,025
0,9653	573,5	573,5	1147,1	1,25	1	0,077	Equal	10	31	0	0	0	0	0,025
0,9782	637,3	637,3	1274,5	1,25	1	0,077	Equal	10	31	0	0	0	0	0,025
0,8757	389,6	389,6	779,2	1,25	1	0,077	Equal	10	31	0	0	0	0	0,025
0,9196	454,5	454,5	909,0	1,25	1	0,077	Equal	10	31	0	0	0	0	0,025
0,9488	519,5	519,5	1038,9	1,25	1	0,077	Equal	10	31	0	0	0	0	0,025
0,9679	584,4	584,4	1168,8	1,25	1	0,077	Equal	10	31	0	0	0	0	0,025
0,9801	649,3	649,3	1298,6	1,25	1	0,077	Equal	10	31	0	0	0	0	0,025

Non-Inferiority Logrank Tests

Numeric Results (Events) (Continued)

Solve For: Power
Test Type: Two-Sample Logrank Test
Groups: 1 = Reference, 2 = Treatment
Hypotheses: H0: HR ≥ HR0 vs. H1: HR < HR0

	Number of Events			Hazard Ratio		Ref. Group Hazard Rate		Time		Proportion Lost*		Proportion Switching Groups*		
Power	E1	E2	E	Non-Inferiority HR0	Actual HR1	h1	Accrual Pattern	Accrual	Total	Ref.	Treat.	Ref. to Treat.	Treat. to Ref.	Alpha
0,8811	396,3	396,3	792,6	1,25	1	0,077	Equal	10	31	0	0	0	0	0,025
0,9238	462,3	462,3	924,7	1,25	1	0,077	Equal	10	31	0	0	0	0	0,025
0,9520	528,4	528,4	1056,8	1,25	1	0,077	Equal	10	31	0	0	0	0	0,025
0,9702	594,4	594,4	1188,9	1,25	1	0,077	Equal	10	31	0	0	0	0	0,025
0,9817	660,5	660,5	1321,0	1,25	1	0,077	Equal	10	31	0	0	0	0	0,025
0,8858	402,5	402,5	805,0	1,25	1	0,077	Equal	10	31	0	0	0	0	0,025
0,9275	469,6	469,6	939,2	1,25	1	0,077	Equal	10	31	0	0	0	0	0,025
0,9547	536,7	536,7	1073,3	1,25	1	0,077	Equal	10	31	0	0	0	0	0,025
0,9721	603,7	603,7	1207,5	1,25	1	0,077	Equal	10	31	0	0	0	0	0,025
0,9831	670,8	670,8	1341,6	1,25	1	0,077	Equal	10	31	0	0	0	0	0,025
0,8901	408,2	408,2	816,5	1,25	1	0,077	Equal	10	31	0	0	0	0	0,025
0,9308	476,3	476,3	952,6	1,25	1	0,077	Equal	10	31	0	0	0	0	0,025
0,9571	544,3	544,3	1088,6	1,25	1	0,077	Equal	10	31	0	0	0	0	0,025
0,9738	612,4	612,4	1224,7	1,25	1	0,077	Equal	10	31	0	0	0	0	0,025
0,9843	680,4	680,4	1360,8	1,25	1	0,077	Equal	10	31	0	0	0	0	0,025
0,8939	413,6	413,6	827,1	1,25	1	0,077	Equal	10	31	0	0	0	0	0,025
0,9337	482,5	482,5	965,0	1,25	1	0,077	Equal	10	31	0	0	0	0	0,025
0,9592	551,4	551,4	1102,8	1,25	1	0,077	Equal	10	31	0	0	0	0	0,025
0,9753	620,3	620,3	1240,7	1,25	1	0,077	Equal	10	31	0	0	0	0	0,025
0,9853	689,3	689,3	1378,5	1,25	1	0,077	Equal	10	31	0	0	0	0	0,025

* The reported proportions are during a single time period.

Non-Inferiority Logrank Tests

Power	The probability of rejecting a false null hypothesis when the alternative hypothesis is true.
E1, E2, E	The number of events in the reference group, the treatment group, and both groups, respectively.
HR0	The Non-Inferiority Hazard Ratio is the upper bound for the hazard ratio that still leads to the conclusion of non-inferiority.
HR1	The Actual Hazard Ratio is assumed to be the actual value of the hazard ratio. This is always set to 1.
h1	The Reference Group Hazard Rate is the hazard (instantaneous failure) rate of the reference group. Its scale is events per time period.
Accrual Pattern	The pattern of accrual used for each time period.
Accrual Time	The number of time periods (years or months) during which accrual takes place.
Total Time	The total number of time periods in the study. Follow-up time = (Total Time) - (Accrual Time).
Reference Proportion Lost	The proportion of the reference group that is lost (drops out) during a single time period (e.g., year or month).
Treatment Proportion Lost	The proportion of the treatment group that is lost (drops out) during a single time period (e.g., year or month).
Reference to Treatment	Drop-In. The proportion of the reference group that switch to a group with a hazard rate equal to the treatment group.
Treatment to Reference	Noncompliance. The proportion of the treatment group that switch to a group with a hazard rate equal to the reference group.
Alpha	The probability of rejecting a true null hypothesis.

Summary Statements

A parallel, two-group design (where higher hazard rates are considered worse) will be used to test whether the Group 2 (treatment) hazard rate is non-inferior to the Group 1 (control) hazard rate, with a non-inferiority hazard ratio of 1,25 (H0: HR ≥ 1,25 versus Ha: HR < 1,25, HR = h2 / h1). The comparison will be made using a one-sided, two-sample logrank test with a Type I error rate (α) of 0,025. The total duration of the study will be 15 time periods with subject accrual (entry) occurring in the first 6 time periods. The accrual pattern across time periods will be uniform (all periods equal). It is anticipated that no subjects will drop out of the control group. It is anticipated that no subjects will drop out of the treatment group. The proportion of subjects switching from the control group to another group with a hazard rate equal to the treatment group is 0 (per time period). The proportion of subjects switching from the treatment group to another group with a hazard rate equal to the control group is 0 (per time period). To detect a hazard ratio (h2 / h1) of 1 (h1 = 0,077, h2 = 0,077), with a sample size of 480 subjects in Group 1 and 480 subjects in Group 2 (totaling 960 subjects), the power is 0,7636. The corresponding number of events is 287,8 in Group 1 and 287,8 in Group 2 (totaling 575,6 events). The power was computed using PASS 2024, version 24.0.9.

References

Jung,Sin-Ho; Kang, Sun J.; McCall, Linda M.; Blumenstein, Brent. 2005. 'Sample Sizes Computation for Two-Sample Noninferiority Log-Rank Test', J. of Biopharmaceutical Statistics, Volume 15, pages 969-979.

Lakatos, Edward. 1988. 'Sample Sizes Based on the Log-Rank Statistic in Complex Clinical Trials', Biometrics, Volume 44, March, pages 229-241.

Non-Inferiority Logrank Tests

Citation

PASS 2024 Power Analysis and Sample Size Software (2024). NCSS, LLC. Kaysville, Utah, USA, ncss.com/software/pass.

PASS 2024, Version 24.0.9

Plots

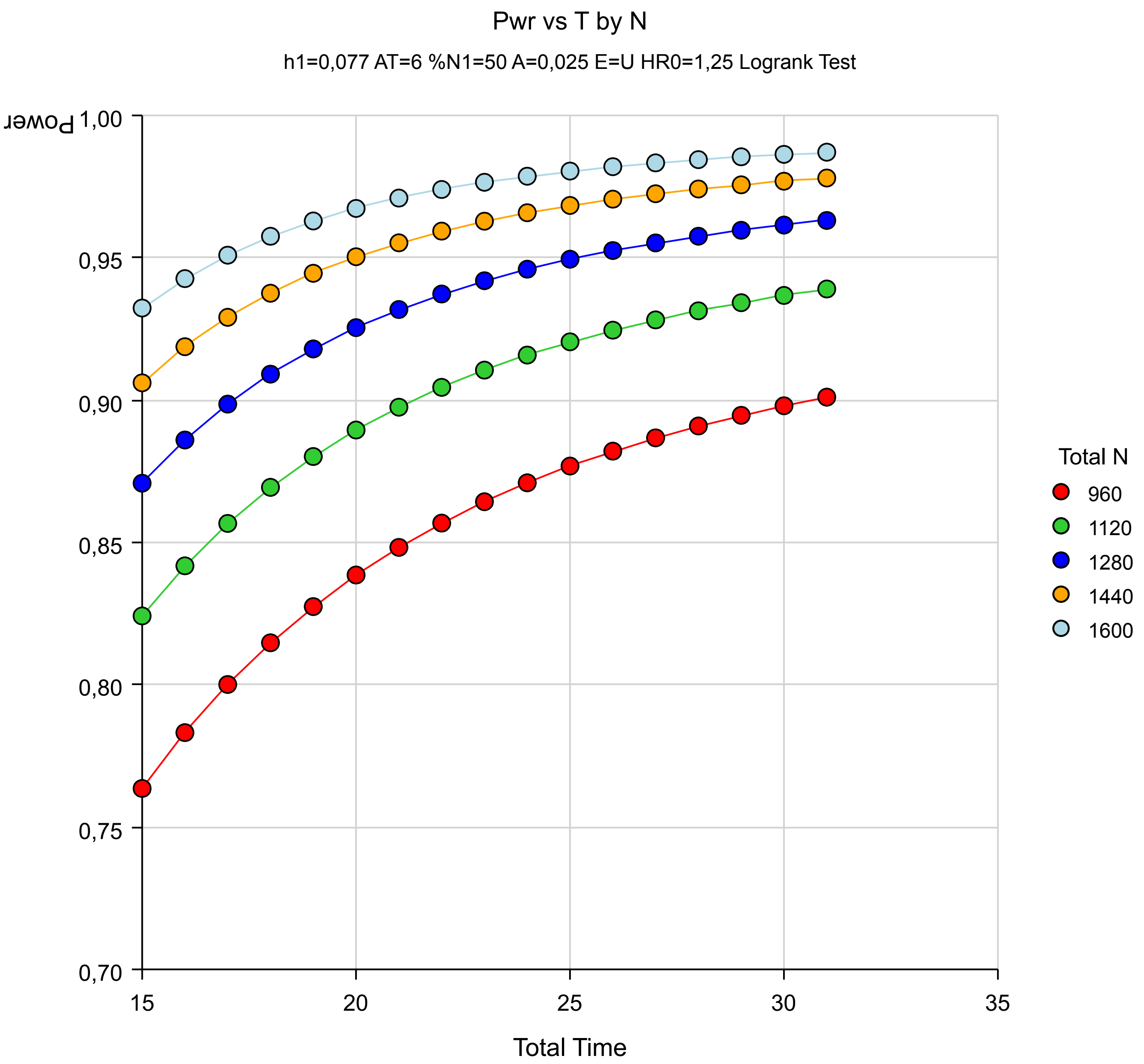

Pwr vs T by N
h1=0,077 AT=6 %N1=50 A=0,025 E=U HR0=1,25 Logrank Test
Power
1,00
0,95
0,90
0,85
0,80
0,75
0,70
15
20
25
30
35
Total Time
Total N
960
1120
1280
1440
1600

Non-Inferiority Logrank Tests

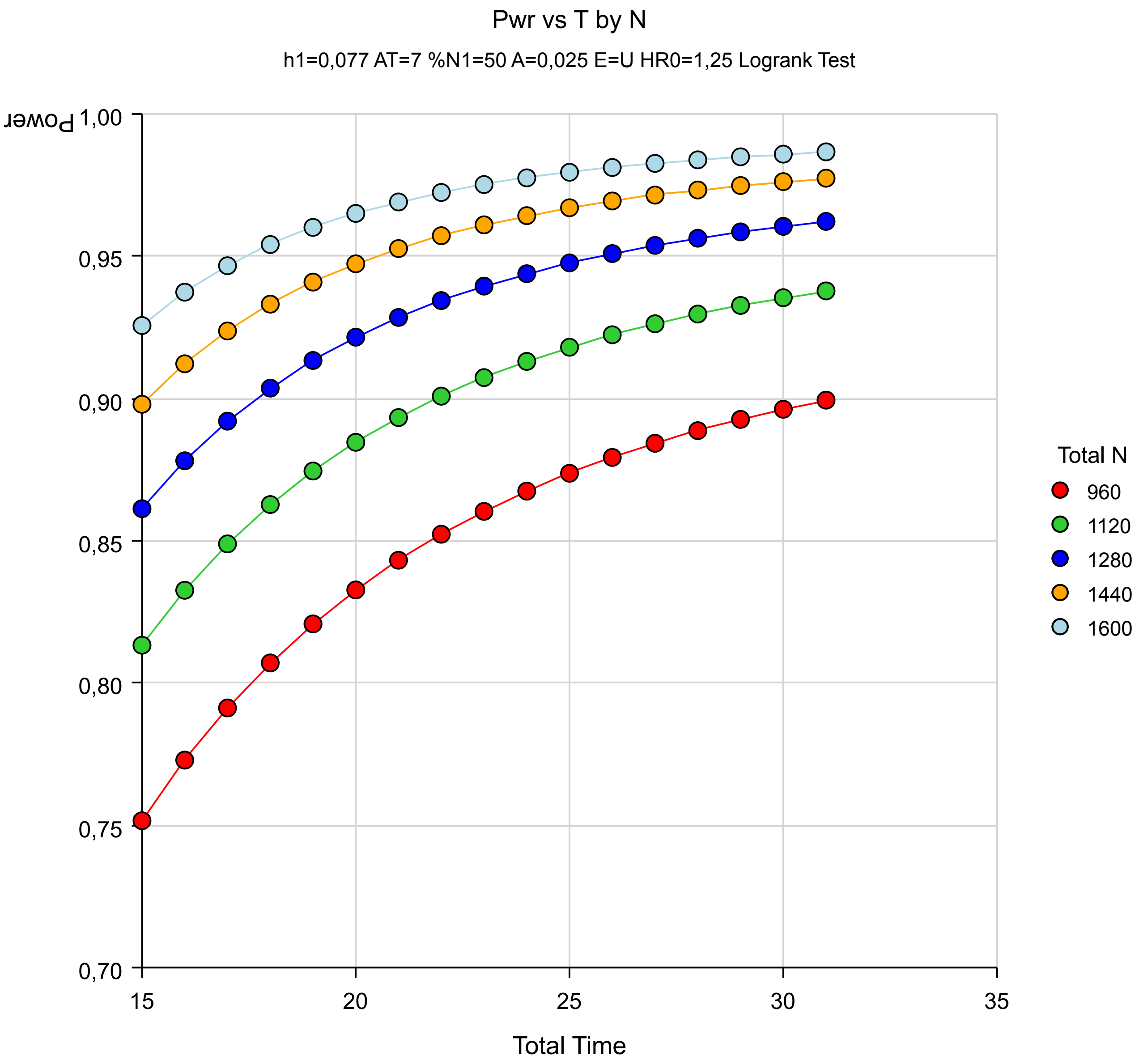

Non-Inferiority Logrank Tests

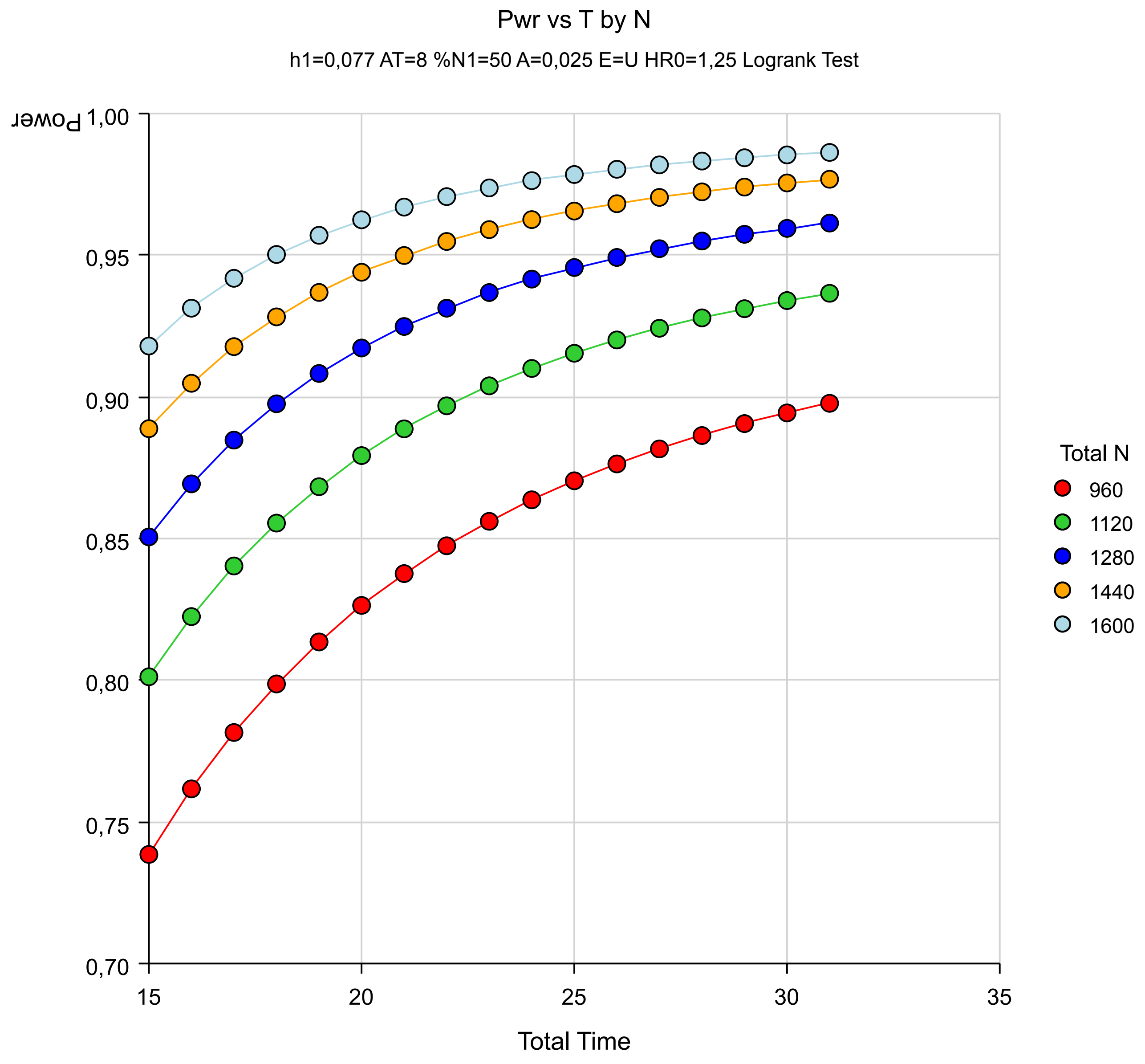

Non-Inferiority Logrank Tests

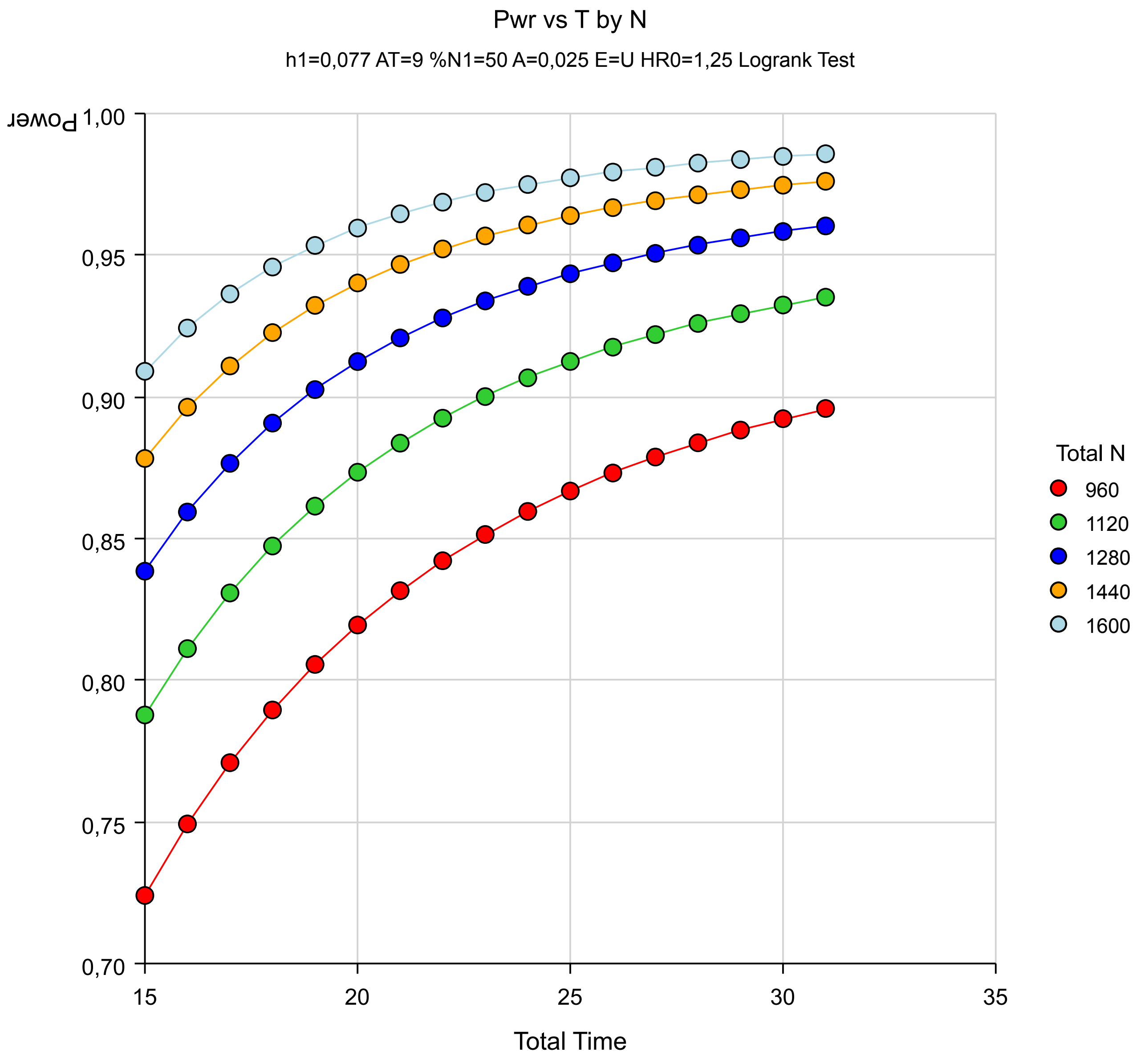

Non-Inferiority Logrank Tests

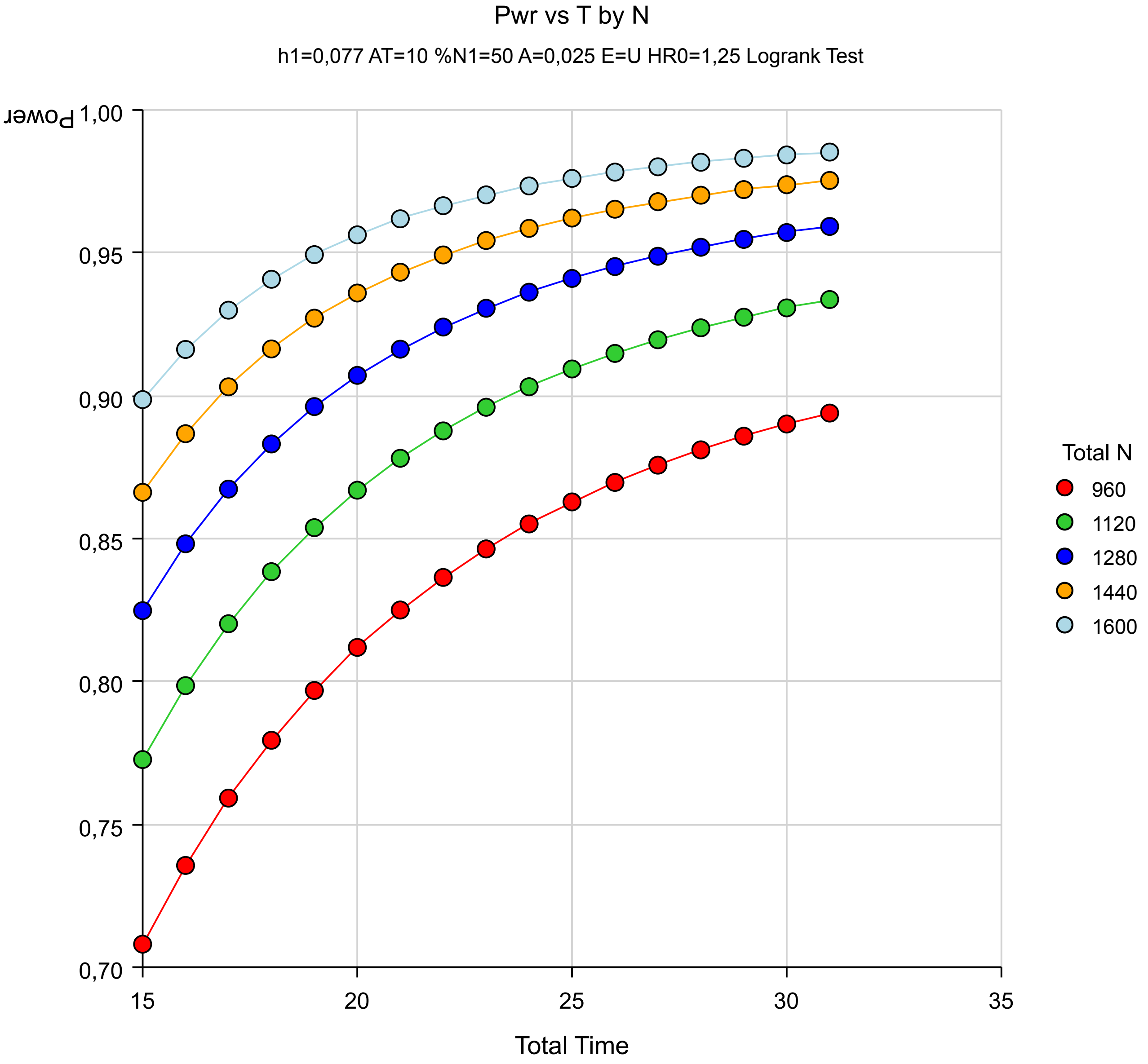

Non-Inferiority Logrank Tests

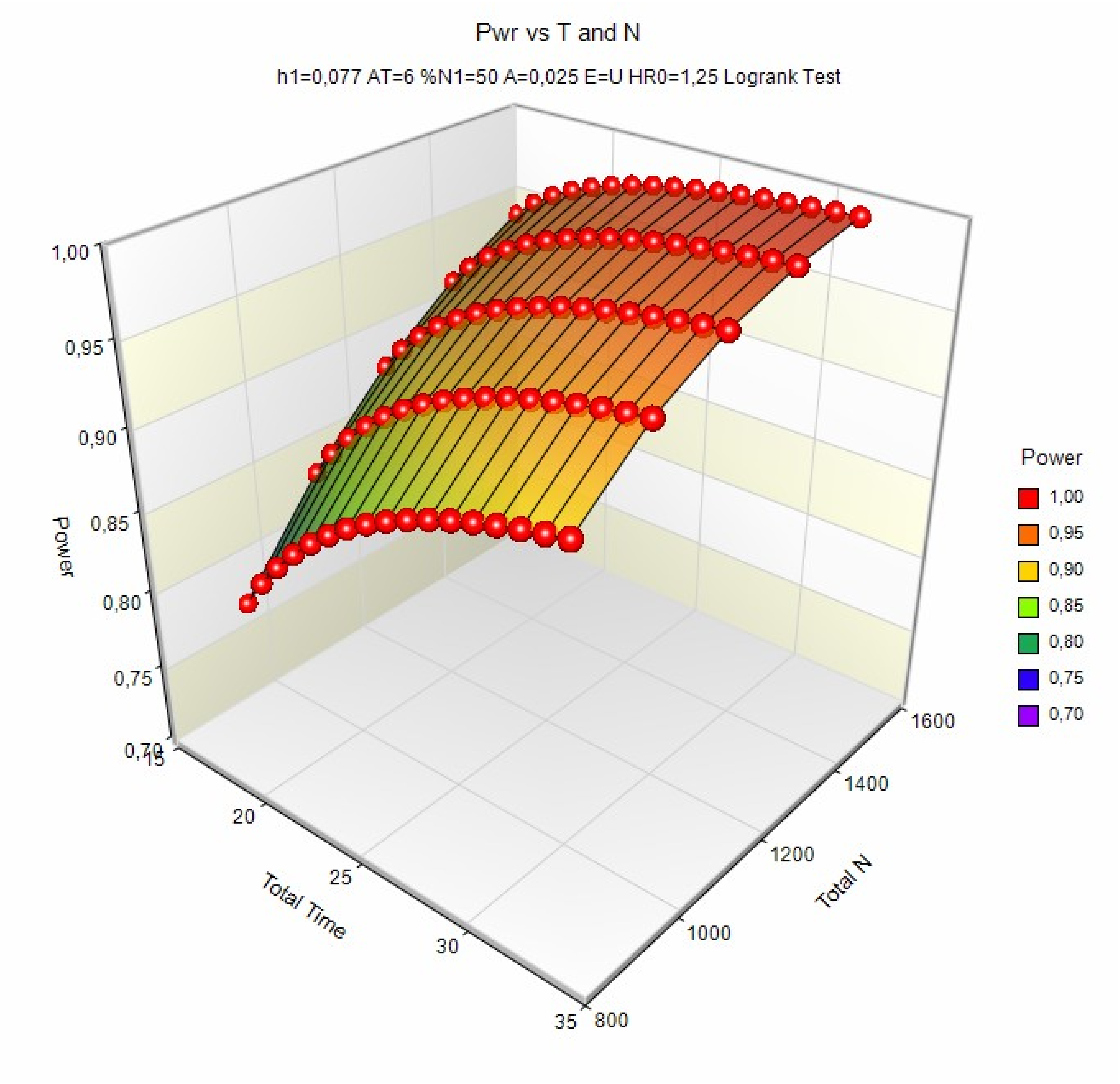

Non-Inferiority Logrank Tests

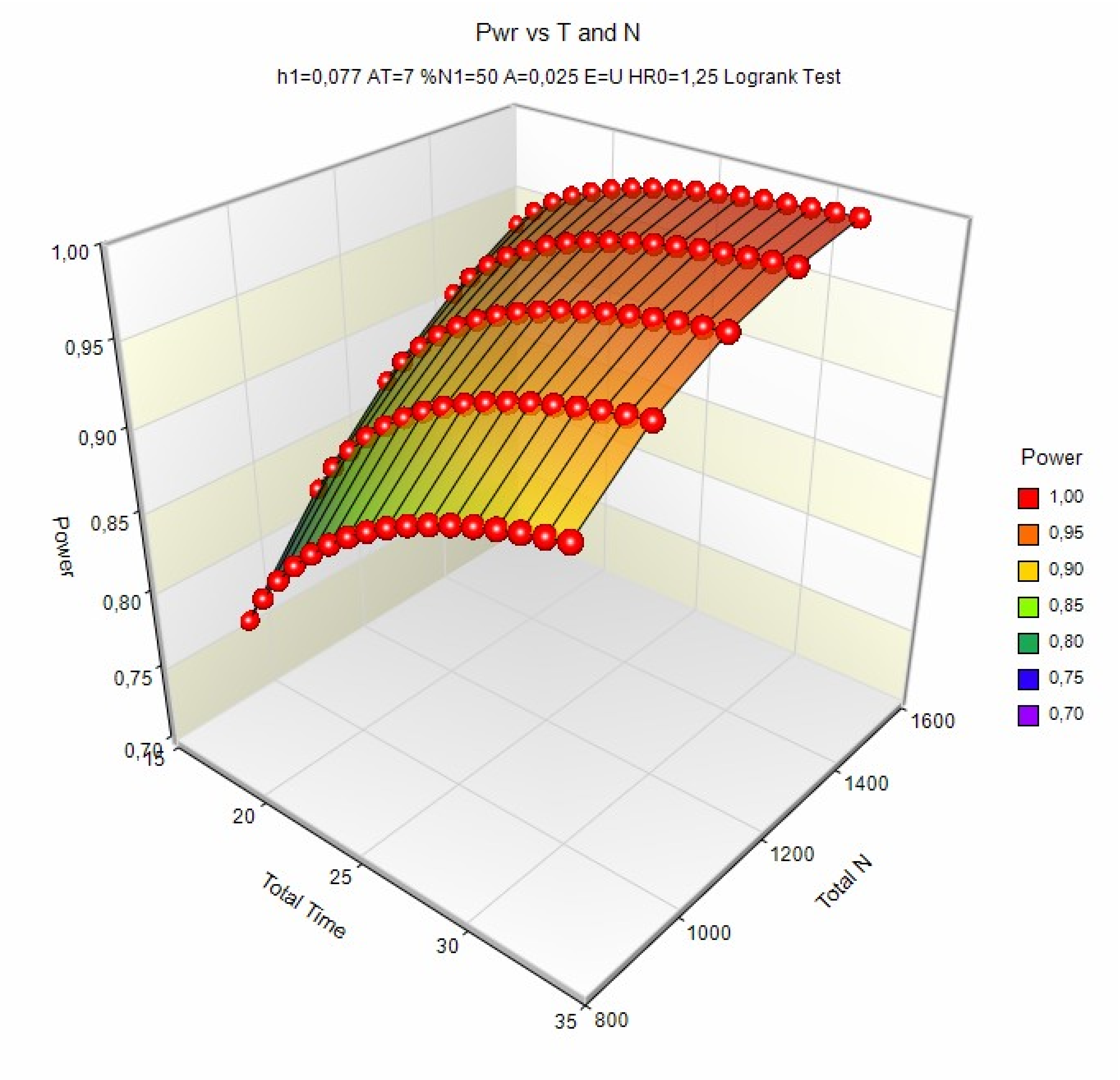

Non-Inferiority Logrank Tests

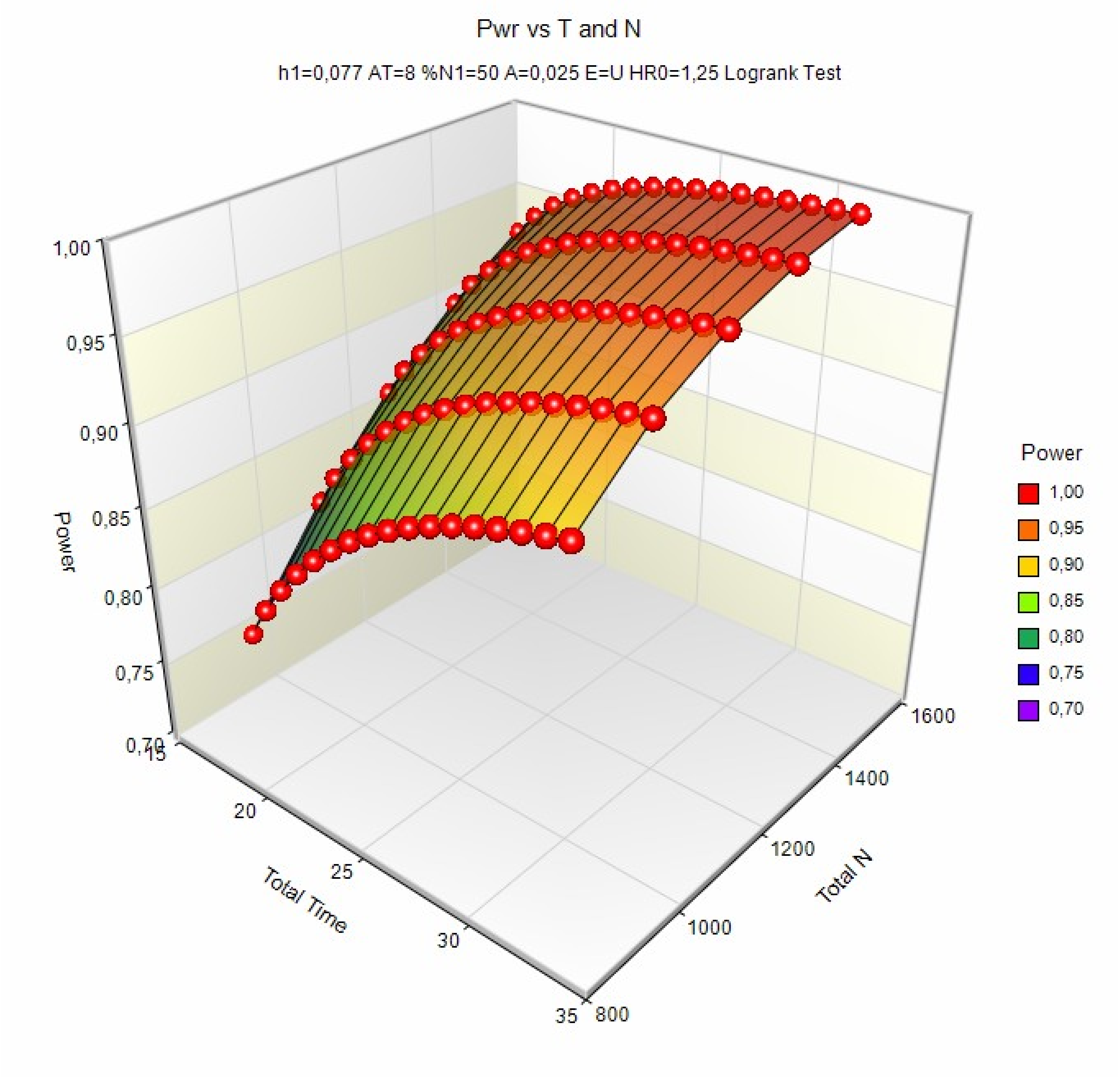

Non-Inferiority Logrank Tests

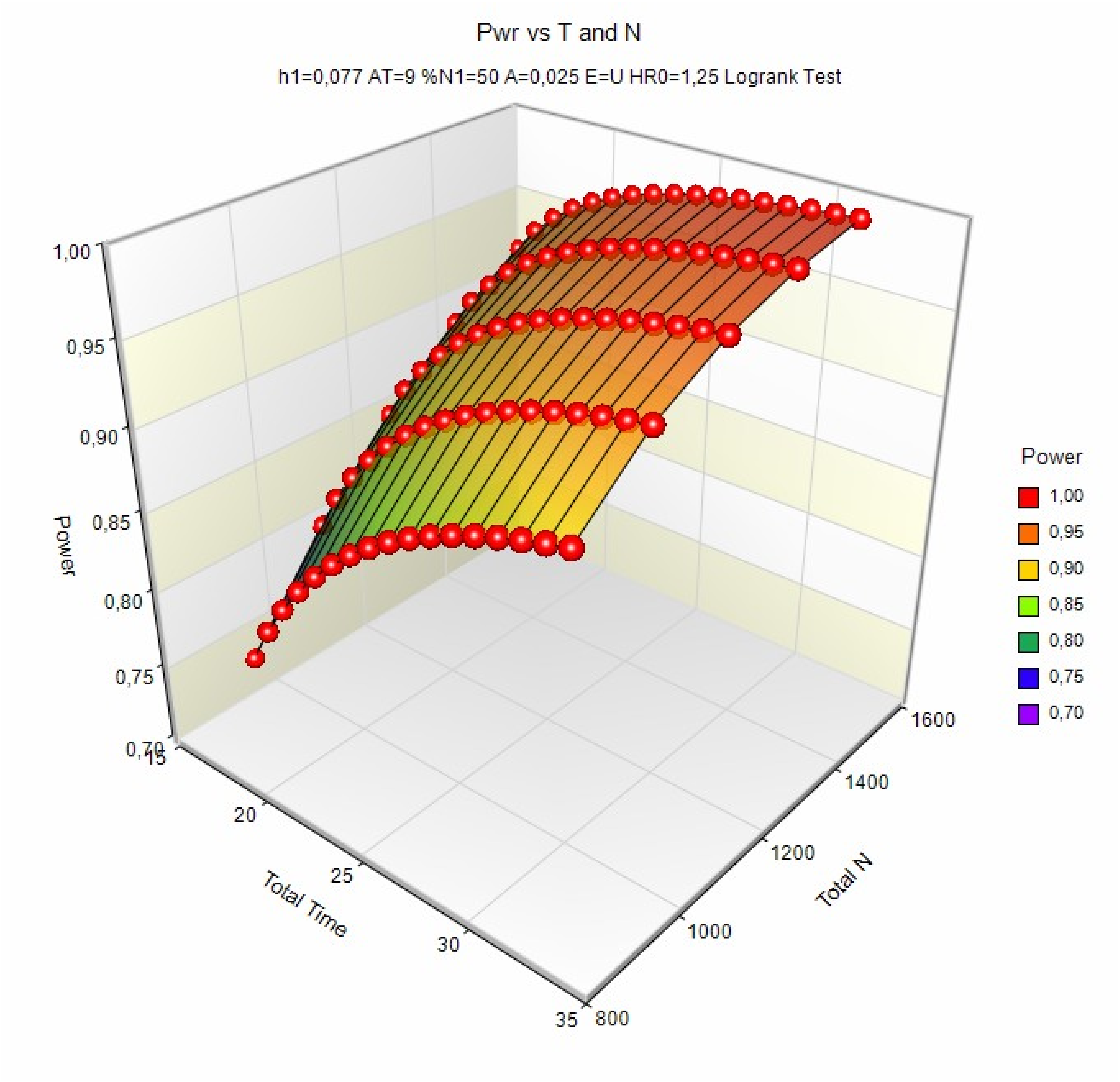

Non-Inferiority Logrank Tests

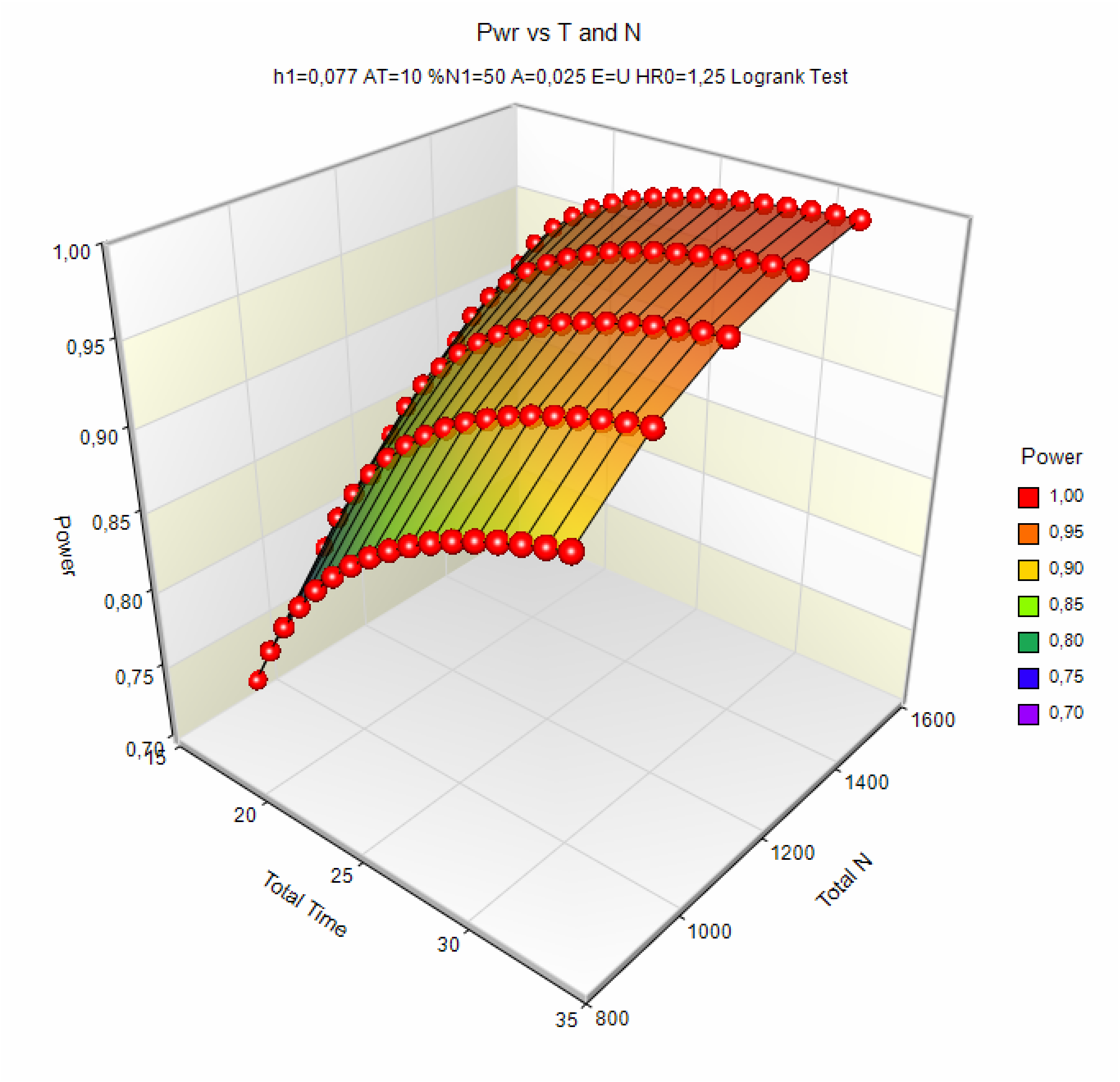

Non-Inferiority Logrank Tests

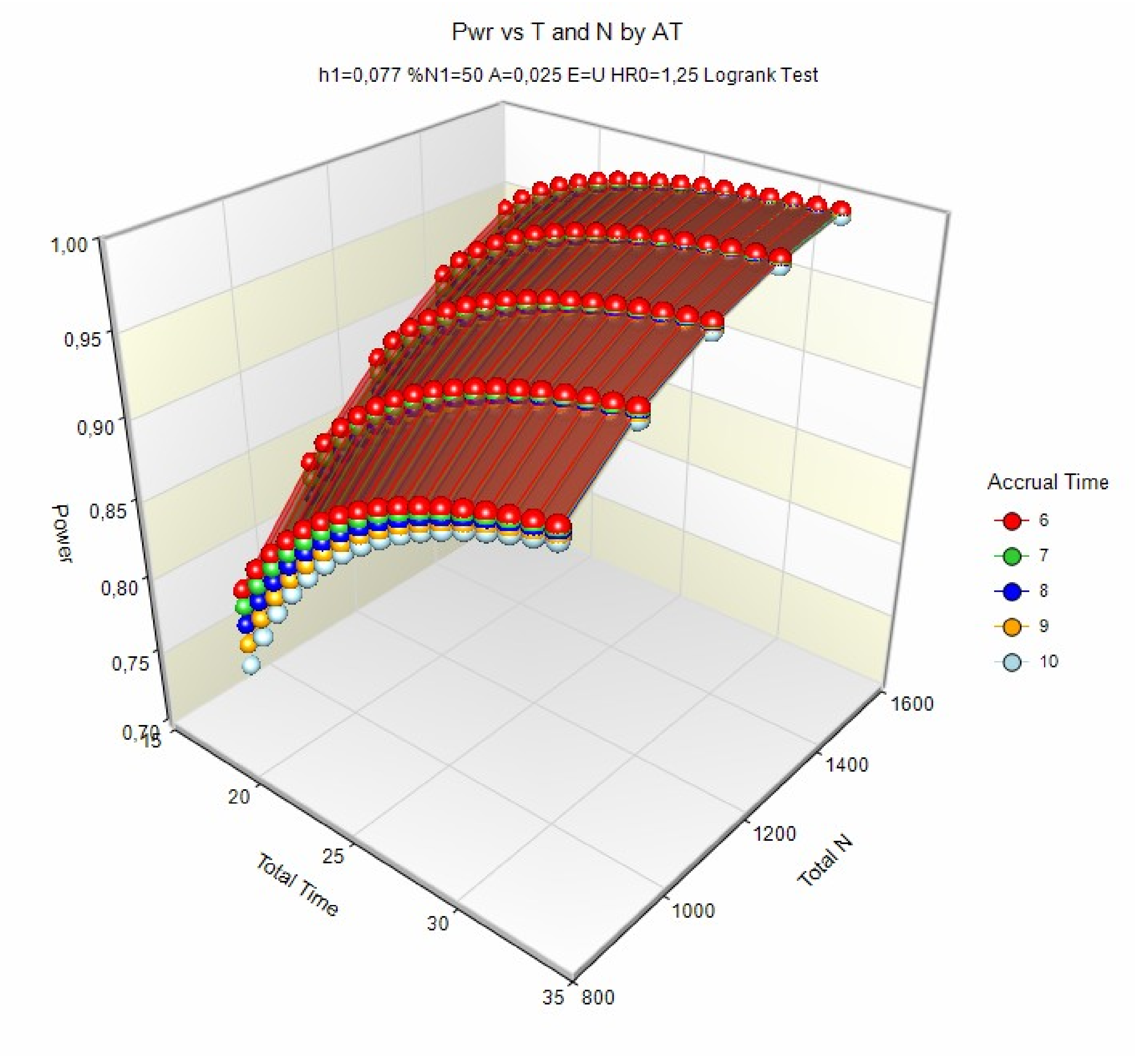

Non-Inferiority Logrank Tests

Procedure Input Settings

C:\Users\imbi\Documents\PASS 2024\Procedure Settings\Autosave\Non-Inferiority Logrank Tests - Autosaved 2026_9_3-10_44_53.t5

Design Tab

Solve For:	Power
Alpha:	0,025
Group Allocation:	Equal (N1 = N2)
Sample Size Per Group:	480 to 800 by 80
HR0 (Non-Inferiority Hazard Ratio):	1,25
h1 (Hazard Rate of Reference Group):	0,077
Accrual Time (Integers Only):	6 7 8 9 10
Accrual Pattern:	Uniform or Equal
Total Time (Integers Only):	15 to 31 by 1
References Lost:	0
References Switch to Treatment:	0,0
Treatments Lost:	0
Treatments Switch to Reference:	0,0

Options Tab

Number of Intervals within a Time Period:	2000
