# Supplementary material for: Current Management and the New Paradigm for ALK+ NSCLC After CROWN
Source: Cancers (Basel). 2026 Sep 19;18(18):3044. doi: 10.3390/cancers18183044 (PMC13605852; doi:10.3390/cancers18183044)
Supplement: Supplementary file 1 [file cancers-18-03044-s001.zip › File S3. Superiority calculation.pdf]

Logrank Tests

Numeric Results (Sample Size)

Solve For: Power
Test Type: Two-Sample Logrank Test
Groups: 1 = Control, 2 = Treatment
Alternative Hypothesis: Two-Sided

	Sample Size			Hazard Ratio	Median Survival Time		Accrual	Time		Proportion Lost*		Proportion Switching Groups*		
Power	N1	N2	N	HR	T1	T2	Pattern	Accrual	Total	Control	Treatment	Control to Treatment	Treatment to Control	Alpha
0,68455	320	320	640	0,75	9	12	Equal	4	11	0	0	0	0	0,05
0,77923	400	400	800	0,75	9	12	Equal	4	11	0	0	0	0	0,05
0,84873	480	480	960	0,75	9	12	Equal	4	11	0	0	0	0	0,05
0,89820	560	560	1120	0,75	9	12	Equal	4	11	0	0	0	0	0,05
0,93256	640	640	1280	0,75	9	12	Equal	4	11	0	0	0	0	0,05
0,71738	320	320	640	0,75	9	12	Equal	4	12	0	0	0	0	0,05
0,80928	400	400	800	0,75	9	12	Equal	4	12	0	0	0	0	0,05
0,87423	480	480	960	0,75	9	12	Equal	4	12	0	0	0	0	0,05
0,91867	560	560	1120	0,75	9	12	Equal	4	12	0	0	0	0	0,05
0,94829	640	640	1280	0,75	9	12	Equal	4	12	0	0	0	0	0,05
0,74549	320	320	640	0,75	9	12	Equal	4	13	0	0	0	0	0,05
0,83409	400	400	800	0,75	9	12	Equal	4	13	0	0	0	0	0,05
0,89450	480	480	960	0,75	9	12	Equal	4	13	0	0	0	0	0,05
0,93431	560	560	1120	0,75	9	12	Equal	4	13	0	0	0	0	0,05
0,95982	640	640	1280	0,75	9	12	Equal	4	13	0	0	0	0	0,05
0,76961	320	320	640	0,75	9	12	Equal	4	14	0	0	0	0	0,05
0,85467	400	400	800	0,75	9	12	Equal	4	14	0	0	0	0	0,05
0,91072	480	480	960	0,75	9	12	Equal	4	14	0	0	0	0	0,05
0,94635	560	560	1120	0,75	9	12	Equal	4	14	0	0	0	0	0,05
0,96838	640	640	1280	0,75	9	12	Equal	4	14	0	0	0	0	0,05
0,79037	320	320	640	0,75	9	12	Equal	4	15	0	0	0	0	0,05
0,87181	400	400	800	0,75	9	12	Equal	4	15	0	0	0	0	0,05
0,92377	480	480	960	0,75	9	12	Equal	4	15	0	0	0	0	0,05
0,95571	560	560	1120	0,75	9	12	Equal	4	15	0	0	0	0	0,05
0,97478	640	640	1280	0,75	9	12	Equal	4	15	0	0	0	0	0,05
0,80830	320	320	640	0,75	9	12	Equal	4	16	0	0	0	0	0,05
0,88616	400	400	800	0,75	9	12	Equal	4	16	0	0	0	0	0,05
0,93435	480	480	960	0,75	9	12	Equal	4	16	0	0	0	0	0,05

Logrank Tests

Numeric Results (Sample Size) (Continued)

Solve For: Power
Test Type: Two-Sample Logrank Test
Groups: 1 = Control, 2 = Treatment
Alternative Hypothesis: Two-Sided

	Sample Size			Hazard Ratio	Median Survival Time		Accrual	Time		Proportion Lost*		Proportion Switching Groups*		
Power	N1	N2	N	HR	T1	T2	Pattern	Accrual	Total	Control	Treatment	Control to Treatment	Treatment to Control	Alpha
0,96305	560	560	1120	0,75	9	12	Equal	4	16	0	0	0	0	0,05
0,97963	640	640	1280	0,75	9	12	Equal	4	16	0	0	0	0	0,05
0,82382	320	320	640	0,75	9	12	Equal	4	17	0	0	0	0	0,05
0,89823	400	400	800	0,75	9	12	Equal	4	17	0	0	0	0	0,05
0,94298	480	480	960	0,75	9	12	Equal	4	17	0	0	0	0	0,05
0,96885	560	560	1120	0,75	9	12	Equal	4	17	0	0	0	0	0,05
0,98335	640	640	1280	0,75	9	12	Equal	4	17	0	0	0	0	0,05
0,83732	320	320	640	0,75	9	12	Equal	4	18	0	0	0	0	0,05
0,90844	400	400	800	0,75	9	12	Equal	4	18	0	0	0	0	0,05
0,95007	480	480	960	0,75	9	12	Equal	4	18	0	0	0	0	0,05
0,97348	560	560	1120	0,75	9	12	Equal	4	18	0	0	0	0	0,05
0,98623	640	640	1280	0,75	9	12	Equal	4	18	0	0	0	0	0,05
0,84909	320	320	640	0,75	9	12	Equal	4	19	0	0	0	0	0,05
0,91712	400	400	800	0,75	9	12	Equal	4	19	0	0	0	0	0,05
0,95594	480	480	960	0,75	9	12	Equal	4	19	0	0	0	0	0,05
0,97721	560	560	1120	0,75	9	12	Equal	4	19	0	0	0	0	0,05
0,98848	640	640	1280	0,75	9	12	Equal	4	19	0	0	0	0	0,05
0,85939	320	320	640	0,75	9	12	Equal	4	20	0	0	0	0	0,05
0,92453	400	400	800	0,75	9	12	Equal	4	20	0	0	0	0	0,05
0,96083	480	480	960	0,75	9	12	Equal	4	20	0	0	0	0	0,05
0,98023	560	560	1120	0,75	9	12	Equal	4	20	0	0	0	0	0,05
0,99026	640	640	1280	0,75	9	12	Equal	4	20	0	0	0	0	0,05
0,86844	320	320	640	0,75	9	12	Equal	4	21	0	0	0	0	0,05
0,93089	400	400	800	0,75	9	12	Equal	4	21	0	0	0	0	0,05
0,96493	480	480	960	0,75	9	12	Equal	4	21	0	0	0	0	0,05
0,98271	560	560	1120	0,75	9	12	Equal	4	21	0	0	0	0	0,05
0,99168	640	640	1280	0,75	9	12	Equal	4	21	0	0	0	0	0,05
0,87641	320	320	640	0,75	9	12	Equal	4	22	0	0	0	0	0,05

Logrank Tests

Numeric Results (Sample Size) (Continued)

Solve For: Power
Test Type: Two-Sample Logrank Test
Groups: 1 = Control, 2 = Treatment
Alternative Hypothesis: Two-Sided

	Sample Size			Hazard Ratio	Median Survival Time			Time		Proportion Lost*		Proportion Switching Groups*		
Power	N1	N2	N	HR	T1	T2	Accrual Pattern	Accrual	Total	Control	Treatment	Control to Treatment	Treatment to Control	Alpha
0,93638	400	400	800	0,75	9	12	Equal	4	22	0	0	0	0	0,05
0,96839	480	480	960	0,75	9	12	Equal	4	22	0	0	0	0	0,05
0,98474	560	560	1120	0,75	9	12	Equal	4	22	0	0	0	0	0,05
0,99282	640	640	1280	0,75	9	12	Equal	4	22	0	0	0	0	0,05
0,88345	320	320	640	0,75	9	12	Equal	4	23	0	0	0	0	0,05
0,94113	400	400	800	0,75	9	12	Equal	4	23	0	0	0	0	0,05
0,97132	480	480	960	0,75	9	12	Equal	4	23	0	0	0	0	0,05
0,98644	560	560	1120	0,75	9	12	Equal	4	23	0	0	0	0	0,05
0,99375	640	640	1280	0,75	9	12	Equal	4	23	0	0	0	0	0,05
0,88970	320	320	640	0,75	9	12	Equal	4	24	0	0	0	0	0,05
0,94526	400	400	800	0,75	9	12	Equal	4	24	0	0	0	0	0,05
0,97382	480	480	960	0,75	9	12	Equal	4	24	0	0	0	0	0,05
0,98785	560	560	1120	0,75	9	12	Equal	4	24	0	0	0	0	0,05
0,99451	640	640	1280	0,75	9	12	Equal	4	24	0	0	0	0	0,05
0,89525	320	320	640	0,75	9	12	Equal	4	25	0	0	0	0	0,05
0,94887	400	400	800	0,75	9	12	Equal	4	25	0	0	0	0	0,05
0,97597	480	480	960	0,75	9	12	Equal	4	25	0	0	0	0	0,05
0,98905	560	560	1120	0,75	9	12	Equal	4	25	0	0	0	0	0,05
0,99514	640	640	1280	0,75	9	12	Equal	4	25	0	0	0	0	0,05
0,90019	320	320	640	0,75	9	12	Equal	4	26	0	0	0	0	0,05
0,95204	400	400	800	0,75	9	12	Equal	4	26	0	0	0	0	0,05
0,97782	480	480	960	0,75	9	12	Equal	4	26	0	0	0	0	0,05
0,99006	560	560	1120	0,75	9	12	Equal	4	26	0	0	0	0	0,05
0,99566	640	640	1280	0,75	9	12	Equal	4	26	0	0	0	0	0,05
0,66514	320	320	640	0,75	9	12	Equal	5	11	0	0	0	0	0,05
0,76095	400	400	800	0,75	9	12	Equal	5	11	0	0	0	0	0,05
0,83274	480	480	960	0,75	9	12	Equal	5	11	0	0	0	0	0,05
0,88496	560	560	1120	0,75	9	12	Equal	5	11	0	0	0	0	0,05

Logrank Tests

Numeric Results (Sample Size) (Continued)

Solve For: Power
Test Type: Two-Sample Logrank Test
Groups: 1 = Control, 2 = Treatment
Alternative Hypothesis: Two-Sided

	Sample Size			Hazard Ratio	Median Survival Time			Time		Proportion Lost*		Proportion Switching Groups*		
Power	N1	N2	N	HR	T1	T2	Accrual Pattern	Accrual	Total	Control	Treatment	Control to Treatment	Treatment to Control	Alpha
0,92206	640	640	1280	0,75	9	12	Equal	5	11	0	0	0	0	0,05
0,70078	320	320	640	0,75	9	12	Equal	5	12	0	0	0	0	0,05
0,79422	400	400	800	0,75	9	12	Equal	5	12	0	0	0	0	0,05
0,86157	480	480	960	0,75	9	12	Equal	5	12	0	0	0	0	0,05
0,90861	560	560	1120	0,75	9	12	Equal	5	12	0	0	0	0	0,05
0,94064	640	640	1280	0,75	9	12	Equal	5	12	0	0	0	0	0,05
0,73126	320	320	640	0,75	9	12	Equal	5	13	0	0	0	0	0,05
0,82164	400	400	800	0,75	9	12	Equal	5	13	0	0	0	0	0,05
0,88442	480	480	960	0,75	9	12	Equal	5	13	0	0	0	0	0,05
0,92661	560	560	1120	0,75	9	12	Equal	5	13	0	0	0	0	0,05
0,95420	640	640	1280	0,75	9	12	Equal	5	13	0	0	0	0	0,05
0,75739	320	320	640	0,75	9	12	Equal	5	14	0	0	0	0	0,05
0,84433	400	400	800	0,75	9	12	Equal	5	14	0	0	0	0	0,05
0,90264	480	480	960	0,75	9	12	Equal	5	14	0	0	0	0	0,05
0,94041	560	560	1120	0,75	9	12	Equal	5	14	0	0	0	0	0,05
0,96419	640	640	1280	0,75	9	12	Equal	5	14	0	0	0	0	0,05
0,77985	320	320	640	0,75	9	12	Equal	5	15	0	0	0	0	0,05
0,86319	400	400	800	0,75	9	12	Equal	5	15	0	0	0	0	0,05
0,91726	480	480	960	0,75	9	12	Equal	5	15	0	0	0	0	0,05
0,95108	560	560	1120	0,75	9	12	Equal	5	15	0	0	0	0	0,05
0,97164	640	640	1280	0,75	9	12	Equal	5	15	0	0	0	0	0,05
0,79920	320	320	640	0,75	9	12	Equal	5	16	0	0	0	0	0,05
0,87893	400	400	800	0,75	9	12	Equal	5	16	0	0	0	0	0,05
0,92906	480	480	960	0,75	9	12	Equal	5	16	0	0	0	0	0,05
0,95941	560	560	1120	0,75	9	12	Equal	5	16	0	0	0	0	0,05
0,97725	640	640	1280	0,75	9	12	Equal	5	16	0	0	0	0	0,05
0,81594	320	320	640	0,75	9	12	Equal	5	17	0	0	0	0	0,05
0,89214	400	400	800	0,75	9	12	Equal	5	17	0	0	0	0	0,05

Logrank Tests

Numeric Results (Sample Size) (Continued)

Solve For: Power
Test Type: Two-Sample Logrank Test
Groups: 1 = Control, 2 = Treatment
Alternative Hypothesis: Two-Sided

	Sample Size			Hazard Ratio	Median Survival Time		Accrual	Time		Proportion Lost*		Proportion Switching Groups*		
Power	N1	N2	N	HR	T1	T2	Pattern	Accrual	Total	Control	Treatment	Control to Treatment	Treatment to Control	Alpha
0,93866	480	480	960	0,75	9	12	Equal	5	17	0	0	0	0	0,05
0,96597	560	560	1120	0,75	9	12	Equal	5	17	0	0	0	0	0,05
0,98152	640	640	1280	0,75	9	12	Equal	5	17	0	0	0	0	0,05
0,83046	320	320	640	0,75	9	12	Equal	5	18	0	0	0	0	0,05
0,90328	400	400	800	0,75	9	12	Equal	5	18	0	0	0	0	0,05
0,94651	480	480	960	0,75	9	12	Equal	5	18	0	0	0	0	0,05
0,97118	560	560	1120	0,75	9	12	Equal	5	18	0	0	0	0	0,05
0,98481	640	640	1280	0,75	9	12	Equal	5	18	0	0	0	0	0,05
0,84310	320	320	640	0,75	9	12	Equal	5	19	0	0	0	0	0,05
0,91273	400	400	800	0,75	9	12	Equal	5	19	0	0	0	0	0,05
0,95299	480	480	960	0,75	9	12	Equal	5	19	0	0	0	0	0,05
0,97535	560	560	1120	0,75	9	12	Equal	5	19	0	0	0	0	0,05
0,98736	640	640	1280	0,75	9	12	Equal	5	19	0	0	0	0	0,05
0,85415	320	320	640	0,75	9	12	Equal	5	20	0	0	0	0	0,05
0,92078	400	400	800	0,75	9	12	Equal	5	20	0	0	0	0	0,05
0,95837	480	480	960	0,75	9	12	Equal	5	20	0	0	0	0	0,05
0,97872	560	560	1120	0,75	9	12	Equal	5	20	0	0	0	0	0,05
0,98937	640	640	1280	0,75	9	12	Equal	5	20	0	0	0	0	0,05
0,86383	320	320	640	0,75	9	12	Equal	5	21	0	0	0	0	0,05
0,92767	400	400	800	0,75	9	12	Equal	5	21	0	0	0	0	0,05
0,96286	480	480	960	0,75	9	12	Equal	5	21	0	0	0	0	0,05
0,98147	560	560	1120	0,75	9	12	Equal	5	21	0	0	0	0	0,05
0,99097	640	640	1280	0,75	9	12	Equal	5	21	0	0	0	0	0,05
0,87234	320	320	640	0,75	9	12	Equal	5	22	0	0	0	0	0,05
0,93359	400	400	800	0,75	9	12	Equal	5	22	0	0	0	0	0,05
0,96664	480	480	960	0,75	9	12	Equal	5	22	0	0	0	0	0,05
0,98372	560	560	1120	0,75	9	12	Equal	5	22	0	0	0	0	0,05
0,99225	640	640	1280	0,75	9	12	Equal	5	22	0	0	0	0	0,05

Logrank Tests

Numeric Results (Sample Size) (Continued)

Solve For: Power
Test Type: Two-Sample Logrank Test
Groups: 1 = Control, 2 = Treatment
Alternative Hypothesis: Two-Sided

	Sample Size			Hazard Ratio	Median Survival Time			Time		Proportion Lost*		Proportion Switching Groups*		
Power	N1	N2	N	HR	T1	T2	Accrual Pattern	Accrual	Total	Control	Treatment	Control to Treatment	Treatment to Control	Alpha
0,87986	320	320	640	0,75	9	12	Equal	5	23	0	0	0	0	0,05
0,93871	400	400	800	0,75	9	12	Equal	5	23	0	0	0	0	0,05
0,96984	480	480	960	0,75	9	12	Equal	5	23	0	0	0	0	0,05
0,98559	560	560	1120	0,75	9	12	Equal	5	23	0	0	0	0	0,05
0,99328	640	640	1280	0,75	9	12	Equal	5	23	0	0	0	0	0,05
0,88651	320	320	640	0,75	9	12	Equal	5	24	0	0	0	0	0,05
0,94316	400	400	800	0,75	9	12	Equal	5	24	0	0	0	0	0,05
0,97255	480	480	960	0,75	9	12	Equal	5	24	0	0	0	0	0,05
0,98714	560	560	1120	0,75	9	12	Equal	5	24	0	0	0	0	0,05
0,99413	640	640	1280	0,75	9	12	Equal	5	24	0	0	0	0	0,05
0,89241	320	320	640	0,75	9	12	Equal	5	25	0	0	0	0	0,05
0,94703	400	400	800	0,75	9	12	Equal	5	25	0	0	0	0	0,05
0,97488	480	480	960	0,75	9	12	Equal	5	25	0	0	0	0	0,05
0,98844	560	560	1120	0,75	9	12	Equal	5	25	0	0	0	0	0,05
0,99482	640	640	1280	0,75	9	12	Equal	5	25	0	0	0	0	0,05
0,89766	320	320	640	0,75	9	12	Equal	5	26	0	0	0	0	0,05
0,95043	400	400	800	0,75	9	12	Equal	5	26	0	0	0	0	0,05
0,97688	480	480	960	0,75	9	12	Equal	5	26	0	0	0	0	0,05
0,98954	560	560	1120	0,75	9	12	Equal	5	26	0	0	0	0	0,05
0,99539	640	640	1280	0,75	9	12	Equal	5	26	0	0	0	0	0,05
0,64383	320	320	640	0,75	9	12	Equal	6	11	0	0	0	0	0,05
0,74045	400	400	800	0,75	9	12	Equal	6	11	0	0	0	0	0,05
0,81442	480	480	960	0,75	9	12	Equal	6	11	0	0	0	0	0,05
0,86945	560	560	1120	0,75	9	12	Equal	6	11	0	0	0	0	0,05
0,90946	640	640	1280	0,75	9	12	Equal	6	11	0	0	0	0	0,05
0,68256	320	320	640	0,75	9	12	Equal	6	12	0	0	0	0	0,05
0,77736	400	400	800	0,75	9	12	Equal	6	12	0	0	0	0	0,05
0,84711	480	480	960	0,75	9	12	Equal	6	12	0	0	0	0	0,05

Logrank Tests

Numeric Results (Sample Size) (Continued)

Solve For: Power
Test Type: Two-Sample Logrank Test
Groups: 1 = Control, 2 = Treatment
Alternative Hypothesis: Two-Sided

	Sample Size			Hazard Ratio	Median Survival Time			Time		Proportion Lost*		Proportion Switching Groups*		
Power	N1	N2	N	HR	T1	T2	Accrual Pattern	Accrual	Total	Control	Treatment	Control to Treatment	Treatment to Control	Alpha
0,89687	560	560	1120	0,75	9	12	Equal	6	12	0	0	0	0	0,05
0,93151	640	640	1280	0,75	9	12	Equal	6	12	0	0	0	0	0,05
0,71567	320	320	640	0,75	9	12	Equal	6	13	0	0	0	0	0,05
0,80774	400	400	800	0,75	9	12	Equal	6	13	0	0	0	0	0,05
0,87294	480	480	960	0,75	9	12	Equal	6	13	0	0	0	0	0,05
0,91766	560	560	1120	0,75	9	12	Equal	6	13	0	0	0	0	0,05
0,94752	640	640	1280	0,75	9	12	Equal	6	13	0	0	0	0	0,05
0,74401	320	320	640	0,75	9	12	Equal	6	14	0	0	0	0	0,05
0,83281	400	400	800	0,75	9	12	Equal	6	14	0	0	0	0	0,05
0,89347	480	480	960	0,75	9	12	Equal	6	14	0	0	0	0	0,05
0,93353	560	560	1120	0,75	9	12	Equal	6	14	0	0	0	0	0,05
0,95926	640	640	1280	0,75	9	12	Equal	6	14	0	0	0	0	0,05
0,76834	320	320	640	0,75	9	12	Equal	6	15	0	0	0	0	0,05
0,85360	400	400	800	0,75	9	12	Equal	6	15	0	0	0	0	0,05
0,90989	480	480	960	0,75	9	12	Equal	6	15	0	0	0	0	0,05
0,94575	560	560	1120	0,75	9	12	Equal	6	15	0	0	0	0	0,05
0,96795	640	640	1280	0,75	9	12	Equal	6	15	0	0	0	0	0,05
0,78928	320	320	640	0,75	9	12	Equal	6	16	0	0	0	0	0,05
0,87092	400	400	800	0,75	9	12	Equal	6	16	0	0	0	0	0,05
0,92310	480	480	960	0,75	9	12	Equal	6	16	0	0	0	0	0,05
0,95524	560	560	1120	0,75	9	12	Equal	6	16	0	0	0	0	0,05
0,97446	640	640	1280	0,75	9	12	Equal	6	16	0	0	0	0	0,05
0,80735	320	320	640	0,75	9	12	Equal	6	17	0	0	0	0	0,05
0,88541	400	400	800	0,75	9	12	Equal	6	17	0	0	0	0	0,05
0,93380	480	480	960	0,75	9	12	Equal	6	17	0	0	0	0	0,05
0,96268	560	560	1120	0,75	9	12	Equal	6	17	0	0	0	0	0,05
0,97939	640	640	1280	0,75	9	12	Equal	6	17	0	0	0	0	0,05
0,82300	320	320	640	0,75	9	12	Equal	6	18	0	0	0	0	0,05

Logrank Tests

Numeric Results (Sample Size) (Continued)

Solve For: Power
Test Type: Two-Sample Logrank Test
Groups: 1 = Control, 2 = Treatment
Alternative Hypothesis: Two-Sided

	Sample Size			Hazard Ratio	Median Survival Time		Accrual	Time		Proportion Lost*		Proportion Switching Groups*		
Power	N1	N2	N	HR	T1	T2	Pattern	Accrual	Total	Control	Treatment	Control to Treatment	Treatment to Control	Alpha
0,89760	400	400	800	0,75	9	12	Equal	6	18	0	0	0	0	0,05
0,94253	480	480	960	0,75	9	12	Equal	6	18	0	0	0	0	0,05
0,96856	560	560	1120	0,75	9	12	Equal	6	18	0	0	0	0	0,05
0,98316	640	640	1280	0,75	9	12	Equal	6	18	0	0	0	0	0,05
0,83660	320	320	640	0,75	9	12	Equal	6	19	0	0	0	0	0,05
0,90790	400	400	800	0,75	9	12	Equal	6	19	0	0	0	0	0,05
0,94970	480	480	960	0,75	9	12	Equal	6	19	0	0	0	0	0,05
0,97324	560	560	1120	0,75	9	12	Equal	6	19	0	0	0	0	0,05
0,98608	640	640	1280	0,75	9	12	Equal	6	19	0	0	0	0	0,05
0,84846	320	320	640	0,75	9	12	Equal	6	20	0	0	0	0	0,05
0,91666	400	400	800	0,75	9	12	Equal	6	20	0	0	0	0	0,05
0,95564	480	480	960	0,75	9	12	Equal	6	20	0	0	0	0	0,05
0,97702	560	560	1120	0,75	9	12	Equal	6	20	0	0	0	0	0,05
0,98836	640	640	1280	0,75	9	12	Equal	6	20	0	0	0	0	0,05
0,85884	320	320	640	0,75	9	12	Equal	6	21	0	0	0	0	0,05
0,92414	400	400	800	0,75	9	12	Equal	6	21	0	0	0	0	0,05
0,96058	480	480	960	0,75	9	12	Equal	6	21	0	0	0	0	0,05
0,98008	560	560	1120	0,75	9	12	Equal	6	21	0	0	0	0	0,05
0,99017	640	640	1280	0,75	9	12	Equal	6	21	0	0	0	0	0,05
0,86795	320	320	640	0,75	9	12	Equal	6	22	0	0	0	0	0,05
0,93055	400	400	800	0,75	9	12	Equal	6	22	0	0	0	0	0,05
0,96471	480	480	960	0,75	9	12	Equal	6	22	0	0	0	0	0,05
0,98258	560	560	1120	0,75	9	12	Equal	6	22	0	0	0	0	0,05
0,99160	640	640	1280	0,75	9	12	Equal	6	22	0	0	0	0	0,05
0,87598	320	320	640	0,75	9	12	Equal	6	23	0	0	0	0	0,05
0,93608	400	400	800	0,75	9	12	Equal	6	23	0	0	0	0	0,05
0,96820	480	480	960	0,75	9	12	Equal	6	23	0	0	0	0	0,05
0,98464	560	560	1120	0,75	9	12	Equal	6	23	0	0	0	0	0,05

Logrank Tests

Numeric Results (Sample Size) (Continued)

Solve For: Power
Test Type: Two-Sample Logrank Test
Groups: 1 = Control, 2 = Treatment
Alternative Hypothesis: Two-Sided

	Sample Size			Hazard Ratio	Median Survival Time			Time		Proportion Lost*		Proportion Switching Groups*		
Power	N1	N2	N	HR	T1	T2	Accrual Pattern	Accrual	Total	Control	Treatment	Control to Treatment	Treatment to Control	Alpha
0,99276	640	640	1280	0,75	9	12	Equal	6	23	0	0	0	0	0,05
0,88307	320	320	640	0,75	9	12	Equal	6	24	0	0	0	0	0,05
0,94087	400	400	800	0,75	9	12	Equal	6	24	0	0	0	0	0,05
0,97116	480	480	960	0,75	9	12	Equal	6	24	0	0	0	0	0,05
0,98635	560	560	1120	0,75	9	12	Equal	6	24	0	0	0	0	0,05
0,99370	640	640	1280	0,75	9	12	Equal	6	24	0	0	0	0	0,05
0,88936	320	320	640	0,75	9	12	Equal	6	25	0	0	0	0	0,05
0,94504	400	400	800	0,75	9	12	Equal	6	25	0	0	0	0	0,05
0,97369	480	480	960	0,75	9	12	Equal	6	25	0	0	0	0	0,05
0,98778	560	560	1120	0,75	9	12	Equal	6	25	0	0	0	0	0,05
0,99447	640	640	1280	0,75	9	12	Equal	6	25	0	0	0	0	0,05
0,89494	320	320	640	0,75	9	12	Equal	6	26	0	0	0	0	0,05
0,94868	400	400	800	0,75	9	12	Equal	6	26	0	0	0	0	0,05
0,97585	480	480	960	0,75	9	12	Equal	6	26	0	0	0	0	0,05
0,98898	560	560	1120	0,75	9	12	Equal	6	26	0	0	0	0	0,05
0,99510	640	640	1280	0,75	9	12	Equal	6	26	0	0	0	0	0,05
0,62041	320	320	640	0,75	9	12	Equal	7	11	0	0	0	0	0,05
0,71745	400	400	800	0,75	9	12	Equal	7	11	0	0	0	0	0,05
0,79340	480	480	960	0,75	9	12	Equal	7	11	0	0	0	0	0,05
0,85123	560	560	1120	0,75	9	12	Equal	7	11	0	0	0	0	0,05
0,89429	640	640	1280	0,75	9	12	Equal	7	11	0	0	0	0	0,05
0,66254	320	320	640	0,75	9	12	Equal	7	12	0	0	0	0	0,05
0,75846	400	400	800	0,75	9	12	Equal	7	12	0	0	0	0	0,05
0,83054	480	480	960	0,75	9	12	Equal	7	12	0	0	0	0	0,05
0,88311	560	560	1120	0,75	9	12	Equal	7	12	0	0	0	0	0,05
0,92057	640	640	1280	0,75	9	12	Equal	7	12	0	0	0	0	0,05
0,69855	320	320	640	0,75	9	12	Equal	7	13	0	0	0	0	0,05
0,79217	400	400	800	0,75	9	12	Equal	7	13	0	0	0	0	0,05

Logrank Tests

Numeric Results (Sample Size) (Continued)

Solve For: Power
Test Type: Two-Sample Logrank Test
Groups: 1 = Control, 2 = Treatment
Alternative Hypothesis: Two-Sided

	Sample Size			Hazard Ratio	Median Survival Time		Accrual	Time		Proportion Lost*		Proportion Switching Groups*		
Power	N1	N2	N	HR	T1	T2	Pattern	Accrual	Total	Control	Treatment	Control to Treatment	Treatment to Control	Alpha
0,85983	480	480	960	0,75	9	12	Equal	7	13	0	0	0	0	0,05
0,90721	560	560	1120	0,75	9	12	Equal	7	13	0	0	0	0	0,05
0,93956	640	640	1280	0,75	9	12	Equal	7	13	0	0	0	0	0,05
0,72935	320	320	640	0,75	9	12	Equal	7	14	0	0	0	0	0,05
0,81995	400	400	800	0,75	9	12	Equal	7	14	0	0	0	0	0,05
0,88303	480	480	960	0,75	9	12	Equal	7	14	0	0	0	0	0,05
0,92553	560	560	1120	0,75	9	12	Equal	7	14	0	0	0	0	0,05
0,95340	640	640	1280	0,75	9	12	Equal	7	14	0	0	0	0	0,05
0,75575	320	320	640	0,75	9	12	Equal	7	15	0	0	0	0	0,05
0,84292	400	400	800	0,75	9	12	Equal	7	15	0	0	0	0	0,05
0,90153	480	480	960	0,75	9	12	Equal	7	15	0	0	0	0	0,05
0,93958	560	560	1120	0,75	9	12	Equal	7	15	0	0	0	0	0,05
0,96360	640	640	1280	0,75	9	12	Equal	7	15	0	0	0	0	0,05
0,77843	320	320	640	0,75	9	12	Equal	7	16	0	0	0	0	0,05
0,86201	400	400	800	0,75	9	12	Equal	7	16	0	0	0	0	0,05
0,91636	480	480	960	0,75	9	12	Equal	7	16	0	0	0	0	0,05
0,95044	560	560	1120	0,75	9	12	Equal	7	16	0	0	0	0	0,05
0,97120	640	640	1280	0,75	9	12	Equal	7	16	0	0	0	0	0,05
0,79797	320	320	640	0,75	9	12	Equal	7	17	0	0	0	0	0,05
0,87795	400	400	800	0,75	9	12	Equal	7	17	0	0	0	0	0,05
0,92833	480	480	960	0,75	9	12	Equal	7	17	0	0	0	0	0,05
0,95891	560	560	1120	0,75	9	12	Equal	7	17	0	0	0	0	0,05
0,97691	640	640	1280	0,75	9	12	Equal	7	17	0	0	0	0	0,05
0,81487	320	320	640	0,75	9	12	Equal	7	18	0	0	0	0	0,05
0,89131	400	400	800	0,75	9	12	Equal	7	18	0	0	0	0	0,05
0,93806	480	480	960	0,75	9	12	Equal	7	18	0	0	0	0	0,05
0,96557	560	560	1120	0,75	9	12	Equal	7	18	0	0	0	0	0,05
0,98126	640	640	1280	0,75	9	12	Equal	7	18	0	0	0	0	0,05

Logrank Tests

Numeric Results (Sample Size) (Continued)

Solve For: Power
Test Type: Two-Sample Logrank Test
Groups: 1 = Control, 2 = Treatment
Alternative Hypothesis: Two-Sided

	Sample Size			Hazard Ratio	Median Survival Time			Time		Proportion Lost*		Proportion Switching Groups*		
Power	N1	N2	N	HR	T1	T2	Accrual Pattern	Accrual	Total	Control	Treatment	Control to Treatment	Treatment to Control	Alpha
0,82953	320	320	640	0,75	9	12	Equal	7	19	0	0	0	0	0,05
0,90258	400	400	800	0,75	9	12	Equal	7	19	0	0	0	0	0,05
0,94602	480	480	960	0,75	9	12	Equal	7	19	0	0	0	0	0,05
0,97086	560	560	1120	0,75	9	12	Equal	7	19	0	0	0	0	0,05
0,98461	640	640	1280	0,75	9	12	Equal	7	19	0	0	0	0	0,05
0,84229	320	320	640	0,75	9	12	Equal	7	20	0	0	0	0	0,05
0,91213	400	400	800	0,75	9	12	Equal	7	20	0	0	0	0	0,05
0,95259	480	480	960	0,75	9	12	Equal	7	20	0	0	0	0	0,05
0,97509	560	560	1120	0,75	9	12	Equal	7	20	0	0	0	0	0,05
0,98721	640	640	1280	0,75	9	12	Equal	7	20	0	0	0	0	0,05
0,85344	320	320	640	0,75	9	12	Equal	7	21	0	0	0	0	0,05
0,92026	400	400	800	0,75	9	12	Equal	7	21	0	0	0	0	0,05
0,95803	480	480	960	0,75	9	12	Equal	7	21	0	0	0	0	0,05
0,97851	560	560	1120	0,75	9	12	Equal	7	21	0	0	0	0	0,05
0,98925	640	640	1280	0,75	9	12	Equal	7	21	0	0	0	0	0,05
0,86320	320	320	640	0,75	9	12	Equal	7	22	0	0	0	0	0,05
0,92723	400	400	800	0,75	9	12	Equal	7	22	0	0	0	0	0,05
0,96258	480	480	960	0,75	9	12	Equal	7	22	0	0	0	0	0,05
0,98129	560	560	1120	0,75	9	12	Equal	7	22	0	0	0	0	0,05
0,99087	640	640	1280	0,75	9	12	Equal	7	22	0	0	0	0	0,05
0,87179	320	320	640	0,75	9	12	Equal	7	23	0	0	0	0	0,05
0,93321	400	400	800	0,75	9	12	Equal	7	23	0	0	0	0	0,05
0,96640	480	480	960	0,75	9	12	Equal	7	23	0	0	0	0	0,05
0,98358	560	560	1120	0,75	9	12	Equal	7	23	0	0	0	0	0,05
0,99217	640	640	1280	0,75	9	12	Equal	7	23	0	0	0	0	0,05
0,87937	320	320	640	0,75	9	12	Equal	7	24	0	0	0	0	0,05
0,93838	400	400	800	0,75	9	12	Equal	7	24	0	0	0	0	0,05
0,96963	480	480	960	0,75	9	12	Equal	7	24	0	0	0	0	0,05

Logrank Tests

Numeric Results (Sample Size) (Continued)

Solve For: Power
Test Type: Two-Sample Logrank Test
Groups: 1 = Control, 2 = Treatment
Alternative Hypothesis: Two-Sided

	Sample Size			Hazard Ratio	Median Survival Time		Accrual	Time		Proportion Lost*		Proportion Switching Groups*		
Power	N1	N2	N	HR	T1	T2	Pattern	Accrual	Total	Control	Treatment	Control to Treatment	Treatment to Control	Alpha
0,98547	560	560	1120	0,75	9	12	Equal	7	24	0	0	0	0	0,05
0,99322	640	640	1280	0,75	9	12	Equal	7	24	0	0	0	0	0,05
0,88607	320	320	640	0,75	9	12	Equal	7	25	0	0	0	0	0,05
0,94287	400	400	800	0,75	9	12	Equal	7	25	0	0	0	0	0,05
0,97238	480	480	960	0,75	9	12	Equal	7	25	0	0	0	0	0,05
0,98704	560	560	1120	0,75	9	12	Equal	7	25	0	0	0	0	0,05
0,99407	640	640	1280	0,75	9	12	Equal	7	25	0	0	0	0	0,05
0,89202	320	320	640	0,75	9	12	Equal	7	26	0	0	0	0	0,05
0,94678	400	400	800	0,75	9	12	Equal	7	26	0	0	0	0	0,05
0,97473	480	480	960	0,75	9	12	Equal	7	26	0	0	0	0	0,05
0,98836	560	560	1120	0,75	9	12	Equal	7	26	0	0	0	0	0,05
0,99478	640	640	1280	0,75	9	12	Equal	7	26	0	0	0	0	0,05
0,59466	320	320	640	0,75	9	12	Equal	8	11	0	0	0	0	0,05
0,69161	400	400	800	0,75	9	12	Equal	8	11	0	0	0	0	0,05
0,76922	480	480	960	0,75	9	12	Equal	8	11	0	0	0	0	0,05
0,82975	560	560	1120	0,75	9	12	Equal	8	11	0	0	0	0	0,05
0,87597	640	640	1280	0,75	9	12	Equal	8	11	0	0	0	0	0,05
0,64054	320	320	640	0,75	9	12	Equal	8	12	0	0	0	0	0,05
0,73724	400	400	800	0,75	9	12	Equal	8	12	0	0	0	0	0,05
0,81151	480	480	960	0,75	9	12	Equal	8	12	0	0	0	0	0,05
0,86695	560	560	1120	0,75	9	12	Equal	8	12	0	0	0	0	0,05
0,90739	640	640	1280	0,75	9	12	Equal	8	12	0	0	0	0	0,05
0,67974	320	320	640	0,75	9	12	Equal	8	13	0	0	0	0	0,05
0,77472	400	400	800	0,75	9	12	Equal	8	13	0	0	0	0	0,05
0,84480	480	480	960	0,75	9	12	Equal	8	13	0	0	0	0	0,05
0,89497	560	560	1120	0,75	9	12	Equal	8	13	0	0	0	0	0,05
0,93002	640	640	1280	0,75	9	12	Equal	8	13	0	0	0	0	0,05
0,71325	320	320	640	0,75	9	12	Equal	8	14	0	0	0	0	0,05

Logrank Tests

Numeric Results (Sample Size) (Continued)

Solve For: Power
Test Type: Two-Sample Logrank Test
Groups: 1 = Control, 2 = Treatment
Alternative Hypothesis: Two-Sided

	Sample Size			Hazard Ratio	Median Survival Time			Time		Proportion Lost*		Proportion Switching Groups*		
Power	N1	N2	N	HR	T1	T2	Accrual Pattern	Accrual	Total	Control	Treatment	Control to Treatment	Treatment to Control	Alpha
0,80555	400	400	800	0,75	9	12	Equal	8	14	0	0	0	0	0,05
0,87111	480	480	960	0,75	9	12	Equal	8	14	0	0	0	0	0,05
0,91621	560	560	1120	0,75	9	12	Equal	8	14	0	0	0	0	0,05
0,94643	640	640	1280	0,75	9	12	Equal	8	14	0	0	0	0	0,05
0,74194	320	320	640	0,75	9	12	Equal	8	15	0	0	0	0	0,05
0,83100	400	400	800	0,75	9	12	Equal	8	15	0	0	0	0	0,05
0,89201	480	480	960	0,75	9	12	Equal	8	15	0	0	0	0	0,05
0,93242	560	560	1120	0,75	9	12	Equal	8	15	0	0	0	0	0,05
0,95845	640	640	1280	0,75	9	12	Equal	8	15	0	0	0	0	0,05
0,76655	320	320	640	0,75	9	12	Equal	8	16	0	0	0	0	0,05
0,85209	400	400	800	0,75	9	12	Equal	8	16	0	0	0	0	0,05
0,90871	480	480	960	0,75	9	12	Equal	8	16	0	0	0	0	0,05
0,94489	560	560	1120	0,75	9	12	Equal	8	16	0	0	0	0	0,05
0,96735	640	640	1280	0,75	9	12	Equal	8	16	0	0	0	0	0,05
0,78773	320	320	640	0,75	9	12	Equal	8	17	0	0	0	0	0,05
0,86966	400	400	800	0,75	9	12	Equal	8	17	0	0	0	0	0,05
0,92215	480	480	960	0,75	9	12	Equal	8	17	0	0	0	0	0,05
0,95457	560	560	1120	0,75	9	12	Equal	8	17	0	0	0	0	0,05
0,97401	640	640	1280	0,75	9	12	Equal	8	17	0	0	0	0	0,05
0,80601	320	320	640	0,75	9	12	Equal	8	18	0	0	0	0	0,05
0,88435	400	400	800	0,75	9	12	Equal	8	18	0	0	0	0	0,05
0,93303	480	480	960	0,75	9	12	Equal	8	18	0	0	0	0	0,05
0,96215	560	560	1120	0,75	9	12	Equal	8	18	0	0	0	0	0,05
0,97904	640	640	1280	0,75	9	12	Equal	8	18	0	0	0	0	0,05
0,82184	320	320	640	0,75	9	12	Equal	8	19	0	0	0	0	0,05
0,89670	400	400	800	0,75	9	12	Equal	8	19	0	0	0	0	0,05
0,94190	480	480	960	0,75	9	12	Equal	8	19	0	0	0	0	0,05
0,96813	560	560	1120	0,75	9	12	Equal	8	19	0	0	0	0	0,05

Logrank Tests

Numeric Results (Sample Size) (Continued)

Solve For: Power
Test Type: Two-Sample Logrank Test
Groups: 1 = Control, 2 = Treatment
Alternative Hypothesis: Two-Sided

	Sample Size			Hazard Ratio	Median Survival Time			Time		Proportion Lost*		Proportion Switching Groups*		
Power	N1	N2	N	HR	T1	T2	Accrual Pattern	Accrual	Total	Control	Treatment	Control to Treatment	Treatment to Control	Alpha
0,98290	640	640	1280	0,75	9	12	Equal	8	19	0	0	0	0	0,05
0,83559	320	320	640	0,75	9	12	Equal	8	20	0	0	0	0	0,05
0,90714	400	400	800	0,75	9	12	Equal	8	20	0	0	0	0	0,05
0,94918	480	480	960	0,75	9	12	Equal	8	20	0	0	0	0	0,05
0,97291	560	560	1120	0,75	9	12	Equal	8	20	0	0	0	0	0,05
0,98587	640	640	1280	0,75	9	12	Equal	8	20	0	0	0	0	0,05
0,84757	320	320	640	0,75	9	12	Equal	8	21	0	0	0	0	0,05
0,91601	400	400	800	0,75	9	12	Equal	8	21	0	0	0	0	0,05
0,95520	480	480	960	0,75	9	12	Equal	8	21	0	0	0	0	0,05
0,97674	560	560	1120	0,75	9	12	Equal	8	21	0	0	0	0	0,05
0,98820	640	640	1280	0,75	9	12	Equal	8	21	0	0	0	0	0,05
0,85806	320	320	640	0,75	9	12	Equal	8	22	0	0	0	0	0,05
0,92358	400	400	800	0,75	9	12	Equal	8	22	0	0	0	0	0,05
0,96021	480	480	960	0,75	9	12	Equal	8	22	0	0	0	0	0,05
0,97985	560	560	1120	0,75	9	12	Equal	8	22	0	0	0	0	0,05
0,99004	640	640	1280	0,75	9	12	Equal	8	22	0	0	0	0	0,05
0,86727	320	320	640	0,75	9	12	Equal	8	23	0	0	0	0	0,05
0,93007	400	400	800	0,75	9	12	Equal	8	23	0	0	0	0	0,05
0,96441	480	480	960	0,75	9	12	Equal	8	23	0	0	0	0	0,05
0,98239	560	560	1120	0,75	9	12	Equal	8	23	0	0	0	0	0,05
0,99150	640	640	1280	0,75	9	12	Equal	8	23	0	0	0	0	0,05
0,87537	320	320	640	0,75	9	12	Equal	8	24	0	0	0	0	0,05
0,93567	400	400	800	0,75	9	12	Equal	8	24	0	0	0	0	0,05
0,96794	480	480	960	0,75	9	12	Equal	8	24	0	0	0	0	0,05
0,98449	560	560	1120	0,75	9	12	Equal	8	24	0	0	0	0	0,05
0,99267	640	640	1280	0,75	9	12	Equal	8	24	0	0	0	0	0,05
0,88253	320	320	640	0,75	9	12	Equal	8	25	0	0	0	0	0,05
0,94051	400	400	800	0,75	9	12	Equal	8	25	0	0	0	0	0,05

Logrank Tests

Numeric Results (Sample Size) (Continued)

Solve For: Power
Test Type: Two-Sample Logrank Test
Groups: 1 = Control, 2 = Treatment
Alternative Hypothesis: Two-Sided

	Sample Size			Hazard Ratio	Median Survival Time			Time		Proportion Lost*		Proportion Switching Groups*		
Power	N1	N2	N	HR	T1	T2	Accrual Pattern	Accrual	Total	Control	Treatment	Control to Treatment	Treatment to Control	Alpha
0,97094	480	480	960	0,75	9	12	Equal	8	25	0	0	0	0	0,05
0,98622	560	560	1120	0,75	9	12	Equal	8	25	0	0	0	0	0,05
0,99363	640	640	1280	0,75	9	12	Equal	8	25	0	0	0	0	0,05
0,88888	320	320	640	0,75	9	12	Equal	8	26	0	0	0	0	0,05
0,94472	400	400	800	0,75	9	12	Equal	8	26	0	0	0	0	0,05
0,97350	480	480	960	0,75	9	12	Equal	8	26	0	0	0	0	0,05
0,98767	560	560	1120	0,75	9	12	Equal	8	26	0	0	0	0	0,05
0,99441	640	640	1280	0,75	9	12	Equal	8	26	0	0	0	0	0,05

* The reported proportions are during a single time period.

Power	The probability of rejecting a false null hypothesis when the alternative hypothesis is true.
N1, N2, and N	The sample sizes of the control group, treatment group, and both groups, respectively.
HR	Hazard Ratio. The controls group's median survival time divided by the treatment group's median survival time.
T1 and T2	The time until half the subjects fail.
Accrual Pattern	The pattern of accrual times across individual time periods.
Accrual Time	The number of time periods (years or months) during which accrual takes place.
Total Time	The total number of time periods in the study. Follow-up time = (Total Time) - (Accrual Time).
Control Lost	The proportion of the control group that is lost (drop out) during a single time period (year or month).
Treatment Lost	The proportion of the treatment group that is lost (drop out) during a single time period (year or month).
Control to Treatment	Drop In. The proportion of the control group that switch to a group with a hazard rate equal to the treatment group.
Treatment to Control	Noncompliance. The proportion of the treatment group that switch to a group with a hazard rate equal to the control group.
Alpha	The probability of rejecting a true null hypothesis.

Logrank Tests

Numeric Results (Events)

Solve For: Power
Test Type: Two-Sample Logrank Test
Groups: 1 = Control, 2 = Treatment
Alternative Hypothesis: Two-Sided

	Number of Events			Hazard Ratio	Median Survival Time			Time		Proportion Lost*		Proportion Switching Groups*		
Power	E1	E2	E	HR	T1	T2	Accrual Pattern	Accrual	Total	Control	Treatment	Control to Treatment	Treatment to Control	Alpha
0,68455	159,4	129,3	288,7	0,75	9	12	Equal	4	11	0	0	0	0	0,05
0,77923	199,2	161,6	360,8	0,75	9	12	Equal	4	11	0	0	0	0	0,05
0,84873	239,1	194,0	433,0	0,75	9	12	Equal	4	11	0	0	0	0	0,05
0,89820	278,9	226,3	505,2	0,75	9	12	Equal	4	11	0	0	0	0	0,05
0,93256	318,7	258,6	577,4	0,75	9	12	Equal	4	11	0	0	0	0	0,05
0,71738	171,3	140,0	311,3	0,75	9	12	Equal	4	12	0	0	0	0	0,05
0,80928	214,1	175,0	389,1	0,75	9	12	Equal	4	12	0	0	0	0	0,05
0,87423	256,9	210,0	466,9	0,75	9	12	Equal	4	12	0	0	0	0	0,05
0,91867	299,7	245,0	544,7	0,75	9	12	Equal	4	12	0	0	0	0	0,05
0,94829	342,6	280,0	622,6	0,75	9	12	Equal	4	12	0	0	0	0	0,05
0,74549	182,3	150,1	332,4	0,75	9	12	Equal	4	13	0	0	0	0	0,05
0,83409	227,9	187,6	415,5	0,75	9	12	Equal	4	13	0	0	0	0	0,05
0,89450	273,5	225,2	498,6	0,75	9	12	Equal	4	13	0	0	0	0	0,05
0,93431	319,0	262,7	581,7	0,75	9	12	Equal	4	13	0	0	0	0	0,05
0,95982	364,6	300,2	664,8	0,75	9	12	Equal	4	13	0	0	0	0	0,05
0,76961	192,5	159,6	352,2	0,75	9	12	Equal	4	14	0	0	0	0	0,05
0,85467	240,6	199,6	440,2	0,75	9	12	Equal	4	14	0	0	0	0	0,05
0,91072	288,8	239,5	528,2	0,75	9	12	Equal	4	14	0	0	0	0	0,05
0,94635	336,9	279,4	616,3	0,75	9	12	Equal	4	14	0	0	0	0	0,05
0,96838	385,0	319,3	704,3	0,75	9	12	Equal	4	14	0	0	0	0	0,05
0,79037	202,0	168,6	370,6	0,75	9	12	Equal	4	15	0	0	0	0	0,05
0,87181	252,4	210,8	463,3	0,75	9	12	Equal	4	15	0	0	0	0	0,05
0,92377	302,9	253,0	555,9	0,75	9	12	Equal	4	15	0	0	0	0	0,05
0,95571	353,4	295,1	648,6	0,75	9	12	Equal	4	15	0	0	0	0	0,05
0,97478	403,9	337,3	741,2	0,75	9	12	Equal	4	15	0	0	0	0	0,05
0,80830	210,7	177,1	387,8	0,75	9	12	Equal	4	16	0	0	0	0	0,05
0,88616	263,4	221,4	484,8	0,75	9	12	Equal	4	16	0	0	0	0	0,05
0,93435	316,1	265,7	581,8	0,75	9	12	Equal	4	16	0	0	0	0	0,05

Logrank Tests

Numeric Results (Events) (Continued)

Solve For: Power
Test Type: Two-Sample Logrank Test
Groups: 1 = Control, 2 = Treatment
Alternative Hypothesis: Two-Sided

	Number of Events			Hazard Ratio	Median Survival Time			Time		Proportion Lost*		Proportion Switching Groups*		
Power	E1	E2	E	HR	T1	T2	Accrual Pattern	Accrual	Total	Control	Treatment	Control to Treatment	Treatment to Control	Alpha
0,96305	368,7	310,0	678,7	0,75	9	12	Equal	4	16	0	0	0	0	0,05
0,97963	421,4	354,3	775,7	0,75	9	12	Equal	4	16	0	0	0	0	0,05
0,82382	218,8	185,2	404,0	0,75	9	12	Equal	4	17	0	0	0	0	0,05
0,89823	273,5	231,4	505,0	0,75	9	12	Equal	4	17	0	0	0	0	0,05
0,94298	328,2	277,7	606,0	0,75	9	12	Equal	4	17	0	0	0	0	0,05
0,96885	382,9	324,0	706,9	0,75	9	12	Equal	4	17	0	0	0	0	0,05
0,98335	437,6	370,3	807,9	0,75	9	12	Equal	4	17	0	0	0	0	0,05
0,83732	226,3	192,7	419,0	0,75	9	12	Equal	4	18	0	0	0	0	0,05
0,90844	282,9	240,9	523,8	0,75	9	12	Equal	4	18	0	0	0	0	0,05
0,95007	339,5	289,1	628,6	0,75	9	12	Equal	4	18	0	0	0	0	0,05
0,97348	396,0	337,3	733,3	0,75	9	12	Equal	4	18	0	0	0	0	0,05
0,98623	452,6	385,5	838,1	0,75	9	12	Equal	4	18	0	0	0	0	0,05
0,84909	233,3	199,9	433,1	0,75	9	12	Equal	4	19	0	0	0	0	0,05
0,91712	291,6	249,8	541,4	0,75	9	12	Equal	4	19	0	0	0	0	0,05
0,95594	349,9	299,8	649,7	0,75	9	12	Equal	4	19	0	0	0	0	0,05
0,97721	408,2	349,8	758,0	0,75	9	12	Equal	4	19	0	0	0	0	0,05
0,98848	466,5	399,7	866,3	0,75	9	12	Equal	4	19	0	0	0	0	0,05
0,85939	239,7	206,6	446,3	0,75	9	12	Equal	4	20	0	0	0	0	0,05
0,92453	299,6	258,3	557,9	0,75	9	12	Equal	4	20	0	0	0	0	0,05
0,96083	359,5	309,9	669,4	0,75	9	12	Equal	4	20	0	0	0	0	0,05
0,98023	419,4	361,6	781,0	0,75	9	12	Equal	4	20	0	0	0	0	0,05
0,99026	479,4	413,2	892,6	0,75	9	12	Equal	4	20	0	0	0	0	0,05
0,86844	245,6	213,0	458,6	0,75	9	12	Equal	4	21	0	0	0	0	0,05
0,93089	307,0	266,2	573,3	0,75	9	12	Equal	4	21	0	0	0	0	0,05
0,96493	368,5	319,5	687,9	0,75	9	12	Equal	4	21	0	0	0	0	0,05
0,98271	429,9	372,7	802,6	0,75	9	12	Equal	4	21	0	0	0	0	0,05
0,99168	491,3	426,0	917,2	0,75	9	12	Equal	4	21	0	0	0	0	0,05
0,87641	251,2	219,0	470,1	0,75	9	12	Equal	4	22	0	0	0	0	0,05

Logrank Tests

Numeric Results (Events) (Continued)

Solve For: Power
Test Type: Two-Sample Logrank Test
Groups: 1 = Control, 2 = Treatment
Alternative Hypothesis: Two-Sided

	Number of Events			Hazard Ratio	Median Survival Time		Accrual	Time		Proportion Lost*		Proportion Switching Groups*		
Power	E1	E2	E	HR	T1	T2	Pattern	Accrual	Total	Control	Treatment	Control to Treatment	Treatment to Control	Alpha
0,93638	313,9	273,7	587,7	0,75	9	12	Equal	4	22	0	0	0	0	0,05
0,96839	376,7	328,5	705,2	0,75	9	12	Equal	4	22	0	0	0	0	0,05
0,98474	439,5	383,2	822,7	0,75	9	12	Equal	4	22	0	0	0	0	0,05
0,99282	502,3	438,0	940,3	0,75	9	12	Equal	4	22	0	0	0	0	0,05
0,88345	256,3	224,7	480,9	0,75	9	12	Equal	4	23	0	0	0	0	0,05
0,94113	320,3	280,8	601,1	0,75	9	12	Equal	4	23	0	0	0	0	0,05
0,97132	384,4	337,0	721,4	0,75	9	12	Equal	4	23	0	0	0	0	0,05
0,98644	448,4	393,1	841,6	0,75	9	12	Equal	4	23	0	0	0	0	0,05
0,99375	512,5	449,3	961,8	0,75	9	12	Equal	4	23	0	0	0	0	0,05
0,88970	261,0	230,0	491,0	0,75	9	12	Equal	4	24	0	0	0	0	0,05
0,94526	326,2	287,5	613,7	0,75	9	12	Equal	4	24	0	0	0	0	0,05
0,97382	391,5	345,0	736,5	0,75	9	12	Equal	4	24	0	0	0	0	0,05
0,98785	456,7	402,5	859,2	0,75	9	12	Equal	4	24	0	0	0	0	0,05
0,99451	522,0	460,0	982,0	0,75	9	12	Equal	4	24	0	0	0	0	0,05
0,89525	265,4	235,1	500,4	0,75	9	12	Equal	4	25	0	0	0	0	0,05
0,94887	331,7	293,8	625,5	0,75	9	12	Equal	4	25	0	0	0	0	0,05
0,97597	398,0	352,6	750,6	0,75	9	12	Equal	4	25	0	0	0	0	0,05
0,98905	464,4	411,3	875,7	0,75	9	12	Equal	4	25	0	0	0	0	0,05
0,99514	530,7	470,1	1000,8	0,75	9	12	Equal	4	25	0	0	0	0	0,05
0,90019	269,4	239,8	509,2	0,75	9	12	Equal	4	26	0	0	0	0	0,05
0,95204	336,8	299,8	636,5	0,75	9	12	Equal	4	26	0	0	0	0	0,05
0,97782	404,1	359,7	763,8	0,75	9	12	Equal	4	26	0	0	0	0	0,05
0,99006	471,5	419,7	891,1	0,75	9	12	Equal	4	26	0	0	0	0	0,05
0,99566	538,8	479,6	1018,5	0,75	9	12	Equal	4	26	0	0	0	0	0,05
0,66514	152,7	123,5	276,2	0,75	9	12	Equal	5	11	0	0	0	0	0,05
0,76095	190,9	154,3	345,2	0,75	9	12	Equal	5	11	0	0	0	0	0,05
0,83274	229,0	185,2	414,2	0,75	9	12	Equal	5	11	0	0	0	0	0,05
0,88496	267,2	216,1	483,3	0,75	9	12	Equal	5	11	0	0	0	0	0,05

Logrank Tests

Numeric Results (Events) (Continued)

Solve For: Power
Test Type: Two-Sample Logrank Test
Groups: 1 = Control, 2 = Treatment
Alternative Hypothesis: Two-Sided

	Number of Events			Hazard Ratio	Median Survival Time			Time		Proportion Lost*		Proportion Switching Groups*		
Power	E1	E2	E	HR	T1	T2	Accrual Pattern	Accrual	Total	Control	Treatment	Control to Treatment	Treatment to Control	Alpha
0,92206	305,4	246,9	552,3	0,75	9	12	Equal	5	11	0	0	0	0	0,05
0,70078	165,1	134,5	299,6	0,75	9	12	Equal	5	12	0	0	0	0	0,05
0,79422	206,4	168,1	374,5	0,75	9	12	Equal	5	12	0	0	0	0	0,05
0,86157	247,6	201,8	449,4	0,75	9	12	Equal	5	12	0	0	0	0	0,05
0,90861	288,9	235,4	524,3	0,75	9	12	Equal	5	12	0	0	0	0	0,05
0,94064	330,2	269,0	599,2	0,75	9	12	Equal	5	12	0	0	0	0	0,05
0,73126	176,6	144,9	321,5	0,75	9	12	Equal	5	13	0	0	0	0	0,05
0,82164	220,7	181,1	401,9	0,75	9	12	Equal	5	13	0	0	0	0	0,05
0,88442	264,9	217,4	482,2	0,75	9	12	Equal	5	13	0	0	0	0	0,05
0,92661	309,0	253,6	562,6	0,75	9	12	Equal	5	13	0	0	0	0	0,05
0,95420	353,2	289,8	643,0	0,75	9	12	Equal	5	13	0	0	0	0	0,05
0,75739	187,2	154,7	341,9	0,75	9	12	Equal	5	14	0	0	0	0	0,05
0,84433	234,0	193,4	427,4	0,75	9	12	Equal	5	14	0	0	0	0	0,05
0,90264	280,8	232,1	512,9	0,75	9	12	Equal	5	14	0	0	0	0	0,05
0,94041	327,6	270,8	598,4	0,75	9	12	Equal	5	14	0	0	0	0	0,05
0,96419	374,4	309,5	683,9	0,75	9	12	Equal	5	14	0	0	0	0	0,05
0,77985	197,1	164,0	361,1	0,75	9	12	Equal	5	15	0	0	0	0	0,05
0,86319	246,3	205,0	451,3	0,75	9	12	Equal	5	15	0	0	0	0	0,05
0,91726	295,6	246,0	541,6	0,75	9	12	Equal	5	15	0	0	0	0	0,05
0,95108	344,8	287,0	631,9	0,75	9	12	Equal	5	15	0	0	0	0	0,05
0,97164	394,1	328,0	722,1	0,75	9	12	Equal	5	15	0	0	0	0	0,05
0,79920	206,2	172,8	378,9	0,75	9	12	Equal	5	16	0	0	0	0	0,05
0,87893	257,7	216,0	473,7	0,75	9	12	Equal	5	16	0	0	0	0	0,05
0,92906	309,2	259,2	568,4	0,75	9	12	Equal	5	16	0	0	0	0	0,05
0,95941	360,8	302,3	663,1	0,75	9	12	Equal	5	16	0	0	0	0	0,05
0,97725	412,3	345,5	757,9	0,75	9	12	Equal	5	16	0	0	0	0	0,05
0,81594	214,6	181,0	395,6	0,75	9	12	Equal	5	17	0	0	0	0	0,05
0,89214	268,3	226,3	494,5	0,75	9	12	Equal	5	17	0	0	0	0	0,05

Logrank Tests

Numeric Results (Events) (Continued)

Solve For: Power
Test Type: Two-Sample Logrank Test
Groups: 1 = Control, 2 = Treatment
Alternative Hypothesis: Two-Sided

	Number of Events			Hazard Ratio	Median Survival Time			Time		Proportion Lost*		Proportion Switching Groups*		
Power	E1	E2	E	HR	T1	T2	Accrual Pattern	Accrual	Total	Control	Treatment	Control to Treatment	Treatment to Control	Alpha
0,93866	321,9	271,6	593,5	0,75	9	12	Equal	5	17	0	0	0	0	0,05
0,96597	375,6	316,8	692,4	0,75	9	12	Equal	5	17	0	0	0	0	0,05
0,98152	429,2	362,1	791,3	0,75	9	12	Equal	5	17	0	0	0	0	0,05
0,83046	222,4	188,8	411,2	0,75	9	12	Equal	5	18	0	0	0	0	0,05
0,90328	278,0	236,0	514,1	0,75	9	12	Equal	5	18	0	0	0	0	0,05
0,94651	333,6	283,3	616,9	0,75	9	12	Equal	5	18	0	0	0	0	0,05
0,97118	389,2	330,5	719,7	0,75	9	12	Equal	5	18	0	0	0	0	0,05
0,98481	444,8	377,7	822,5	0,75	9	12	Equal	5	18	0	0	0	0	0,05
0,84310	229,6	196,2	425,8	0,75	9	12	Equal	5	19	0	0	0	0	0,05
0,91273	287,1	245,2	532,3	0,75	9	12	Equal	5	19	0	0	0	0	0,05
0,95299	344,5	294,3	638,8	0,75	9	12	Equal	5	19	0	0	0	0	0,05
0,97535	401,9	343,3	745,2	0,75	9	12	Equal	5	19	0	0	0	0	0,05
0,98736	459,3	392,4	851,7	0,75	9	12	Equal	5	19	0	0	0	0	0,05
0,85415	236,3	203,1	439,5	0,75	9	12	Equal	5	20	0	0	0	0	0,05
0,92078	295,4	253,9	549,4	0,75	9	12	Equal	5	20	0	0	0	0	0,05
0,95837	354,5	304,7	659,2	0,75	9	12	Equal	5	20	0	0	0	0	0,05
0,97872	413,6	355,5	769,1	0,75	9	12	Equal	5	20	0	0	0	0	0,05
0,98937	472,7	406,3	879,0	0,75	9	12	Equal	5	20	0	0	0	0	0,05
0,86383	242,5	209,7	452,2	0,75	9	12	Equal	5	21	0	0	0	0	0,05
0,92767	303,2	262,1	565,3	0,75	9	12	Equal	5	21	0	0	0	0	0,05
0,96286	363,8	314,6	678,4	0,75	9	12	Equal	5	21	0	0	0	0	0,05
0,98147	424,5	367,0	791,4	0,75	9	12	Equal	5	21	0	0	0	0	0,05
0,99097	485,1	419,4	904,5	0,75	9	12	Equal	5	21	0	0	0	0	0,05
0,87234	248,3	215,9	464,2	0,75	9	12	Equal	5	22	0	0	0	0	0,05
0,93359	310,4	269,9	580,2	0,75	9	12	Equal	5	22	0	0	0	0	0,05
0,96664	372,4	323,8	696,3	0,75	9	12	Equal	5	22	0	0	0	0	0,05
0,98372	434,5	377,8	812,3	0,75	9	12	Equal	5	22	0	0	0	0	0,05
0,99225	496,6	431,8	928,4	0,75	9	12	Equal	5	22	0	0	0	0	0,05

Logrank Tests

Numeric Results (Events) (Continued)

Solve For: Power
Test Type: Two-Sample Logrank Test
Groups: 1 = Control, 2 = Treatment
Alternative Hypothesis: Two-Sided

	Number of Events			Hazard Ratio	Median Survival Time			Time		Proportion Lost*		Proportion Switching Groups*		
Power	E1	E2	E	HR	T1	T2	Accrual Pattern	Accrual	Total	Control	Treatment	Control to Treatment	Treatment to Control	Alpha
0,87986	253,6	221,7	475,3	0,75	9	12	Equal	5	23	0	0	0	0	0,05
0,93871	317,0	277,2	594,2	0,75	9	12	Equal	5	23	0	0	0	0	0,05
0,96984	380,4	332,6	713,0	0,75	9	12	Equal	5	23	0	0	0	0	0,05
0,98559	443,8	388,0	831,8	0,75	9	12	Equal	5	23	0	0	0	0	0,05
0,99328	507,2	443,5	950,7	0,75	9	12	Equal	5	23	0	0	0	0	0,05
0,88651	258,5	227,3	485,8	0,75	9	12	Equal	5	24	0	0	0	0	0,05
0,94316	323,2	284,1	607,2	0,75	9	12	Equal	5	24	0	0	0	0	0,05
0,97255	387,8	340,9	728,7	0,75	9	12	Equal	5	24	0	0	0	0	0,05
0,98714	452,4	397,7	850,1	0,75	9	12	Equal	5	24	0	0	0	0	0,05
0,99413	517,1	454,5	971,6	0,75	9	12	Equal	5	24	0	0	0	0	0,05
0,89241	263,1	232,5	495,5	0,75	9	12	Equal	5	25	0	0	0	0	0,05
0,94703	328,9	290,6	619,4	0,75	9	12	Equal	5	25	0	0	0	0	0,05
0,97488	394,6	348,7	743,3	0,75	9	12	Equal	5	25	0	0	0	0	0,05
0,98844	460,4	406,8	867,2	0,75	9	12	Equal	5	25	0	0	0	0	0,05
0,99482	526,2	464,9	991,1	0,75	9	12	Equal	5	25	0	0	0	0	0,05
0,89766	267,3	237,4	504,7	0,75	9	12	Equal	5	26	0	0	0	0	0,05
0,95043	334,1	296,7	630,8	0,75	9	12	Equal	5	26	0	0	0	0	0,05
0,97688	401,0	356,1	757,0	0,75	9	12	Equal	5	26	0	0	0	0	0,05
0,98954	467,8	415,4	883,2	0,75	9	12	Equal	5	26	0	0	0	0	0,05
0,99539	534,6	474,7	1009,3	0,75	9	12	Equal	5	26	0	0	0	0	0,05
0,64383	145,7	117,4	263,1	0,75	9	12	Equal	6	11	0	0	0	0	0,05
0,74045	182,1	146,8	328,8	0,75	9	12	Equal	6	11	0	0	0	0	0,05
0,81442	218,5	176,1	394,6	0,75	9	12	Equal	6	11	0	0	0	0	0,05
0,86945	254,9	205,5	460,4	0,75	9	12	Equal	6	11	0	0	0	0	0,05
0,90946	291,3	234,8	526,1	0,75	9	12	Equal	6	11	0	0	0	0	0,05
0,68256	158,6	128,8	287,4	0,75	9	12	Equal	6	12	0	0	0	0	0,05
0,77736	198,2	161,0	359,2	0,75	9	12	Equal	6	12	0	0	0	0	0,05
0,84711	237,9	193,2	431,0	0,75	9	12	Equal	6	12	0	0	0	0	0,05

Logrank Tests

Numeric Results (Events) (Continued)

Solve For: Power
Test Type: Two-Sample Logrank Test
Groups: 1 = Control, 2 = Treatment
Alternative Hypothesis: Two-Sided

	Number of Events			Hazard Ratio	Median Survival Time		Accrual	Time		Proportion Lost*		Proportion Switching Groups*		
Power	E1	E2	E	HR	T1	T2	Pattern	Accrual	Total	Control	Treatment	Control to Treatment	Treatment to Control	Alpha
0,89687	277,5	225,4	502,9	0,75	9	12	Equal	6	12	0	0	0	0	0,05
0,93151	317,2	257,6	574,7	0,75	9	12	Equal	6	12	0	0	0	0	0,05
0,71567	170,5	139,5	310,0	0,75	9	12	Equal	6	13	0	0	0	0	0,05
0,80774	213,2	174,4	387,6	0,75	9	12	Equal	6	13	0	0	0	0	0,05
0,87294	255,8	209,3	465,1	0,75	9	12	Equal	6	13	0	0	0	0	0,05
0,91766	298,4	244,1	542,6	0,75	9	12	Equal	6	13	0	0	0	0	0,05
0,94752	341,1	279,0	620,1	0,75	9	12	Equal	6	13	0	0	0	0	0,05
0,74401	181,6	149,6	331,3	0,75	9	12	Equal	6	14	0	0	0	0	0,05
0,83281	227,0	187,0	414,1	0,75	9	12	Equal	6	14	0	0	0	0	0,05
0,89347	272,4	224,5	496,9	0,75	9	12	Equal	6	14	0	0	0	0	0,05
0,93353	317,8	261,9	579,7	0,75	9	12	Equal	6	14	0	0	0	0	0,05
0,95926	363,2	299,3	662,5	0,75	9	12	Equal	6	14	0	0	0	0	0,05
0,76834	191,9	159,2	351,1	0,75	9	12	Equal	6	15	0	0	0	0	0,05
0,85360	239,8	199,0	438,8	0,75	9	12	Equal	6	15	0	0	0	0	0,05
0,90989	287,8	238,8	526,6	0,75	9	12	Equal	6	15	0	0	0	0	0,05
0,94575	335,8	278,6	614,4	0,75	9	12	Equal	6	15	0	0	0	0	0,05
0,96795	383,8	318,4	702,2	0,75	9	12	Equal	6	15	0	0	0	0	0,05
0,78928	201,4	168,2	369,6	0,75	9	12	Equal	6	16	0	0	0	0	0,05
0,87092	251,7	210,3	462,0	0,75	9	12	Equal	6	16	0	0	0	0	0,05
0,92310	302,1	252,3	554,4	0,75	9	12	Equal	6	16	0	0	0	0	0,05
0,95524	352,4	294,4	646,8	0,75	9	12	Equal	6	16	0	0	0	0	0,05
0,97446	402,7	336,5	739,2	0,75	9	12	Equal	6	16	0	0	0	0	0,05
0,80735	210,2	176,7	386,9	0,75	9	12	Equal	6	17	0	0	0	0	0,05
0,88541	262,7	220,9	483,6	0,75	9	12	Equal	6	17	0	0	0	0	0,05
0,93380	315,3	265,1	580,4	0,75	9	12	Equal	6	17	0	0	0	0	0,05
0,96268	367,8	309,3	677,1	0,75	9	12	Equal	6	17	0	0	0	0	0,05
0,97939	420,3	353,5	773,8	0,75	9	12	Equal	6	17	0	0	0	0	0,05
0,82300	218,3	184,8	403,1	0,75	9	12	Equal	6	18	0	0	0	0	0,05

Logrank Tests

Numeric Results (Events) (Continued)

Solve For: Power
Test Type: Two-Sample Logrank Test
Groups: 1 = Control, 2 = Treatment
Alternative Hypothesis: Two-Sided

	Number of Events			Hazard Ratio	Median Survival Time		Accrual	Time		Proportion Lost*		Proportion Switching Groups*		
Power	E1	E2	E	HR	T1	T2	Pattern	Accrual	Total	Control	Treatment	Control to Treatment	Treatment to Control	Alpha
0,89760	272,9	231,0	503,9	0,75	9	12	Equal	6	18	0	0	0	0	0,05
0,94253	327,5	277,2	604,6	0,75	9	12	Equal	6	18	0	0	0	0	0,05
0,96856	382,0	323,4	705,4	0,75	9	12	Equal	6	18	0	0	0	0	0,05
0,98316	436,6	369,6	806,2	0,75	9	12	Equal	6	18	0	0	0	0	0,05
0,83660	225,8	192,4	418,2	0,75	9	12	Equal	6	19	0	0	0	0	0,05
0,90790	282,3	240,5	522,8	0,75	9	12	Equal	6	19	0	0	0	0	0,05
0,94970	338,8	288,6	627,3	0,75	9	12	Equal	6	19	0	0	0	0	0,05
0,97324	395,2	336,7	731,9	0,75	9	12	Equal	6	19	0	0	0	0	0,05
0,98608	451,7	384,7	836,4	0,75	9	12	Equal	6	19	0	0	0	0	0,05
0,84846	232,8	199,5	432,4	0,75	9	12	Equal	6	20	0	0	0	0	0,05
0,91666	291,0	249,4	540,5	0,75	9	12	Equal	6	20	0	0	0	0	0,05
0,95564	349,2	299,3	648,5	0,75	9	12	Equal	6	20	0	0	0	0	0,05
0,97702	407,4	349,2	756,6	0,75	9	12	Equal	6	20	0	0	0	0	0,05
0,98836	465,7	399,1	864,7	0,75	9	12	Equal	6	20	0	0	0	0	0,05
0,85884	239,3	206,3	445,6	0,75	9	12	Equal	6	21	0	0	0	0	0,05
0,92414	299,1	257,9	557,0	0,75	9	12	Equal	6	21	0	0	0	0	0,05
0,96058	358,9	309,4	668,4	0,75	9	12	Equal	6	21	0	0	0	0	0,05
0,98008	418,8	361,0	779,8	0,75	9	12	Equal	6	21	0	0	0	0	0,05
0,99017	478,6	412,6	891,2	0,75	9	12	Equal	6	21	0	0	0	0	0,05
0,86795	245,3	212,7	457,9	0,75	9	12	Equal	6	22	0	0	0	0	0,05
0,93055	306,6	265,8	572,4	0,75	9	12	Equal	6	22	0	0	0	0	0,05
0,96471	367,9	319,0	686,9	0,75	9	12	Equal	6	22	0	0	0	0	0,05
0,98258	429,2	372,2	801,4	0,75	9	12	Equal	6	22	0	0	0	0	0,05
0,99160	490,5	425,4	915,9	0,75	9	12	Equal	6	22	0	0	0	0	0,05
0,87598	250,8	218,7	469,5	0,75	9	12	Equal	6	23	0	0	0	0	0,05
0,93608	313,5	273,4	586,9	0,75	9	12	Equal	6	23	0	0	0	0	0,05
0,96820	376,2	328,1	704,3	0,75	9	12	Equal	6	23	0	0	0	0	0,05
0,98464	438,9	382,7	821,6	0,75	9	12	Equal	6	23	0	0	0	0	0,05

Logrank Tests

Numeric Results (Events) (Continued)

Solve For: Power
Test Type: Two-Sample Logrank Test
Groups: 1 = Control, 2 = Treatment
Alternative Hypothesis: Two-Sided

	Number of Events			Hazard Ratio	Median Survival Time			Time		Proportion Lost*		Proportion Switching Groups*		
Power	E1	E2	E	HR	T1	T2	Accrual Pattern	Accrual	Total	Control	Treatment	Control to Treatment	Treatment to Control	Alpha
0,99276	501,6	437,4	939,0	0,75	9	12	Equal	6	23	0	0	0	0	0,05
0,88307	255,9	224,4	480,3	0,75	9	12	Equal	6	24	0	0	0	0	0,05
0,94087	319,9	280,5	600,4	0,75	9	12	Equal	6	24	0	0	0	0	0,05
0,97116	383,9	336,6	720,5	0,75	9	12	Equal	6	24	0	0	0	0	0,05
0,98635	447,9	392,7	840,6	0,75	9	12	Equal	6	24	0	0	0	0	0,05
0,99370	511,9	448,8	960,7	0,75	9	12	Equal	6	24	0	0	0	0	0,05
0,88936	260,7	229,8	490,4	0,75	9	12	Equal	6	25	0	0	0	0	0,05
0,94504	325,9	287,2	613,1	0,75	9	12	Equal	6	25	0	0	0	0	0,05
0,97369	391,0	344,6	735,7	0,75	9	12	Equal	6	25	0	0	0	0	0,05
0,98778	456,2	402,1	858,3	0,75	9	12	Equal	6	25	0	0	0	0	0,05
0,99447	521,4	459,5	980,9	0,75	9	12	Equal	6	25	0	0	0	0	0,05
0,89494	265,1	234,8	499,9	0,75	9	12	Equal	6	26	0	0	0	0	0,05
0,94868	331,4	293,5	624,9	0,75	9	12	Equal	6	26	0	0	0	0	0,05
0,97585	397,6	352,2	749,9	0,75	9	12	Equal	6	26	0	0	0	0	0,05
0,98898	463,9	410,9	874,8	0,75	9	12	Equal	6	26	0	0	0	0	0,05
0,99510	530,2	469,6	999,8	0,75	9	12	Equal	6	26	0	0	0	0	0,05
0,62041	138,2	111,1	249,3	0,75	9	12	Equal	7	11	0	0	0	0	0,05
0,71745	172,8	138,9	311,6	0,75	9	12	Equal	7	11	0	0	0	0	0,05
0,79340	207,3	166,6	374,0	0,75	9	12	Equal	7	11	0	0	0	0	0,05
0,85123	241,9	194,4	436,3	0,75	9	12	Equal	7	11	0	0	0	0	0,05
0,89429	276,5	222,2	498,6	0,75	9	12	Equal	7	11	0	0	0	0	0,05
0,66254	151,7	122,8	274,5	0,75	9	12	Equal	7	12	0	0	0	0	0,05
0,75846	189,6	153,5	343,1	0,75	9	12	Equal	7	12	0	0	0	0	0,05
0,83054	227,6	184,2	411,8	0,75	9	12	Equal	7	12	0	0	0	0	0,05
0,88311	265,5	214,9	480,4	0,75	9	12	Equal	7	12	0	0	0	0	0,05
0,92057	303,4	245,6	549,0	0,75	9	12	Equal	7	12	0	0	0	0	0,05
0,69855	164,2	133,9	298,1	0,75	9	12	Equal	7	13	0	0	0	0	0,05
0,79217	205,2	167,4	372,6	0,75	9	12	Equal	7	13	0	0	0	0	0,05

Logrank Tests

Numeric Results (Events) (Continued)

Solve For: Power
Test Type: Two-Sample Logrank Test
Groups: 1 = Control, 2 = Treatment
Alternative Hypothesis: Two-Sided

	Number of Events			Hazard Ratio	Median Survival Time			Time		Proportion Lost*		Proportion Switching Groups*		
Power	E1	E2	E	HR	T1	T2	Accrual Pattern	Accrual	Total	Control	Treatment	Control to Treatment	Treatment to Control	Alpha
0,85983	246,3	200,8	447,1	0,75	9	12	Equal	7	13	0	0	0	0	0,05
0,90721	287,3	234,3	521,6	0,75	9	12	Equal	7	13	0	0	0	0	0,05
0,93956	328,4	267,8	596,1	0,75	9	12	Equal	7	13	0	0	0	0	0,05
0,72935	175,7	144,3	320,1	0,75	9	12	Equal	7	14	0	0	0	0	0,05
0,81995	219,7	180,4	400,1	0,75	9	12	Equal	7	14	0	0	0	0	0,05
0,88303	263,6	216,5	480,1	0,75	9	12	Equal	7	14	0	0	0	0	0,05
0,92553	307,5	252,6	560,1	0,75	9	12	Equal	7	14	0	0	0	0	0,05
0,95340	351,5	288,7	640,1	0,75	9	12	Equal	7	14	0	0	0	0	0,05
0,75575	186,4	154,2	340,6	0,75	9	12	Equal	7	15	0	0	0	0	0,05
0,84292	233,0	192,7	425,8	0,75	9	12	Equal	7	15	0	0	0	0	0,05
0,90153	279,6	231,3	510,9	0,75	9	12	Equal	7	15	0	0	0	0	0,05
0,93958	326,2	269,8	596,1	0,75	9	12	Equal	7	15	0	0	0	0	0,05
0,96360	372,8	308,4	681,2	0,75	9	12	Equal	7	15	0	0	0	0	0,05
0,77843	196,3	163,5	359,8	0,75	9	12	Equal	7	16	0	0	0	0	0,05
0,86201	245,4	204,4	449,8	0,75	9	12	Equal	7	16	0	0	0	0	0,05
0,91636	294,5	245,2	539,7	0,75	9	12	Equal	7	16	0	0	0	0	0,05
0,95044	343,6	286,1	629,7	0,75	9	12	Equal	7	16	0	0	0	0	0,05
0,97120	392,6	327,0	719,6	0,75	9	12	Equal	7	16	0	0	0	0	0,05
0,79797	205,5	172,3	377,8	0,75	9	12	Equal	7	17	0	0	0	0	0,05
0,87795	256,9	215,3	472,2	0,75	9	12	Equal	7	17	0	0	0	0	0,05
0,92833	308,2	258,4	566,7	0,75	9	12	Equal	7	17	0	0	0	0	0,05
0,95891	359,6	301,5	661,1	0,75	9	12	Equal	7	17	0	0	0	0	0,05
0,97691	411,0	344,6	755,5	0,75	9	12	Equal	7	17	0	0	0	0	0,05
0,81487	214,0	180,6	394,5	0,75	9	12	Equal	7	18	0	0	0	0	0,05
0,89131	267,5	225,7	493,2	0,75	9	12	Equal	7	18	0	0	0	0	0,05
0,93806	321,0	270,9	591,8	0,75	9	12	Equal	7	18	0	0	0	0	0,05
0,96557	374,5	316,0	690,5	0,75	9	12	Equal	7	18	0	0	0	0	0,05
0,98126	428,0	361,1	789,1	0,75	9	12	Equal	7	18	0	0	0	0	0,05

Logrank Tests

Numeric Results (Events) (Continued)

Solve For: Power
Test Type: Two-Sample Logrank Test
Groups: 1 = Control, 2 = Treatment
Alternative Hypothesis: Two-Sided

	Number of Events			Hazard Ratio	Median Survival Time			Time		Proportion Lost*		Proportion Switching Groups*		
Power	E1	E2	E	HR	T1	T2	Accrual Pattern	Accrual	Total	Control	Treatment	Control to Treatment	Treatment to Control	Alpha
0,82953	221,8	188,4	410,2	0,75	9	12	Equal	7	19	0	0	0	0	0,05
0,90258	277,3	235,5	512,8	0,75	9	12	Equal	7	19	0	0	0	0	0,05
0,94602	332,8	282,6	615,4	0,75	9	12	Equal	7	19	0	0	0	0	0,05
0,97086	388,2	329,7	717,9	0,75	9	12	Equal	7	19	0	0	0	0	0,05
0,98461	443,7	376,8	820,5	0,75	9	12	Equal	7	19	0	0	0	0	0,05
0,84229	229,1	195,8	424,9	0,75	9	12	Equal	7	20	0	0	0	0	0,05
0,91213	286,4	244,7	531,1	0,75	9	12	Equal	7	20	0	0	0	0	0,05
0,95259	343,7	293,7	637,3	0,75	9	12	Equal	7	20	0	0	0	0	0,05
0,97509	400,9	342,6	743,6	0,75	9	12	Equal	7	20	0	0	0	0	0,05
0,98721	458,2	391,6	849,8	0,75	9	12	Equal	7	20	0	0	0	0	0,05
0,85344	235,9	202,8	438,6	0,75	9	12	Equal	7	21	0	0	0	0	0,05
0,92026	294,8	253,4	548,3	0,75	9	12	Equal	7	21	0	0	0	0	0,05
0,95803	353,8	304,1	657,9	0,75	9	12	Equal	7	21	0	0	0	0	0,05
0,97851	412,7	354,8	767,6	0,75	9	12	Equal	7	21	0	0	0	0	0,05
0,98925	471,7	405,5	877,2	0,75	9	12	Equal	7	21	0	0	0	0	0,05
0,86320	242,1	209,3	451,4	0,75	9	12	Equal	7	22	0	0	0	0	0,05
0,92723	302,6	261,7	564,3	0,75	9	12	Equal	7	22	0	0	0	0	0,05
0,96258	363,1	314,0	677,1	0,75	9	12	Equal	7	22	0	0	0	0	0,05
0,98129	423,7	366,3	790,0	0,75	9	12	Equal	7	22	0	0	0	0	0,05
0,99087	484,2	418,7	902,8	0,75	9	12	Equal	7	22	0	0	0	0	0,05
0,87179	247,9	215,5	463,4	0,75	9	12	Equal	7	23	0	0	0	0	0,05
0,93321	309,8	269,4	579,3	0,75	9	12	Equal	7	23	0	0	0	0	0,05
0,96640	371,8	323,3	695,1	0,75	9	12	Equal	7	23	0	0	0	0	0,05
0,98358	433,8	377,2	811,0	0,75	9	12	Equal	7	23	0	0	0	0	0,05
0,99217	495,7	431,1	926,8	0,75	9	12	Equal	7	23	0	0	0	0	0,05
0,87937	253,2	221,4	474,6	0,75	9	12	Equal	7	24	0	0	0	0	0,05
0,93838	316,5	276,8	593,3	0,75	9	12	Equal	7	24	0	0	0	0	0,05
0,96963	379,8	332,1	711,9	0,75	9	12	Equal	7	24	0	0	0	0	0,05

Logrank Tests

Numeric Results (Events) (Continued)

Solve For: Power
Test Type: Two-Sample Logrank Test
Groups: 1 = Control, 2 = Treatment
Alternative Hypothesis: Two-Sided

	Number of Events			Hazard Ratio	Median Survival Time			Time		Proportion Lost*		Proportion Switching Groups*		
Power	E1	E2	E	HR	T1	T2	Accrual Pattern	Accrual	Total	Control	Treatment	Control to Treatment	Treatment to Control	Alpha
0,98547	443,1	387,5	830,6	0,75	9	12	Equal	7	24	0	0	0	0	0,05
0,99322	506,4	442,8	949,2	0,75	9	12	Equal	7	24	0	0	0	0	0,05
0,88607	258,2	226,9	485,1	0,75	9	12	Equal	7	25	0	0	0	0	0,05
0,94287	322,7	283,7	606,4	0,75	9	12	Equal	7	25	0	0	0	0	0,05
0,97238	387,2	340,4	727,7	0,75	9	12	Equal	7	25	0	0	0	0	0,05
0,98704	451,8	397,1	848,9	0,75	9	12	Equal	7	25	0	0	0	0	0,05
0,99407	516,3	453,9	970,2	0,75	9	12	Equal	7	25	0	0	0	0	0,05
0,89202	262,7	232,2	494,9	0,75	9	12	Equal	7	26	0	0	0	0	0,05
0,94678	328,4	290,2	618,6	0,75	9	12	Equal	7	26	0	0	0	0	0,05
0,97473	394,1	348,2	742,4	0,75	9	12	Equal	7	26	0	0	0	0	0,05
0,98836	459,8	406,3	866,1	0,75	9	12	Equal	7	26	0	0	0	0	0,05
0,99478	525,5	464,3	989,8	0,75	9	12	Equal	7	26	0	0	0	0	0,05
0,59466	130,4	104,5	234,9	0,75	9	12	Equal	8	11	0	0	0	0	0,05
0,69161	163,0	130,7	293,6	0,75	9	12	Equal	8	11	0	0	0	0	0,05
0,76922	195,6	156,8	352,4	0,75	9	12	Equal	8	11	0	0	0	0	0,05
0,82975	228,2	182,9	411,1	0,75	9	12	Equal	8	11	0	0	0	0	0,05
0,87597	260,8	209,0	469,8	0,75	9	12	Equal	8	11	0	0	0	0	0,05
0,64054	144,4	116,6	261,1	0,75	9	12	Equal	8	12	0	0	0	0	0,05
0,73724	180,6	145,8	326,3	0,75	9	12	Equal	8	12	0	0	0	0	0,05
0,81151	216,7	174,9	391,6	0,75	9	12	Equal	8	12	0	0	0	0	0,05
0,86695	252,8	204,1	456,9	0,75	9	12	Equal	8	12	0	0	0	0	0,05
0,90739	288,9	233,2	522,1	0,75	9	12	Equal	8	12	0	0	0	0	0,05
0,67974	157,5	128,0	285,5	0,75	9	12	Equal	8	13	0	0	0	0	0,05
0,77472	196,8	160,0	356,9	0,75	9	12	Equal	8	13	0	0	0	0	0,05
0,84480	236,2	192,0	428,2	0,75	9	12	Equal	8	13	0	0	0	0	0,05
0,89497	275,6	224,1	499,6	0,75	9	12	Equal	8	13	0	0	0	0	0,05
0,93002	314,9	256,1	571,0	0,75	9	12	Equal	8	13	0	0	0	0	0,05
0,71325	169,5	138,8	308,3	0,75	9	12	Equal	8	14	0	0	0	0	0,05

Logrank Tests

Numeric Results (Events) (Continued)

Solve For: Power
Test Type: Two-Sample Logrank Test
Groups: 1 = Control, 2 = Treatment
Alternative Hypothesis: Two-Sided

	Number of Events			Hazard Ratio	Median Survival Time		Accrual	Time		Proportion Lost*		Proportion Switching Groups*		
Power	E1	E2	E	HR	T1	T2	Pattern	Accrual	Total	Control	Treatment	Control to Treatment	Treatment to Control	Alpha
0,80555	211,9	173,5	385,4	0,75	9	12	Equal	8	14	0	0	0	0	0,05
0,87111	254,3	208,2	462,5	0,75	9	12	Equal	8	14	0	0	0	0	0,05
0,91621	296,6	242,9	539,6	0,75	9	12	Equal	8	14	0	0	0	0	0,05
0,94643	339,0	277,6	616,6	0,75	9	12	Equal	8	14	0	0	0	0	0,05
0,74194	180,7	149,0	329,6	0,75	9	12	Equal	8	15	0	0	0	0	0,05
0,83100	225,8	186,2	412,0	0,75	9	12	Equal	8	15	0	0	0	0	0,05
0,89201	271,0	223,5	494,5	0,75	9	12	Equal	8	15	0	0	0	0	0,05
0,93242	316,2	260,7	576,9	0,75	9	12	Equal	8	15	0	0	0	0	0,05
0,95845	361,3	298,0	659,3	0,75	9	12	Equal	8	15	0	0	0	0	0,05
0,76655	191,0	158,6	349,6	0,75	9	12	Equal	8	16	0	0	0	0	0,05
0,85209	238,7	198,2	437,0	0,75	9	12	Equal	8	16	0	0	0	0	0,05
0,90871	286,5	237,9	524,4	0,75	9	12	Equal	8	16	0	0	0	0	0,05
0,94489	334,2	277,5	611,7	0,75	9	12	Equal	8	16	0	0	0	0	0,05
0,96735	382,0	317,1	699,1	0,75	9	12	Equal	8	16	0	0	0	0	0,05
0,78773	200,6	167,6	368,2	0,75	9	12	Equal	8	17	0	0	0	0	0,05
0,86966	250,7	209,5	460,2	0,75	9	12	Equal	8	17	0	0	0	0	0,05
0,92215	300,8	251,5	552,3	0,75	9	12	Equal	8	17	0	0	0	0	0,05
0,95457	351,0	293,4	644,3	0,75	9	12	Equal	8	17	0	0	0	0	0,05
0,97401	401,1	335,3	736,4	0,75	9	12	Equal	8	17	0	0	0	0	0,05
0,80601	209,4	176,2	385,6	0,75	9	12	Equal	8	18	0	0	0	0	0,05
0,88435	261,8	220,2	482,0	0,75	9	12	Equal	8	18	0	0	0	0	0,05
0,93303	314,1	264,3	578,4	0,75	9	12	Equal	8	18	0	0	0	0	0,05
0,96215	366,5	308,3	674,8	0,75	9	12	Equal	8	18	0	0	0	0	0,05
0,97904	418,8	352,4	771,2	0,75	9	12	Equal	8	18	0	0	0	0	0,05
0,82184	217,6	184,3	401,9	0,75	9	12	Equal	8	19	0	0	0	0	0,05
0,89670	272,0	230,3	502,3	0,75	9	12	Equal	8	19	0	0	0	0	0,05
0,94190	326,4	276,4	602,8	0,75	9	12	Equal	8	19	0	0	0	0	0,05
0,96813	380,8	322,5	703,3	0,75	9	12	Equal	8	19	0	0	0	0	0,05

Logrank Tests

Numeric Results (Events) (Continued)

Solve For: Power
Test Type: Two-Sample Logrank Test
Groups: 1 = Control, 2 = Treatment
Alternative Hypothesis: Two-Sided

	Number of Events			Hazard Ratio	Median Survival Time		Accrual	Time		Proportion Lost*		Proportion Switching Groups*		
Power	E1	E2	E	HR	T1	T2	Pattern	Accrual	Total	Control	Treatment	Control to Treatment	Treatment to Control	Alpha
0,98290	435,2	368,5	803,7	0,75	9	12	Equal	8	19	0	0	0	0	0,05
0,83559	225,2	191,9	417,1	0,75	9	12	Equal	8	20	0	0	0	0	0,05
0,90714	281,5	239,8	521,3	0,75	9	12	Equal	8	20	0	0	0	0	0,05
0,94918	337,8	287,8	625,6	0,75	9	12	Equal	8	20	0	0	0	0	0,05
0,97291	394,1	335,8	729,9	0,75	9	12	Equal	8	20	0	0	0	0	0,05
0,98587	450,4	383,8	834,1	0,75	9	12	Equal	8	20	0	0	0	0	0,05
0,84757	232,2	199,1	431,3	0,75	9	12	Equal	8	21	0	0	0	0	0,05
0,91601	290,3	248,8	539,1	0,75	9	12	Equal	8	21	0	0	0	0	0,05
0,95520	348,3	298,6	646,9	0,75	9	12	Equal	8	21	0	0	0	0	0,05
0,97674	406,4	348,4	754,8	0,75	9	12	Equal	8	21	0	0	0	0	0,05
0,98820	464,4	398,1	862,6	0,75	9	12	Equal	8	21	0	0	0	0	0,05
0,85806	238,7	205,9	444,6	0,75	9	12	Equal	8	22	0	0	0	0	0,05
0,92358	298,4	257,3	555,7	0,75	9	12	Equal	8	22	0	0	0	0	0,05
0,96021	358,1	308,8	666,9	0,75	9	12	Equal	8	22	0	0	0	0	0,05
0,97985	417,8	360,2	778,0	0,75	9	12	Equal	8	22	0	0	0	0	0,05
0,99004	477,5	411,7	889,2	0,75	9	12	Equal	8	22	0	0	0	0	0,05
0,86727	244,8	212,3	457,0	0,75	9	12	Equal	8	23	0	0	0	0	0,05
0,93007	305,9	265,3	571,3	0,75	9	12	Equal	8	23	0	0	0	0	0,05
0,96441	367,1	318,4	685,5	0,75	9	12	Equal	8	23	0	0	0	0	0,05
0,98239	428,3	371,5	799,8	0,75	9	12	Equal	8	23	0	0	0	0	0,05
0,99150	489,5	424,5	914,0	0,75	9	12	Equal	8	23	0	0	0	0	0,05
0,87537	250,3	218,3	468,6	0,75	9	12	Equal	8	24	0	0	0	0	0,05
0,93567	312,9	272,9	585,8	0,75	9	12	Equal	8	24	0	0	0	0	0,05
0,96794	375,5	327,5	703,0	0,75	9	12	Equal	8	24	0	0	0	0	0,05
0,98449	438,1	382,0	820,1	0,75	9	12	Equal	8	24	0	0	0	0	0,05
0,99267	500,7	436,6	937,3	0,75	9	12	Equal	8	24	0	0	0	0	0,05
0,88253	255,5	224,0	479,5	0,75	9	12	Equal	8	25	0	0	0	0	0,05
0,94051	319,4	280,0	599,4	0,75	9	12	Equal	8	25	0	0	0	0	0,05

Logrank Tests

Numeric Results (Events) (Continued)

Solve For: Power
Test Type: Two-Sample Logrank Test
Groups: 1 = Control, 2 = Treatment
Alternative Hypothesis: Two-Sided

	Number of Events			Hazard Ratio	Median Survival Time			Time		Proportion Lost*		Proportion Switching Groups*		
Power	E1	E2	E	HR	T1	T2	Accrual Pattern	Accrual	Total	Control	Treatment	Control to Treatment	Treatment to Control	Alpha
0,97094	383,2	336,0	719,3	0,75	9	12	Equal	8	25	0	0	0	0	0,05
0,98622	447,1	392,0	839,1	0,75	9	12	Equal	8	25	0	0	0	0	0,05
0,99363	511,0	448,0	959,0	0,75	9	12	Equal	8	25	0	0	0	0	0,05
0,88888	260,3	229,4	489,7	0,75	9	12	Equal	8	26	0	0	0	0	0,05
0,94472	325,3	286,8	612,1	0,75	9	12	Equal	8	26	0	0	0	0	0,05
0,97350	390,4	344,1	734,5	0,75	9	12	Equal	8	26	0	0	0	0	0,05
0,98767	455,5	401,5	856,9	0,75	9	12	Equal	8	26	0	0	0	0	0,05
0,99441	520,6	458,8	979,4	0,75	9	12	Equal	8	26	0	0	0	0	0,05

* The reported proportions are during a single time period.

Power	The probability of rejecting a false null hypothesis when the alternative hypothesis is true.
E1, E2, and E	The number of events in the control group, treatment group, and both groups, respectively.
HR	Hazard Ratio. The controls group's median survival time divided by the treatment group's median survival time.
T1 and T2	The time until half the subjects fail.
Accrual Pattern	The pattern of accrual times across individual time periods.
Accrual Time	The number of time periods (years or months) during which accrual takes place.
Total Time	The total number of time periods in the study. Follow-up time = (Total Time) - (Accrual Time).
Control Lost	The proportion of the control group that is lost (drop out) during a single time period (year or month).
Treatment Lost	The proportion of the treatment group that is lost (drop out) during a single time period (year or month).
Control to Treatment	Drop In. The proportion of the control group that switch to a group with a hazard rate equal to the treatment group.
Treatment to Control	Noncompliance. The proportion of the treatment group that switch to a group with a hazard rate equal to the control group.
Alpha	The probability of rejecting a true null hypothesis.

Logrank Tests

Summary Statements

A parallel, two-group design will be used to test whether the Group 2 (treatment) hazard rate is different from the Group 1 (control) hazard rate. The comparison will be made using a two-sided, two-sample logrank test with a Type I error rate (α) of 0,05. The total duration of the study will be 11 time periods, with subject accrual (entry) occurring in the first 4 time periods. The accrual pattern across time periods will be uniform (all periods equal). It is anticipated that no subjects will drop out of the control group. It is anticipated that no subjects will drop out of the treatment group. The proportion of subjects switching from the control group to another group with a median survival time equal to that of the treatment group is 0 (per time period). The proportion of subjects switching from the treatment group to another group with a median survival time equal to that of the control group is 0 (per time period). To detect a hazard ratio (h2 / h1) of 0,75 when the control group median survival time is 9, with a sample size of 320 subjects in Group 1 and 320 subjects in Group 2 (totaling 640 subjects), the power is 0,68455. The corresponding number of events is 159,4 in Group 1 and 129,3 in Group 2 (totaling 288,7 events). The power was computed using PASS 2024, version 24.0.9.

References

Lakatos, Edward. 1988. 'Sample Sizes Based on the Log-Rank Statistic in Complex Clinical Trials', Biometrics, Volume 44, March, pages 229-241.
Lakatos, Edward. 2002. 'Designing Complex Group Sequential Survival Trials', Statistics in Medicine, Volume 21, pages 1969-1989.

Citation

PASS 2024 Power Analysis and Sample Size Software (2024). NCSS, LLC. Kaysville, Utah, USA, ncss.com/software/pass.

Logrank Tests

Plots

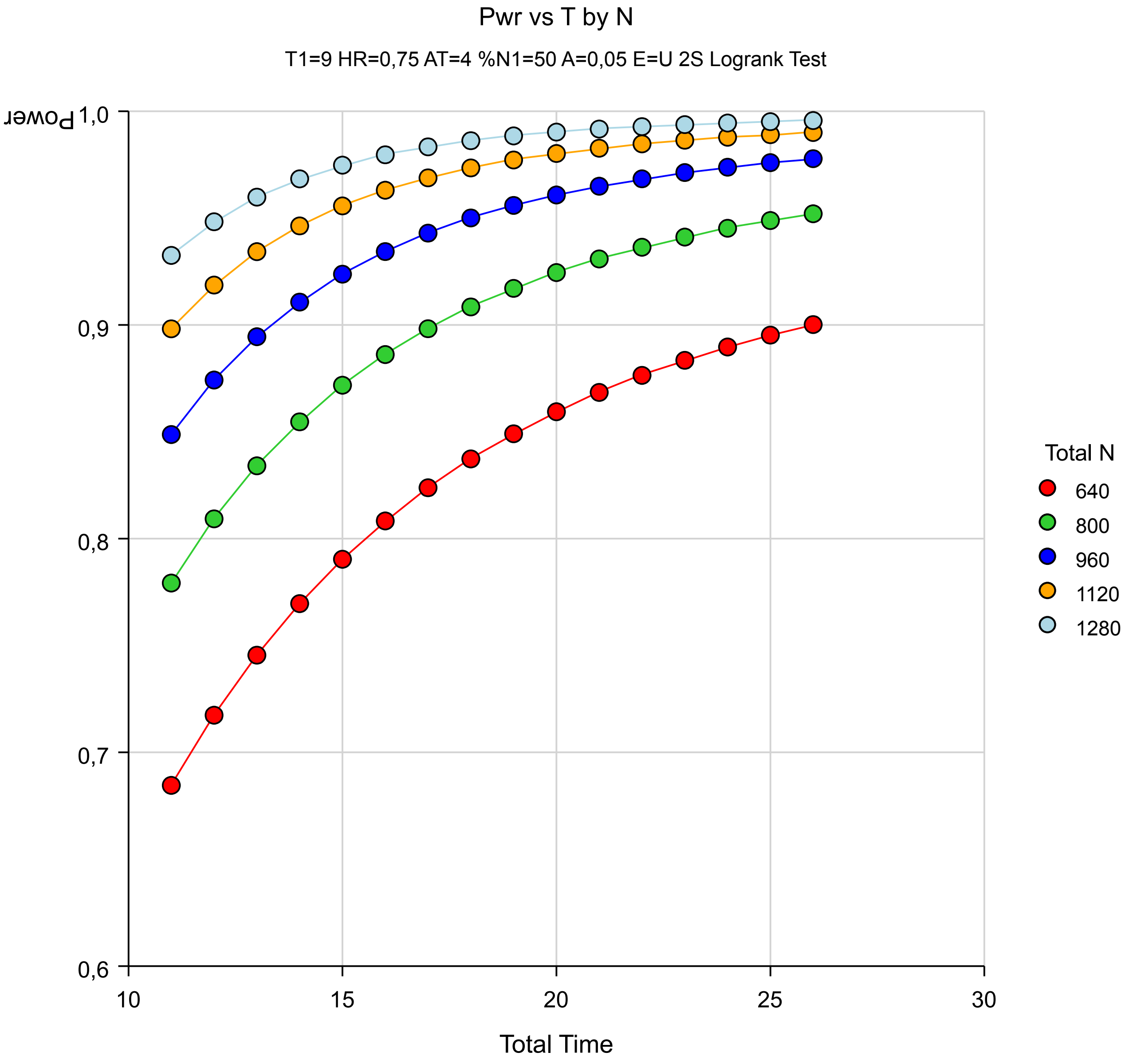

Logrank Tests

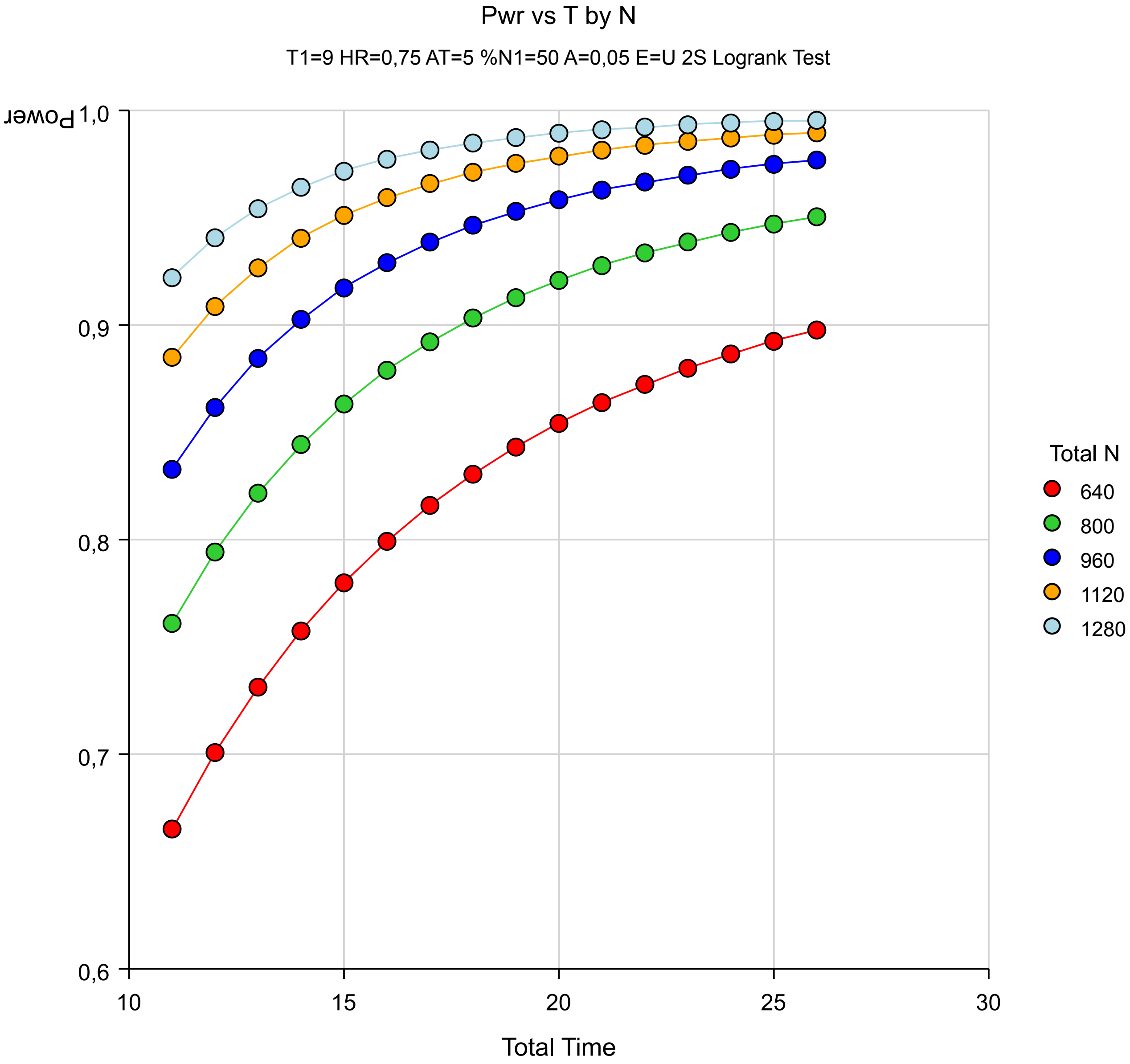

Logrank Tests

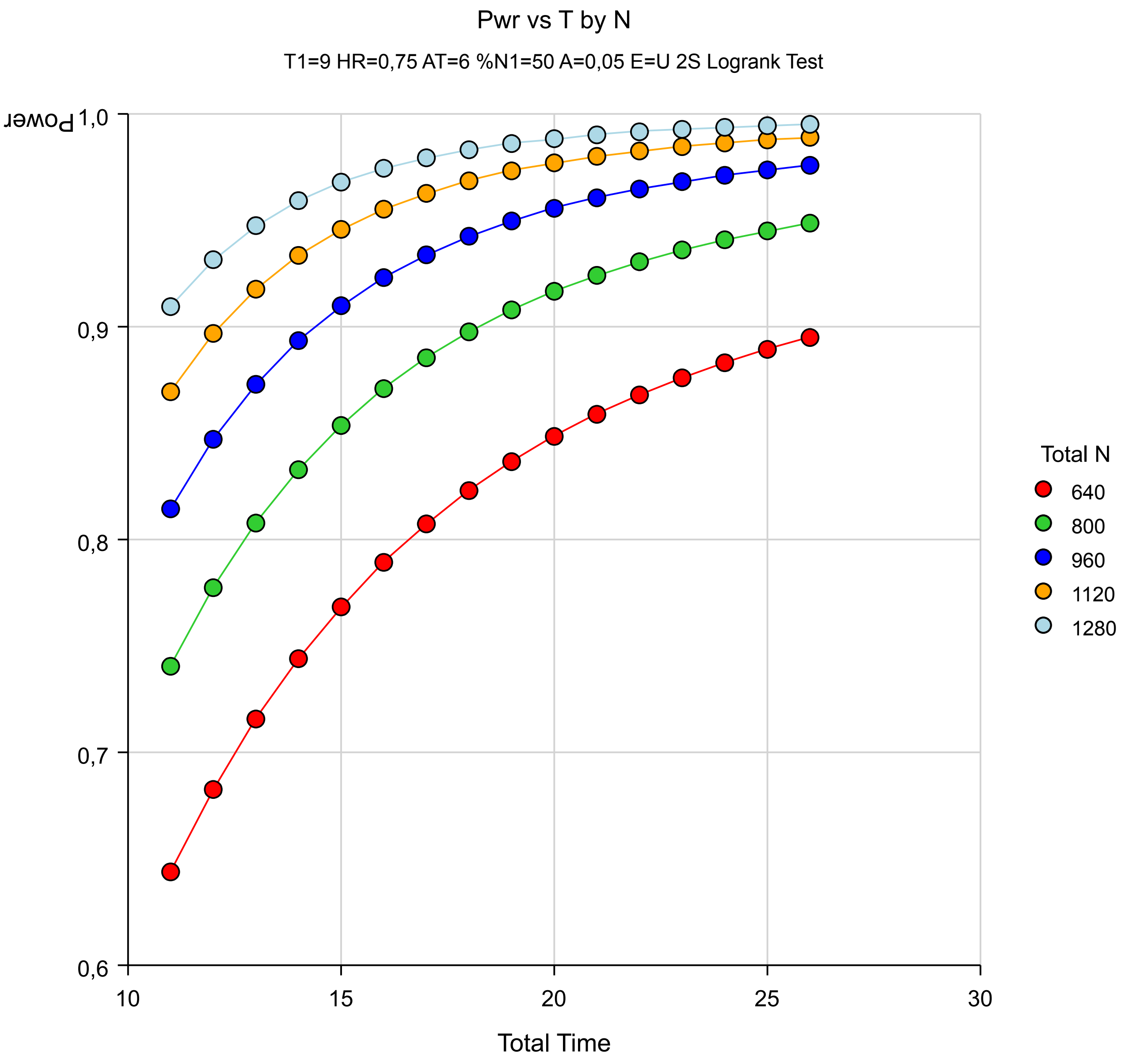

Logrank Tests

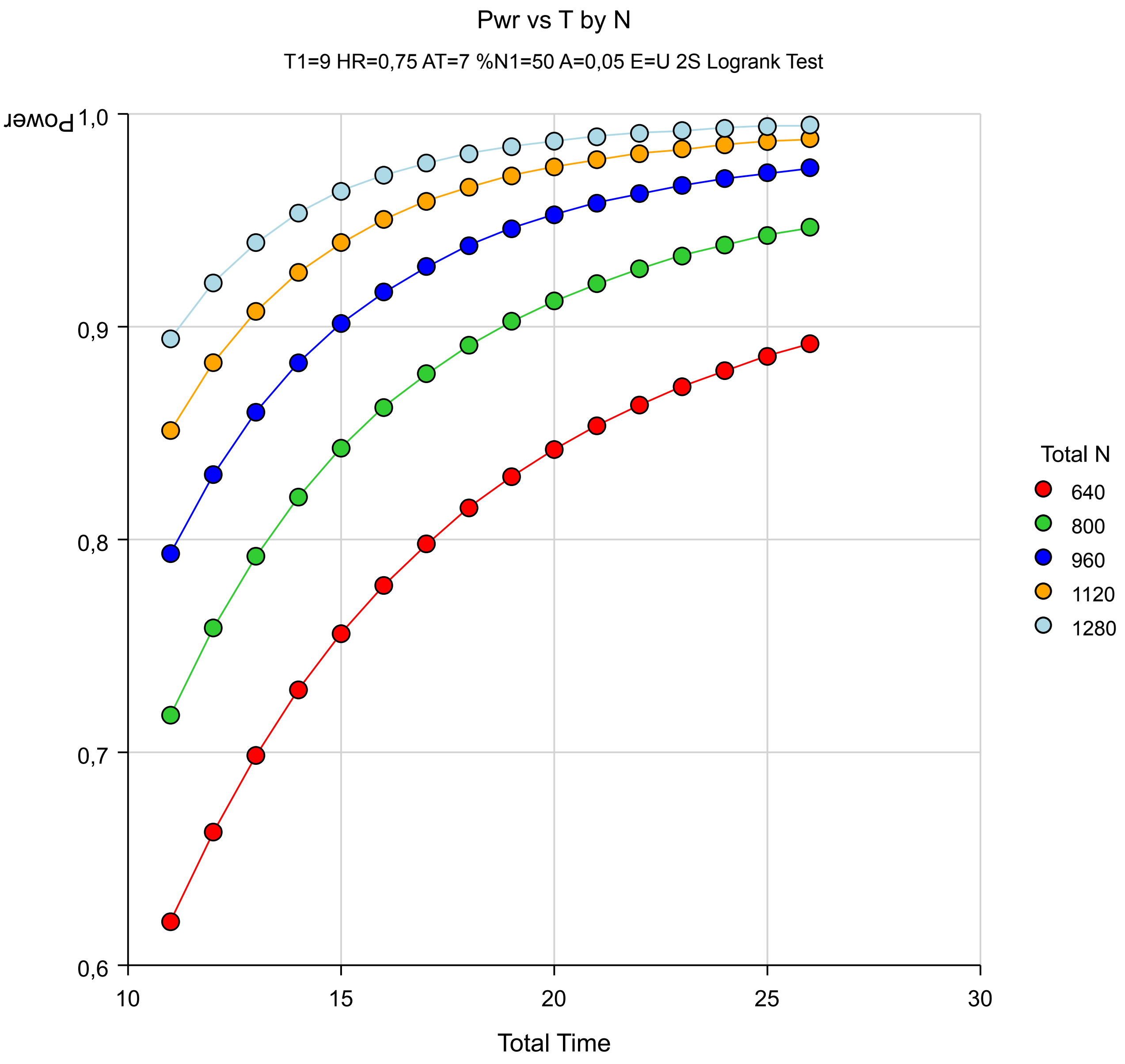

Logrank Tests

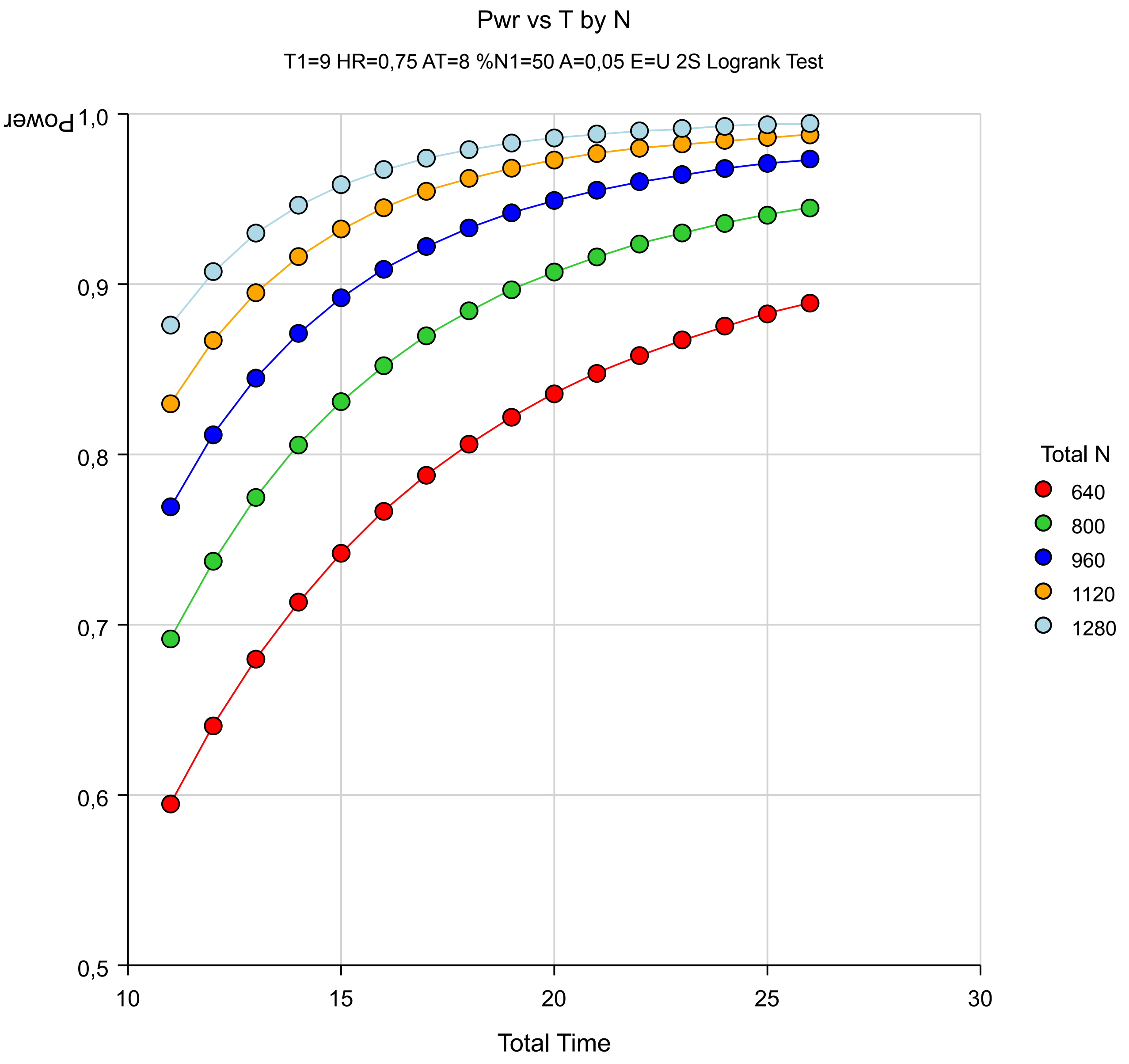

Logrank Tests

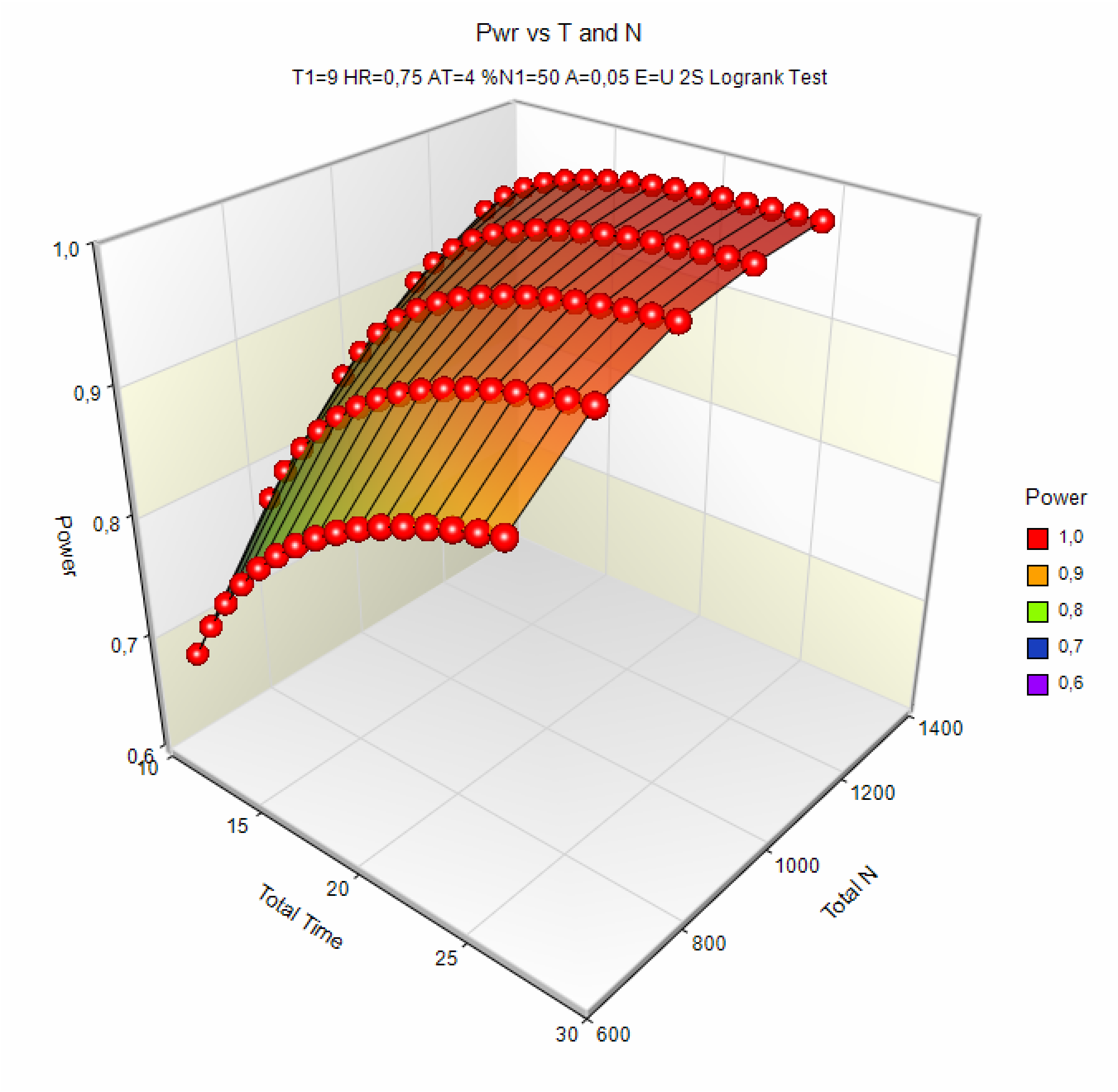

Logrank Tests

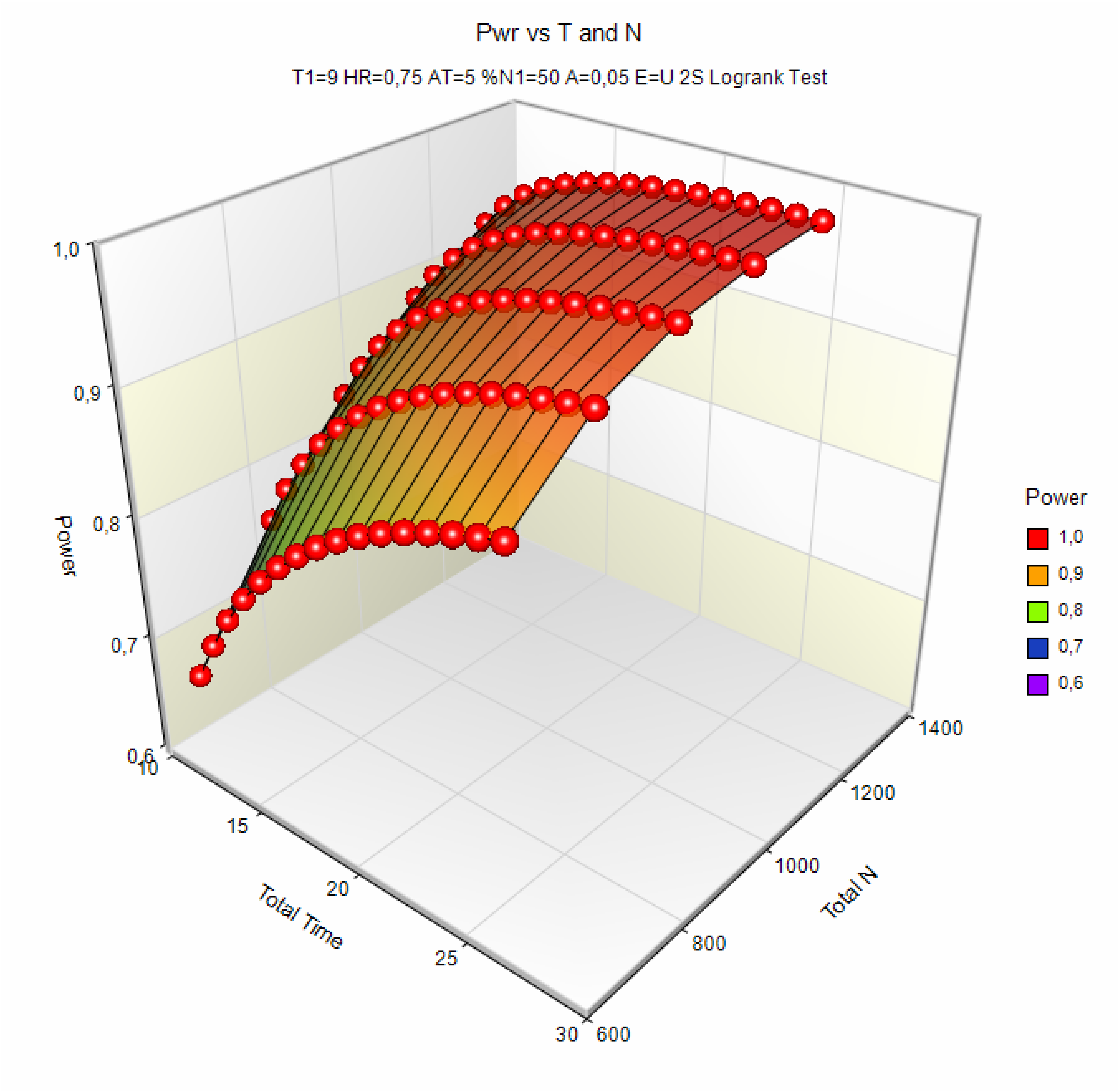

Logrank Tests

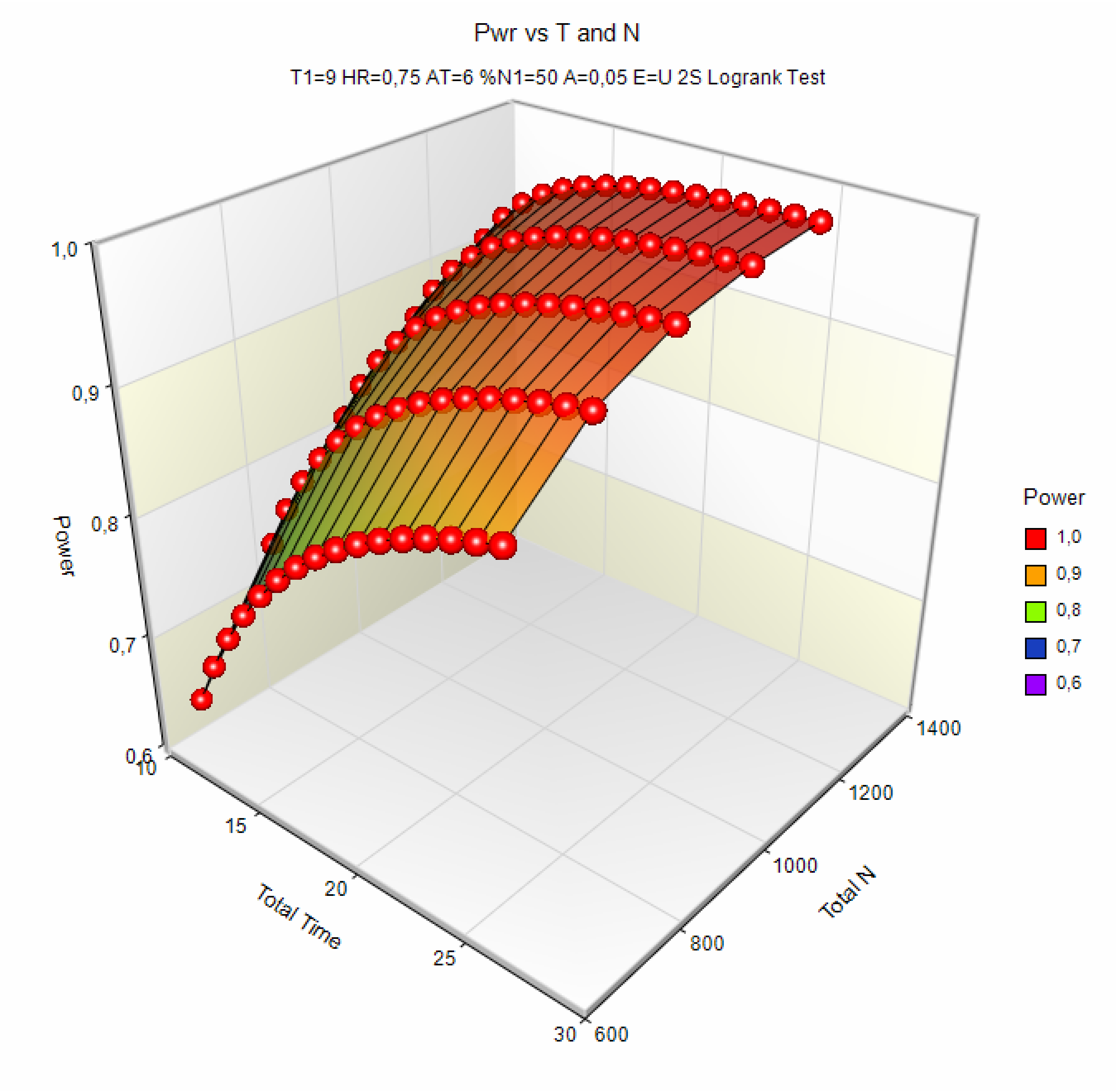

Logrank Tests

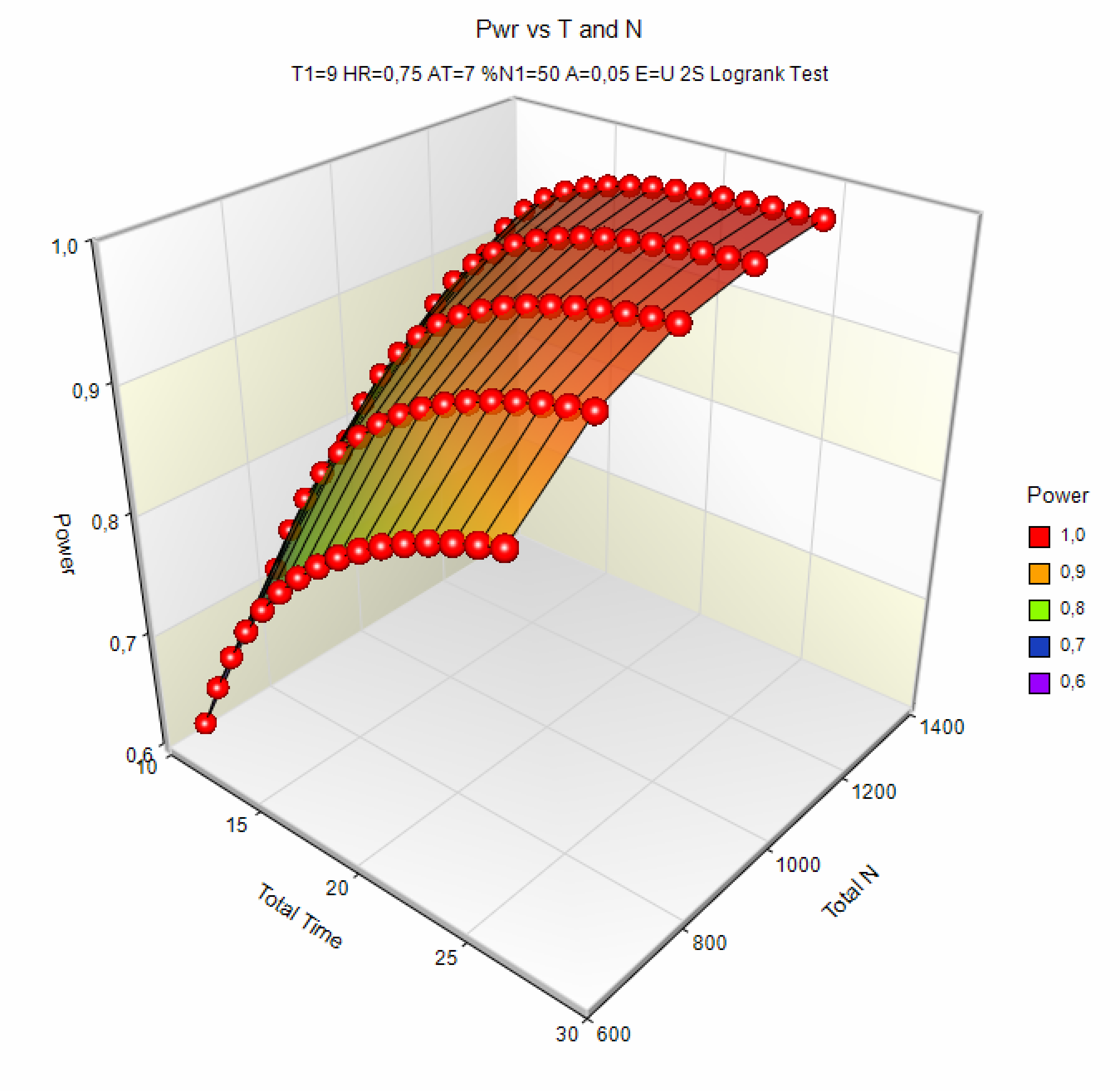

Logrank Tests

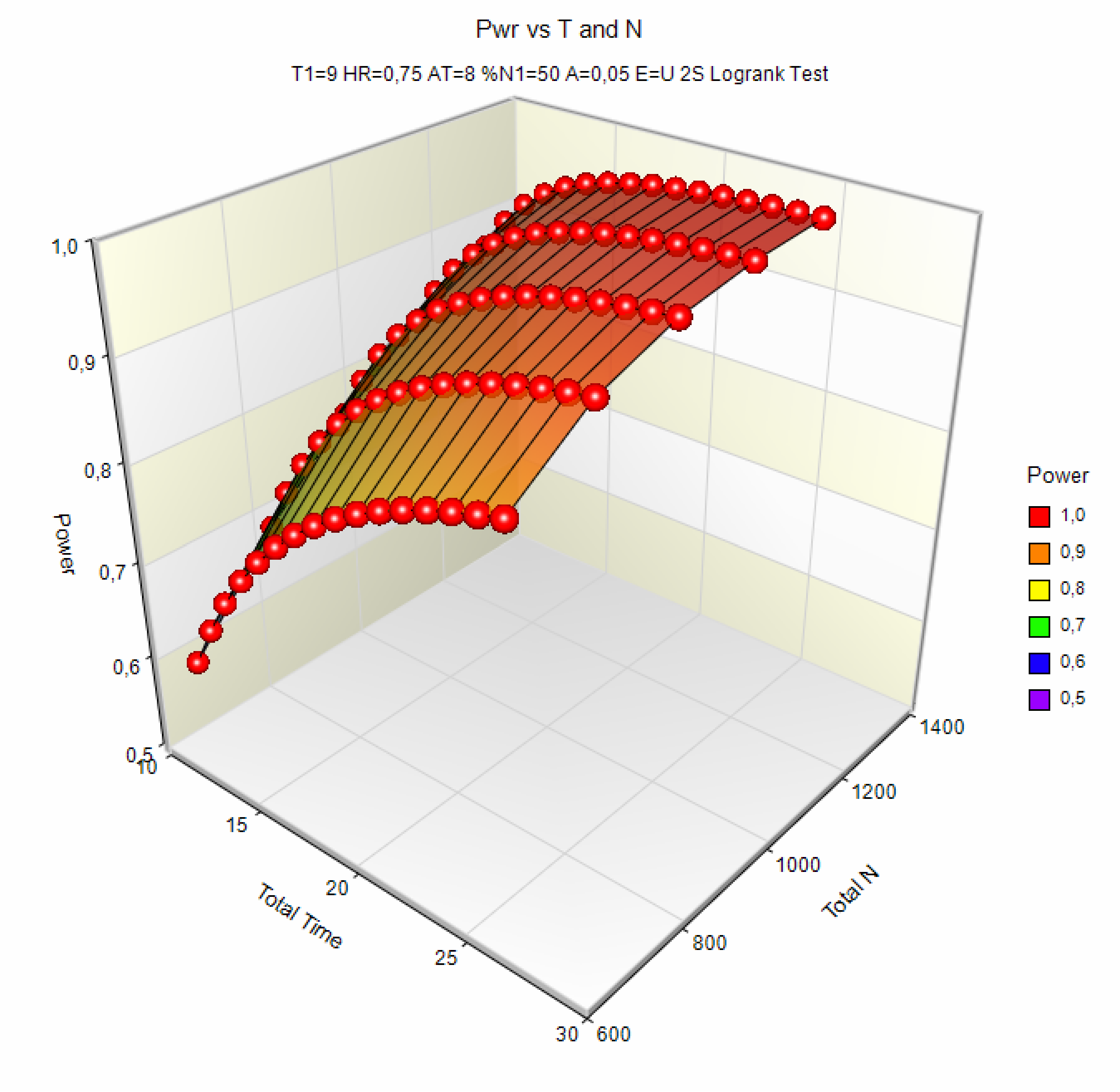

Logrank Tests

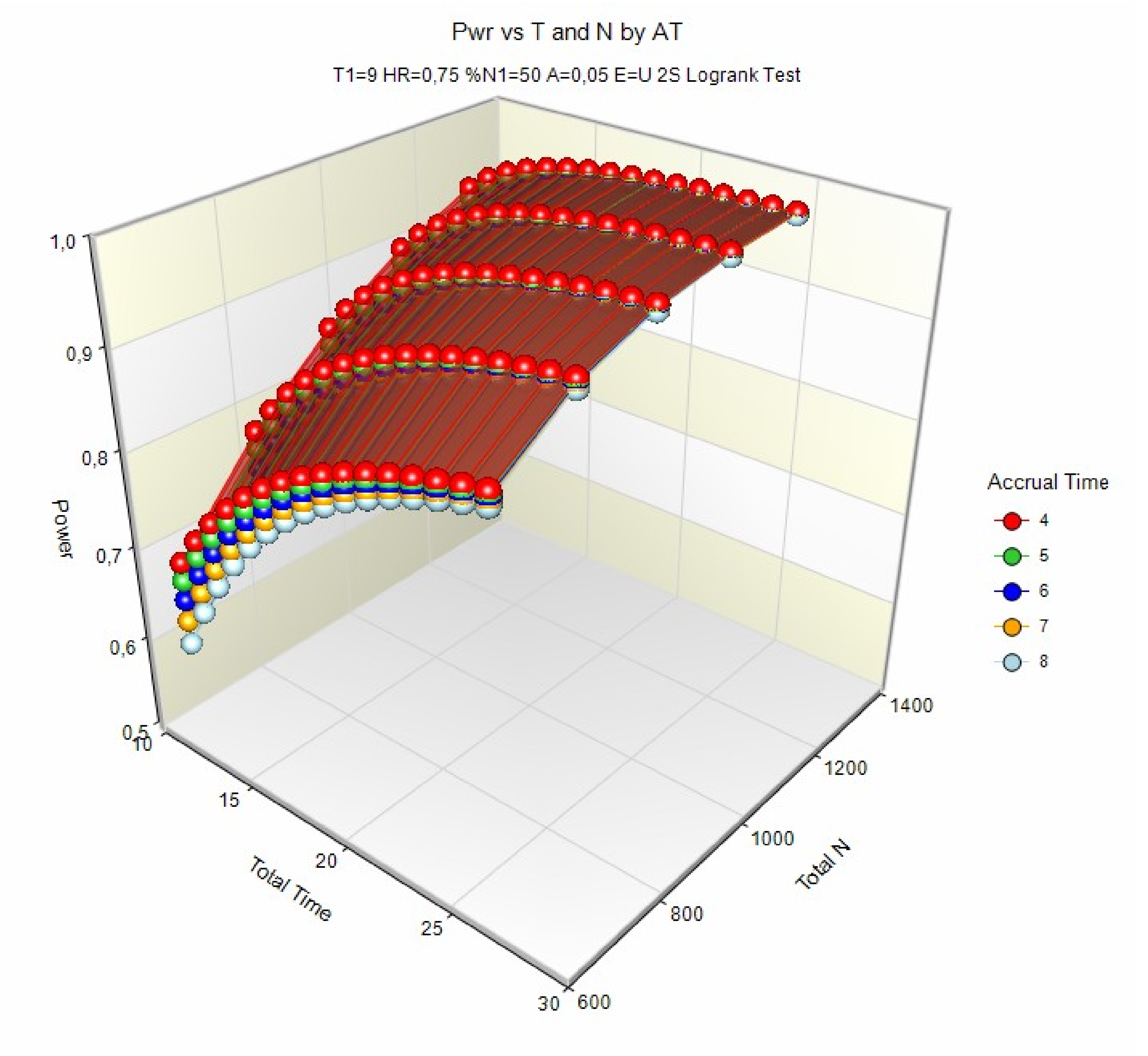

Logrank Tests

Procedure Input Settings

C:\Users\imbi\Documents\PASS 2024\Procedure Settings\Autosave\Logrank Tests - Autosaved 2026_9_3-11_0_5.t396

Design Tab

Solve For:	Power
Alternative Hypothesis:	Two-Sided
Alpha:	0,05
Group Allocation:	Equal (N1 = N2)
Sample Size Per Group:	320 to 640 by 80
Input Type:	Median Survival Time
T1 (Median Survival Time - Control):	9
Treatment Group Parameter:	HR (Hazard Ratio = T1/T2)
HR (Hazard Ratio = T1/T2 = h2/h1):	0,75
Accrual Time (Integers Only):	4 to 8 by 1
Accrual Pattern:	Uniform or Equal
Total Time (Integers Only):	11 to 26 by 1
Controls Lost:	0
Treatments Lost:	0
Controls Switch to Treatments:	0
Treatments Switch to Controls:	0

Options Tab

Number of Intervals within a Time Period:	2000
